# Metabolic Pathways for the Biosynthesis of Heptoses Used in the Construction of Capsular Polysaccharides in the Human Pathogen *Campylobacter jejuni*

**DOI:** 10.1021/acs.biochem.3c00390

**Published:** 2023-10-27

**Authors:** Dao Feng Xiang, Maggie Xu, Manas K. Ghosh, Frank M. Raushel

**Affiliations:** Department of Chemistry, Texas A&M University, College Station, Texas 77843, United States

## Abstract

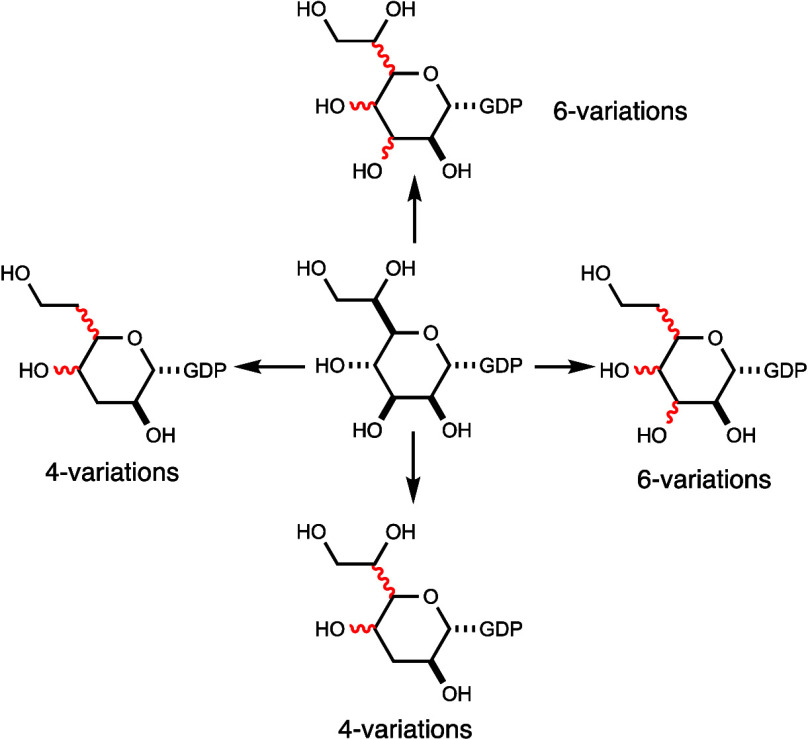

*Campylobacter jejuni* is the leading cause of food poisoning in North America. The exterior surface of this bacterium is coated with a capsular polysaccharide (CPS) that consists of a repeating sequence of 2–5 different carbohydrates that is anchored to the outer membrane. Heptoses of various configurations are among the most common monosaccharides that have been identified within the CPS. It is currently thought that all heptose variations derive from the modification of GDP-d-*glycero*-α-d-*manno*-heptose (GMH). From the associated gene clusters for CPS biosynthesis, we have identified 20 unique enzymes with different substrate profiles that are used by the various strains and serotypes of *C. jejuni* to make six different stereoisomers of GDP-6-deoxy-heptose, four stereoisomers of GDP-d-*glycero*-heptoses, and two stereoisomers of GDP-3,6-dideoxy-heptoses starting from d-sedoheptulose-7-phosphate. The modification enzymes include a C4-dehydrogenase, a C4,6-dehydratase, three C3- and/or C5-epimerases, a C3-dehydratase, eight C4-reductases, two pyranose/furanose mutases, and four enzymes for the formation of GMH from d-sedoheptulose-7-phosphate. We have mixed these enzymes in different combinations to make novel GDP-heptose modifications, including GDP-6-hydroxy-heptoses, GDP-3-deoxy-heptoses, and GDP-3,6-dideoxy-heptoses.

## Introduction

**Figure 1:**
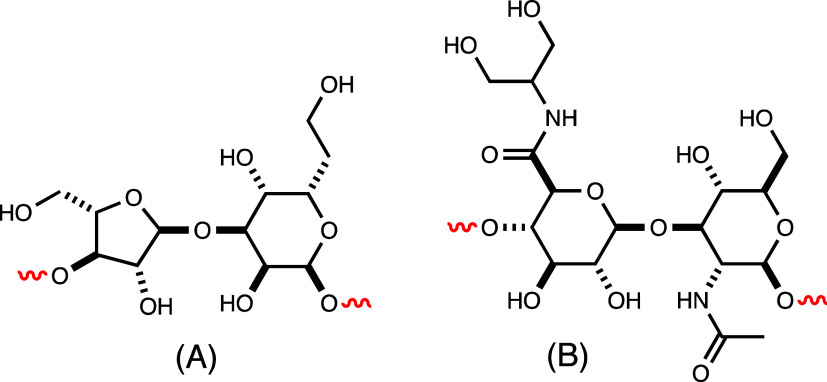
Structures of the repeating polysaccharides in the HS:15 and HS:19 serotypes of *C. jejuni*. (A) Repeating polysaccharide from the HS:15 serotype consists of l-arabinose and 6-deoxy-l-*gulo*-heptose. (B) Repeating polysaccharide from the HS:19 serotype consists of the serinol amide of d-glucuronate and *N*-acetyl-d-glucosamine.

*Campylobacter jejuni* is the leading cause of food poisoning in North America and Europe.^1^ In general, *C. jejuni* infections are predominantly attributed to the consumption of undercooked or contaminated poultry products.^2,3^*C. jejuni* has also been implicated in the acquisition of Guillain-Barré Syndrome (GBS), an autoimmune disorder that can result in muscle weakness and paralysis.^4,5^ Coating the exterior surface of *C. jejuni* is a capsular polysaccharide (CPS) that helps the bacterium evade the host immune system.^6^ The CPS consists of a repeating sequence of 2–5 monosaccharides that is anchored to the cell wall.^7,8^ Distinct strains and serotypes of *C. jejuni* can be differentiated from one another by changes in the composition and sequence of the monosaccharides that comprise the CPS. For example, in the HS:15 serotype, the repeating polysaccharide consists of an alternating sequence of l-arabinose and 6-deoxy-l-*gulo*-heptose, whereas in the HS:19 serotype, the CPS is composed of the serinol amide of d-glucuronate and *N*-acetyl-d-glucosamine as illustrated in Figure 1.^9,10^

**Figure 2:**
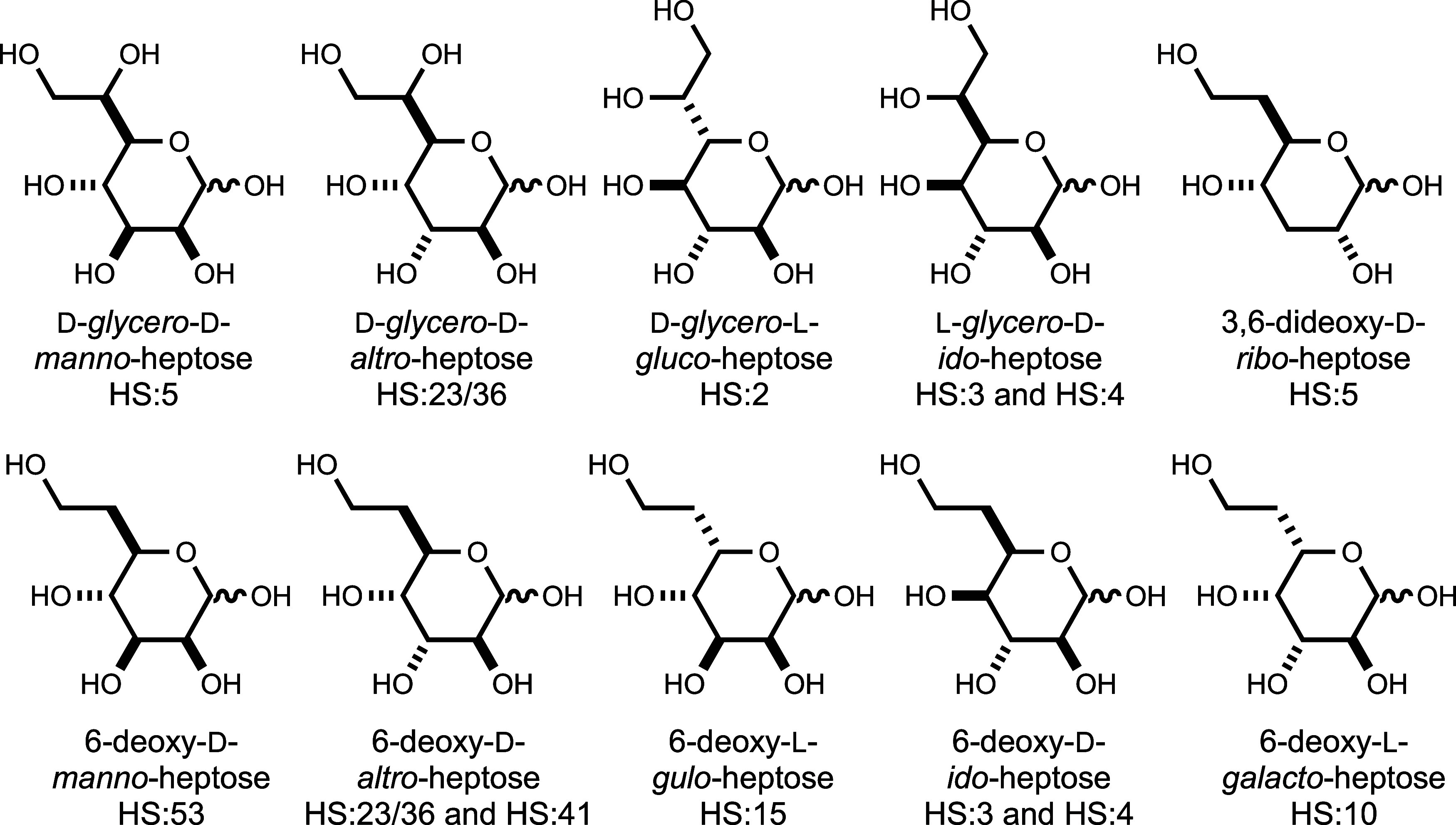
Structures of heptoses that have been reported to be found within various strains and serotypes of *C. jejuni*.

Heptoses are among the most common monosaccharides found within the capsular polysaccharides in *C. jejuni* whose chemical compositions have been structurally determined to date.^8^ From the CPS structures of the 12 serotypes that have been chemically characterized, the relatively uncommon heptoses have been found in 9 of these polysaccharides.^8^ Ten different heptoses have thus far been reported.^11−19^ These include stereochemical differences from C2 to C6 and deoxy modifications at C3 and C6. The specific monosaccharide moieties that have been reported are shown in Figure 2. It should also be noted that furanose configurations are observed for the 6-deoxy-l-*galacto*-heptose and 6-deoxy-d-*altro*-heptose moieties in the HS:10 and HS:41 serotypes, respectively.^15,18^

We have interrogated the gene clusters for 33 of the most common serotypes of *C. jejuni* to ascertain a more complete list of enzymes required for the biosynthesis of the heptoses needed for the assembly of the capsular polysaccharides. We have identified and functionally characterized 20 unique enzymes with different substrate profiles and the associated biosynthetic pathways that ultimately produce 14 different GDP-activated heptoses from 23 *C. jejuni* serotypes. We have also demonstrated that these 20 enzymes can be mixed in different combinations to make previously uncharacterized GDP-activated heptoses.

## Materials and Methods

### Materials

All materials used in this study were obtained from Sigma-Aldrich, Carbosynth, or GE Healthcare Biosciences, unless otherwise stated. Lysogeny broth (LB) and isopropyl-β-d-thiogalactopyranoside (IPTG) were purchased from Research Products International. HisTrap columns and Vivaspin 20 spin filters were obtained from Cytiva. The 3 and 10 kDa Nanosep spin filters were purchased from Pall Corporation (Port Washington, NY). Deuterium oxide was purchased from Cambridge Isotope Laboratories, Inc. *Escherichia coli* strain BL21-Gold (DE3) was obtained from New England Biolabs. α-Ketoglutarate was purchased from AK Scientific (Union City, CA). Ultraviolet spectra were recorded on a SpectraMax340 (Molecular Devices) ultraviolet–visible plate reader using 96-well Greiner plates. ^1^H NMR and ^1^H–^1^H COSY spectra were recorded on a Bruker Avance III 400 MHz system equipped with a broadband probe and sample changer. Mass spectrometry data were collected on a Thermo Scientific Q Exactive Focus system run in the negative-ion mode. GDP-d-*glycero*-α-d-*manno-*heptose (**5**) was synthesized and purified as described previously.^20^

### Protein Expression and Purification

The C4-dehydrogenase (Cj1427; UniProt id: Q0P8I7) from serotype HS:2, the C3-dehydratase (UniProt id: A0A0U3ALB0) from HS:5, the epimerases, and the associated C4-reductases from serotypes HS:2 (UniProt id: Q0P8I4 and Q0P8I6), and HS:3 (UniProt id: F2X702 and F2X701), HS:23/36 (UniProt id: Q6EF58 and Q5M6Q6), HS:33 (UniProt id: A0A0S2CGT2), HS:11 (UniProt id: A0A0U2QGV6 and A0A0U3ANW2), HS:15 (UniProt ids: A0A3Z9HSX9 and F2X7A7), HS:42 (UniProt ids: F2X7E5 and F2X7E4), and HS:51/HS:53 (UniProt id: Q5HSZ2) were expressed according to the procedures reported previously.^20−27^ In general, *E. coli* BL21(DE3) cells were transformed with the appropriate expression plasmids using MicroPulser (Bio-Rad). A 10 mL starter culture was used to inoculate a 1 L flask of lysogeny broth (LB) medium. Growth continued at 37 °C with shaking until the OD_600_ reached ∼0.6, followed by the addition of 1.0 mM IPTG to induce gene expression for 18 h at 22 °C. The cells were harvested by centrifugation at 7000*g* for 10 min at 4 °C, frozen in liquid N_2_, and stored at −80 °C.

Purification of the Cj1427 C4-dehydrogenase (HS:2), C3-dehydratase (HS:5), C3-epimerases (HS:3 and HS:23/36), C3/C5-epimerases (HS:2, HS:15, and HS:42), C5-epimerase (HS:11), and the various C4-reductases (HS:2, HS:3, HS:11, HS:15, HS:23/36, HS:33, HS:42 and HS:51/53) was conducted using the procedures reported previously.^20−27^ In a typical purification, ∼10 g of frozen cell paste was resuspended in 100 mL of buffer A (50 mM HEPES, pH 8.0, 250 mM KCl, 5.0 mM imidazole) supplemented with 0.1 mg/mL lysozyme, 0.05 mg/mL protease inhibitor cocktail powder, and 40 U/mL DNase I. The suspended cells were lysed by sonication (Branson 450 Sonifier), and the supernatant solution was collected after centrifugation at 10,000*g* for 30 min. The supernatant solution was loaded onto a prepacked 5 mL HisTrap column (Cytiva) and eluted with a linear gradient of buffer B (50 mM HEPES, pH 8.0, 250 mM KCl, 500 mM imidazole). Fractions containing the desired protein, as identified by sodium dodecyl sulfate–polyacrylamide gel electrophoresis, were combined and concentrated in a 20 mL spin filter with a 10 kDa molecular weight cutoff. The imidazole was removed from the protein solution by dialysis using buffer C (50 mM HEPES, pH 8.0, 250 mM KCl). Concentrations of the proteins were determined spectrophotometrically using computationally derived molar absorption coefficients at 280 nm, based on the protein sequences.^28^ The proteins were concentrated to 5–10 mg/mL, aliquoted, frozen in liquid N_2_, and stored at −80 °C.

### Isolation of GDP-d-*glycero*-4-keto-α-d-*lyxo*-heptose (**22**)

GDP-d-*glycero*-4-keto-α-d-*lyxo*-heptose (**22**) was made from GDP-d-*glycero*-α-d-*manno*-heptose (**5**) using Cj1427 C4-dehydrogenase (from serotype HS:2). Cj1427 (10 μM) was incubated with 4.0 mM GDP-d-*glycero*-α-d-*manno*-heptose (**5**) and 8.0 mM α-ketoglutarate in 4.0 mL of 50 mM HEPES, pH 8.0, for 18 h. The reaction mixture was subsequently loaded onto a prepacked 9 mm × 25 mm Dionex CarboPac PA1 column (Thermo Scientific) after the enzyme was removed using a 10 kDa molecular weight cutoff filter (Pall Corporation). The column was washed with water and then eluted using a linear gradient (0–80%) of 2 M ammonium acetate, pH 8.0, over four column volumes. Fractions of 1.0 mL were collected based on the UV spectra of the fractions (230–300 nm). The pooled fractions were analyzed using ESI-MS and then dried under vacuum.

### Synthesis and Isolation of GDP-d-*glycero*-α-d-*ido*-heptose (**28**)

To make GDP-d-*glycero*-α-d-*ido*-heptose (**28**) using the C4-reductase from serotype HS:3, a 1.0 mL reaction containing 4.0 mM GDP-d-*glycero*-α-d-*manno*-heptose (**5)**, 8.0 mM α-ketoglutarate, 10 mM acetaldehyde, 0.15 mM NADPH, and 3 units of aldehyde dehydrogenase was incubated with 10 μM Cj1427 (HS:2), 10 μM C3-epimerase (HS:3), and 10 μM C4-reductase (HS:3) in 50 mM HEPES, pH 8.0 for 18 h. The reaction mixture was then loaded onto a prepacked 1 mL HiTrap Q HP anion exchange column (GE Healthcare) after the enzymes were removed using a 10 kDa molecular weight cutoff filter (Pall Corporation). The column was washed with water and then eluted using a linear gradient (0–60%) of 500 mM ammonium bicarbonate, pH 8.0, over 60 column volumes. Fractions of 0.5 mL were collected based on the UV spectra of the fractions (230–300 nm) and then dried under vacuum.

### Synthesis and Isolation of Additional GDP-d-*glycero*-heptoses (**5, 26–30**)

The same procedures were adopted for making the appropriate products using the corresponding C4-reductases from serotypes HS:2 (**26**), HS:15 (**29**), HS:33 (**28**), and HS:51/HS:53 (**5**). Epimerases from the same serotype of each C4-reductase were used for each reaction, except for the reaction catalyzed by the C4-reductase from HS:51/HS:53, which does not need an epimerase prior to the reduction of C4. The product of the C4-reductase from HS:23/36 (**27**) was made in two steps. In the first step, GDP-d-*glycero*-α-d-*manno*-heptose (**5**) was oxidized to GDP-d-*glycero*-4-keto-α-d-*lyxo*-heptose (**22**) using Cj1427 C4-dehydrogenase (see above). In the second step, 4.0 mM purified Cj1427 product (**22**) was incubated with 10 μM C3-epimerase and 10 μM C4-reductase from HS:23/36 in the presence of 5.0 mM NADPH in 50 mM HEPES, pH 8.0 for 18 h. The reaction mixture was loaded onto a prepacked 9 mm × 25 mm Dionex CarboPac PA1 column (Thermo Scientific) after the proteins were removed using a 10 kDa molecular weight cutoff filter (Pall Corporation). The column was washed with water and then eluted using a linear gradient (0–80%) of 2 M NH_4_Ac (pH, 8.0) over four column volumes. Fractions of 1.0 mL were collected based on the UV spectra of the fractions (230–300 nm) and dried under vacuum. The product of the C4-reductase from HS:42 (**30**) was made using the same procedure as for the product of C4-reductase from HS:23/36 (**27**), except that 30 μM C4-reductase from HS:42 was used in the reaction and the incubation time was increased to 40 h.

The C4-reductase reactions were also conducted in D_2_O. Enzymes used in these reactions were initially solvent-exchanged into 50 mM ammonium bicarbonate buffer D_2_O (pD 8.0) using VivaSpin 3K spin filters, and then the reactions were conducted in 50 mM ammonium bicarbonate buffer in D_2_O (pD 8.0). The reaction buffers for the C4-reductases from HS:23/36 and HS:42 were exchanged into 50 mM ammonium acetate at pH 8.0. The purification procedures of the products were the same as for the products obtained in H_2_O.

All isolated C4-reductase products were analyzed using ESI-MS and then dried under vacuum using a freezer dryer (LABCONCO) and subsequently redissolved in D_2_O for ^1^H NMR and ^1^H–^1^H COSY spectra.

### Synthesis of Four GDP-d-*glycero*-3-deoxy-heptose Products (**34**–**38**)

GDP-d-*glycero*-4-keto-3-deoxy-d-*threo*-heptose (**33**) was made from **5** using Cj1427 and the C3-dehydratase from HS:5. GDP-d-*glycero*-α-d-*manno*-heptose (4.0 mM), 8.0 mM α-ketoglutarate, 0.25 mM PLP, and 10 mM l-glutamate were incubated with 10 μM Cj1427 and 10 μM C3-dehydratase (HS:5) in 50 mM HEPES, pH 8.0, for 18 h. The reaction mixture was subsequently loaded onto 9 mm × 25 mm Dionex CarboPac PA1 column after the enzymes were removed using a 10 kDa molecular weight cutoff filter (Pall Corporation). The column was washed with water and then eluted using a linear gradient (0–80%) of 2 M ammonium acetate, pH 8.0, over four column volumes. Fractions of 1.0 mL were collected based on the UV spectra of the fractions and dried under vacuum. The resulting product, GDP-d-*glycero*-4-keto-3-deoxy-*threo*-heptose (**33**), was analyzed using ESI-MS. In the following steps, a 1.0 mL reaction containing 4.0 mM compound **33**, 5.0 mM NADPH, 10 μM epimerase (if added), and 10 μM C4-reductase from four different serotypes (HS:53, HS:3, HS:2, and HS:11) was incubated in 50 mM HEPES, pH 8.0, for 18 h. The products were purified using the PA1 column as described above, analyzed by ESI-MS, dried under a vacuum, and dissolved in D_2_O for ^1^H NMR and ^1^H–^1^H COSY analysis. Four different GDP-d-*glycero*-3-deoxy-heptose products were made.

### Determination of Kinetic Constants for the C4-Reductases Used to Make GDP-d-*glycero*-heptose Products

The kinetic constants for the C4-reductases were determined by monitoring the oxidation of NADPH to NADP^+^ at 340 nm using a SpectraMax340 UV–visible plate reader. The substrates for six different C4-reductases were generated by incubating 4.0 mM GDP-d-*glycero*-α-d-*manno*-heptose (**5**) and α-ketoglutarate (8.0 mM) with Cj1427 (10 μM) in 50 mM HEPES (pH, 7.5) for 2 h. The Cj1427 was removed from the reaction mixture using a 10 kDa molecular weight cutoff filter (Pall Corporation). Total substrate concentrations used for the kinetic assays varied between 5 and 520 μM. The assays for the determination of the kinetic constants were conducted in 50 mM HEPES, pH 7.5, at 30 °C using 300 μM NADPH, 10 μM of either the C3-epimerse from HS:3 or the C3/C5-epimerase from HS:2, and different concentrations of the appropriate C4-reductase (0.1 μM HS:2 reductase; 0.05 μM HS:3 reductase; 1.0 μM HS:15 reductase; 0.1 μM HS:23/36 reductase; 0.03 μM HS:53 reductase, and 20 μM HS:42 reductase). The apparent values of *k*_cat_ and *k*_cat_/*K*_m_ were determined by fitting the initial velocity data to eq 1, where *v* is the initial velocity of the reaction, A is the substrate concentration, E_t_ is the enzyme concentration, *k*_cat_ is the turnover number, and *K*_m_ is the Michaelis constant. The concentration of the specific C4-reductase substrate (**22–25**) was determined using the measured equilibrium constant for the reactions catalyzed by the C3- and C3/C5-epimerase with substrate **22**.^23^
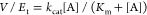
1

### Construction of Identity Matrices and Sequence Similarity Networks

The FASTA protein sequences for all genes were obtained from the UniProt Knowledgebase (UniProtKB). Identity matrices were generated from the FASTA-formatted sequences using the UniProt Align tool.^29^ All sequence similarity networks (SSN) were generated by submitting the FASTA-formatted sequences to the Enzyme Function Initiative-Enzyme Similarity Tool (EFI-EST).^30,31^ The SSNs were visualized into networks using Cytoscape 3.9.0.^32^

## Results and Discussion

### Biosynthesis of GDP-d-*glycero*-α-d-*manno*-Heptose (**5**)

**Figure 3:**
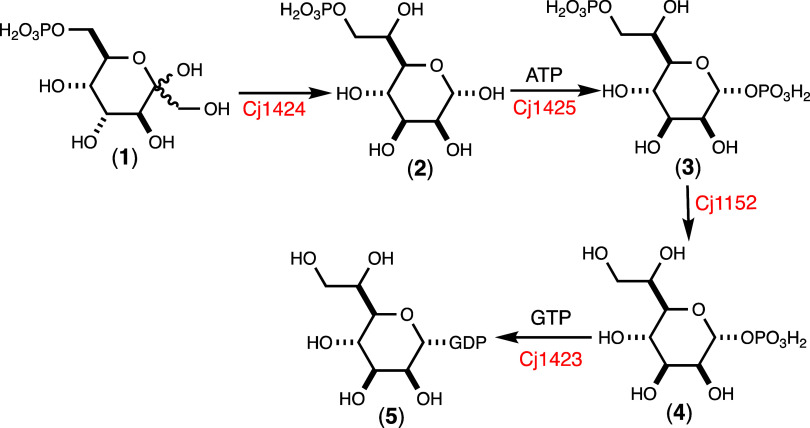
Biosynthetic pathway for the formation of GDP-d-*glycero*-α-d-*manno*-heptose (**5**) in serotype HS:2 of *C. jejuni*.

It is apparent that all of the heptoses that are currently found in the capsular polysaccharides of *C. jejuni* originate from GDP-d-*glycero*-α-d-*manno*-heptose (**5**). In *C. jejuni* NCTC 11168 (serotype HS:2), the biosynthesis is initiated by the isomerization of d-sedoheptulose-7-phosphate (**1**) to d-*glycero*-d-*manno*-heptose-7-phosphate (**2**) by d-sedoheptulose-7-P isomerase (Cj1424, GmhA).^20,33^ This product is subsequently phosphorylated at C1 with ATP by d-*glycero*-d-*manno*-heptose-7-P kinase (Cj1425, HddA) to form d-*glycero*-α-d-*manno*-heptose-1,7-bis-phosphate (**3**). This product is then dephosphorylated at C7 by an enzyme (Cj1152, GmhB) whose gene is not located within the cluster for capsular polysaccharide biosynthesis in *C. jejuni* to form d-*glycero*-α-d-*manno*-heptose-1-phosphate (**4**). The gene for this enzyme is found in a cluster for the ultimate biosynthesis of ADP-l-*glycero*-β-d-*manno*-heptose for lipopolysaccharide (LPS) biosynthesis.^33,34^ In the final step, GDP-d-*glycero*-α-d-*manno*-heptose (**5**) is formed by the reaction of **4** with GTP catalyzed by d-*glycero*-α-d-*manno*-heptose-1-P guanylyltransferase (Cj1423, HddC). The overall biosynthetic pathway for the HS:2 serotype is summarized in Figure 3.^20^

Examination of the corresponding gene clusters for CPS formation in 33 of the most common serotypes of *C. jejuni* reveals that the four enzymes needed for the construction of GDP-d-*glycero*-α-d-*manno*-heptose (**5**) are found in 23 different serotypes and thus heptoses populate a significant majority of the various CPS structures formed by *C. jejuni*. These serotypes include HS:2, HS:3, HS:4, HS:5, HS:8, HS:10, HS:11, HS:12, HS:15, HS:18, HS:23/36, HS:27, HS:29, HS:32/HS:58, HS:33, HS:40, HS:41, HS:42, HS:45, HS:51/HS:53, HS:52, HS:60, and HS:63. Comparison of the amino acid sequences of these enzymes from each of the 23 serotypes indicates an overall sequence identity of 78–100% and thus the catalytic properties are highly likely to be identical to one another (Tables S1–S3).

### Biosynthesis of GDP-6-Deoxy-heptoses

The most common type of heptose that is ultimately found in the capsular polysaccharides of *C. jejuni* are the 6-deoxy-heptoses (Figure 2). These heptoses are formed via a three-step reaction scheme that begins with the conversion of GDP-d-*glycero*-α-d-*manno*-heptose (**5**) to GDP-6-deoxy-4-keto-α-d-*lyxo*-heptose (**6**) catalyzed by GDP-d-*glycero*-d-*manno*-heptose 4,6-dehydratase.^35−37^ This enzyme is found in 19 of the 23 serotypes that can make GDP-d-*glycero*-α-d-*manno*-heptose (**5**). The 4,6-dehydratases include those identified from serotypes HS:3, HS:4, HS:5, HS:8, HS:10, HS:11, HS:12, HS:15, HS:18, HS:23/36, HS:29, HS:32/HS:58, HS:41, HS:42, HS:45, HS:51/HS:53, HS:52, HS:60, and HS:63. Sequence comparisons of the 19 4,6-dehydratases with one another demonstrate a pairwise identity of 89–98% (Table S4). The catalytic reaction mechanism for the 4,6-dehydratases involves an NAD^+^-dependent oxidation of C4, elimination of water from C5/C6, followed by the NADH-dependent conjugate addition of hydride to the β-carbon.^35−41^

**Figure 4:**
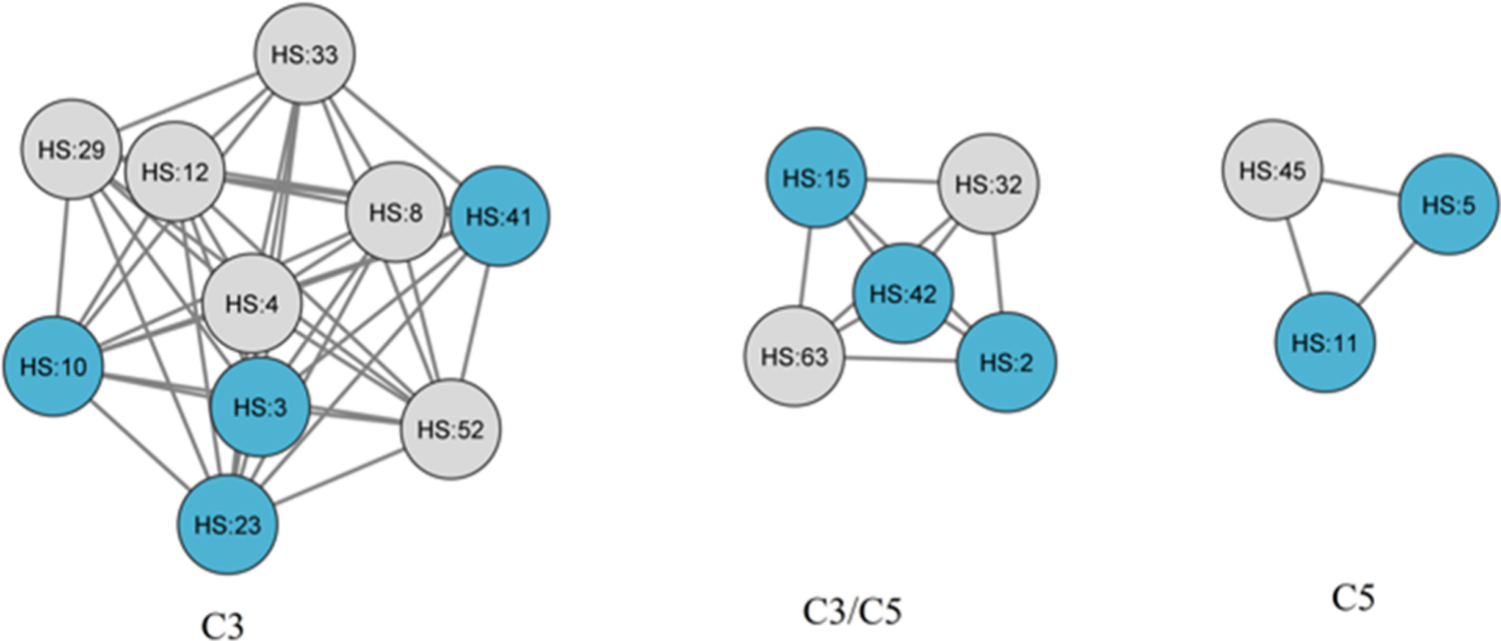
Sequence similarity network for the C3-, C5-, and C3/C5-epimerases at a sequence identity cutoff of 89%. The blue nodes represent enzymes that have been purified to homogeneity and whose reaction profiles were experimentally determined. Additional details are available in the text.

**Figure 5:**
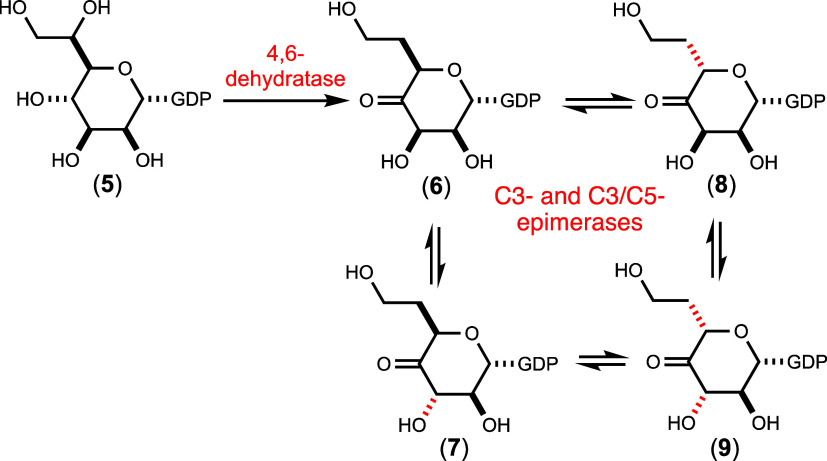
Reactions catalyzed by the 4,6-dehydratase (**5** to **6**), the C3-epimerase (**6** to **7**), and the C3/C5-epimerase (**6** to **7**, **8**, and **9**).

The C4-keto functional group in GDP-6-deoxy-4-keto-α-d-*lyxo*-heptose (**5**) renders the stereochemical configuration at C3 and C5 susceptible to racemization via an enolate intermediate that is formed by proton abstraction from either C3 and/or C5.^24,42−44^ Within the 33 common serotypes of *C. jejuni*, there are three types of C3- and C5-epimerases that have been identified thus far.^24,27,42,44^ The sequence similarity network (SSN) for the epimerases found within the gene clusters for heptose biosynthesis in *C. jejuni* is presented in Figure 4 at a sequence identity cutoff of 89%.^24,27^ The C3-epimerases have been shown to catalyze the epimerization of **6** to form GDP-6-deoxy-4-keto-α-d-*arabino*-heptose (**7**), whereas the C3/C5-epimerases have been shown to catalyze the epimerization of both C3 and C5 to generate an equilibrium mixture with GDP-6-deoxy-4-keto-β-l-*ribo*-heptose (**8**) and GDP-6-deoxy-4-keto-β-l-*xylo*-heptose (**9**), in addition to **7**.^24,42−44^ Ten serotypes (HS:3, HS:4, HS:8, HS:10, HS:12, HS:23/36, HS:29, HS:33, HS:41, and HS:52) contain a C3-epimerase (91–99% identical to one another), whereas five of the serotypes (HS:2, HS:15, HS:32/HS:58, HS:42, and HS:63) have the gene for the C3/C5-epimerase (81–98% identical to one another). Five serotypes (HS:18, HS:27, HS:40, HS:51/HS:53, and HS:60) do not contain either a C3 or C3/C5-epimerase and are thus highly likely to utilize either GDP-d-*glycero*-d-*manno*-heptose (**5**) or GDP-6-deoxy-d-*manno*-heptose (**10**) in their ultimate capsular polysaccharide. The third type of epimerase (C5-epimerase) is more specialized in that it has been shown to epimerize C5 of GDP-3,6-dideoxy-α-d-*threo*-heptose (**16**, *vida infra*). The C5-epimerases from HS:5, HS:11, and HS:45 are 88–97% identical to one another. This pathway, used for the biosynthesis of 3,6-dideoxy heptoses, will be discussed later. The reactions catalyzed by the 4,6-dehydratase and the C3 and C3/C5-epimerases are illustrated in Figure 5.

### Reaction Specificity of the C4-Reductases

**Figure 6:**
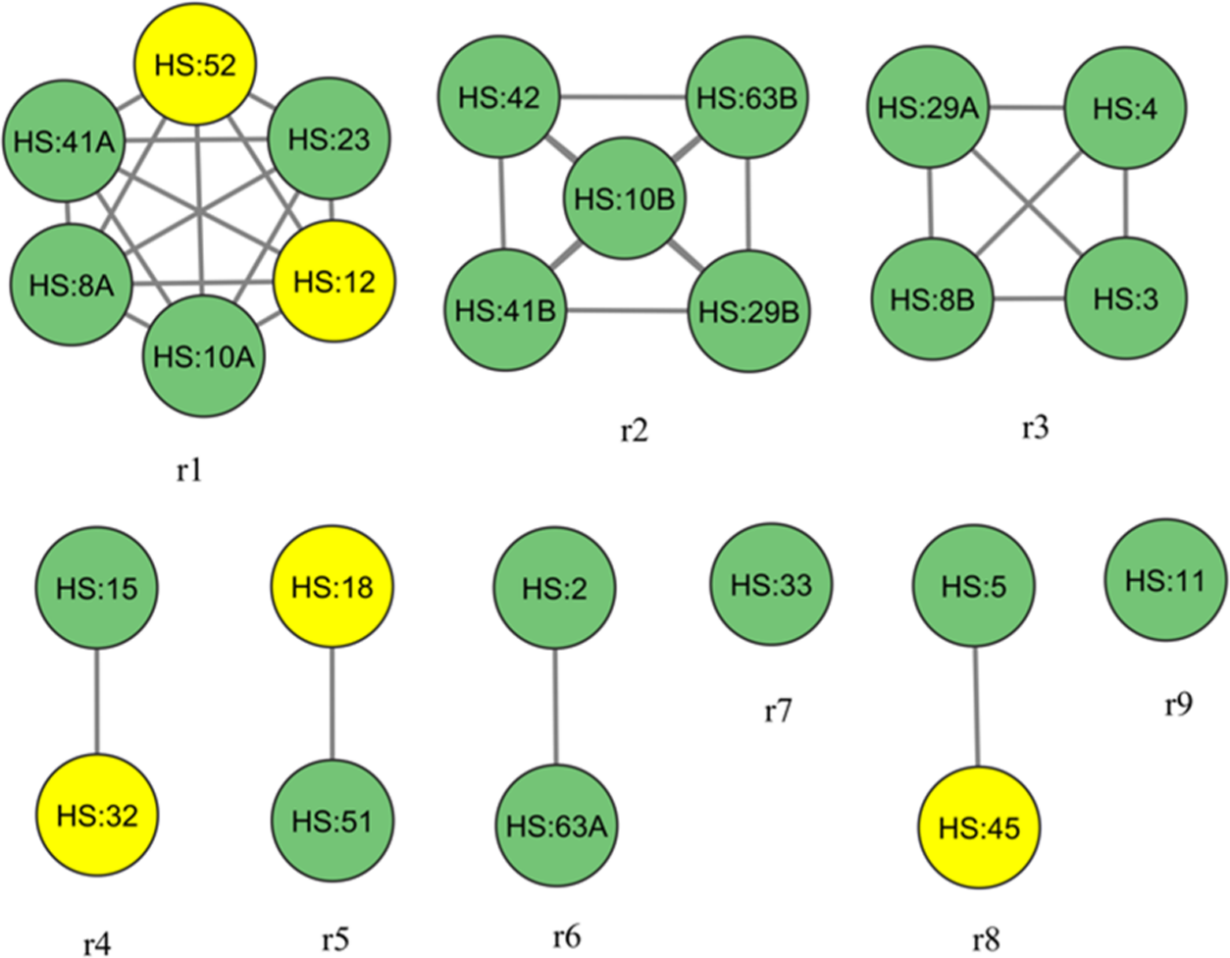
Sequence similarity diagram for 25 C4-reductases at a sequence identity cutoff of 92% that have been identified within the gene clusters for the biosynthesis of capsular polysaccharides from various serotypes of *C. jejuni*. The green nodes represent enzymes that have been purified to homogeneity, and their substrate and reaction profiles were determined experimentally. Additional details are available in the text.

After C4 has been oxidized and C3 or C5 has been epimerized, a C4-reductase is utilized to reduce the 4-keto group to the corresponding alcohol in either of the two possible stereochemical orientations. Within a 6-deoxy substrate core, there are a total of 8 possible products that differ in stereochemistry at C3, C4, and C5. Of the 8 possible products, C4-reductases have been identified within *C. jejuni* that will make 6 of these products. The isomers not observed are GDP-6-deoxy-β-l-*allo*-heptose and GDP-6-deoxy-α-d-*talo*-heptose.^25,26,42−44^ The sequence similarity diagram for the C4-reductases from *C. jejuni* at a sequence identity cutoff of 92% is presented in Figure 6. Twenty-five C4-reductases have been identified from the 33 most common serotypes of *C. jejuni*. From the SSN these C4-reductases cluster together into 9 separate groups. Twenty of the 25 known C4-reductases have been purified to homogeneity and their catalytic properties have been determined.^25,26,42−44^ It must be noted here that in five serotypes, there are two different C4-reductases contained within a single gene cluster. These include those from HS:8, HS:10, HS:29, HS:41, and HS:63. We have arbitrarily labeled one of these two genes/proteins as either an A-type or B-type depending on which gene appears nearer to the 5′-end of the cluster.^26^ Some of the B-type C4-reductases have additional catalytic properties that are discussed below.

**Figure 7:**
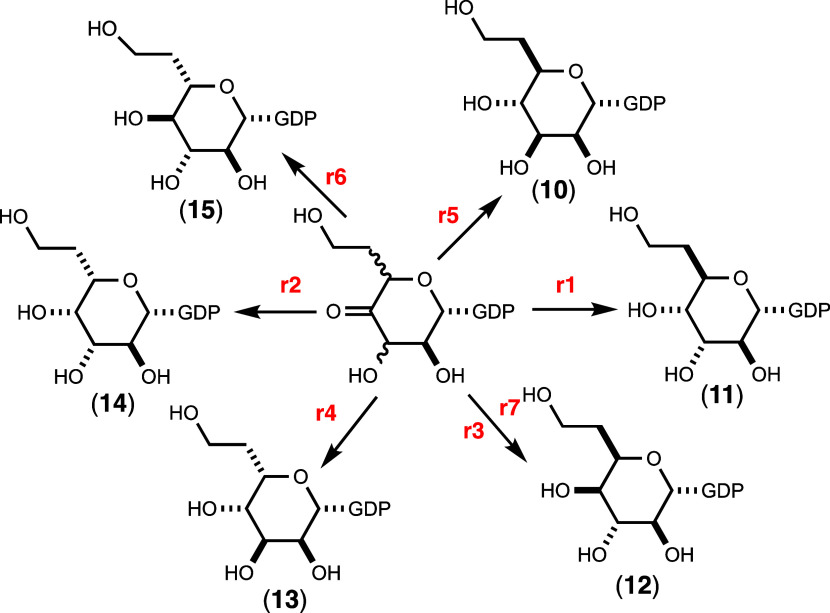
Product specificities for 7 groups of C4-reductases identified in the gene clusters for the biosynthesis of the capsular polysaccharides of *C. jejuni*. The labels (**r1** through **r7**) refer to the SSN for the C4-reductases that appears in Figure 6.

There are 20 serotypes of *C. jejuni* that contain at least one C4-reductase. Twelve of these serotypes exclusively make 6-deoxy-heptose products (HS:8, HS:10, HS:12, HS:15, HS:18, HS:32/HS:58, HS:29 HS:41, HS:42, HS:51/HS:53, HS:52, and HS:63), two will only make GDP-6-hydroxy-heptose products because they do not have a 4,6-dehydratase (HS:2 and HS:33), and three of them will make 3,6-dideoxy products (HS:5, HS:11, and HS:45). The remaining 3 serotypes can function to make both 6-deoxy and 6-hydroxy-heptose products (HS:3, HS:4, and HS:23/36). The product specificities for each of the nine groups of enzymes identified within the SSN of Figure 6 have been determined from the original chemical characterization of the capsular polysaccharides^8^ and from NMR analysis of the isolated products using purified enzymes.^25−27,42−44^ The enzymes in groups **r1**, **r2**, **r3**, **r4**, **r5**, **r6**, and **r7** catalyze the formation of GDP-6-deoxy-α-d-*altro*-heptose (**11**), GDP-6-deoxy-β-l-*galacto*-heptose (**14**), GDP-6-deoxy-α-d-*ido*-heptose (**12**), GDP-6-deoxy-β-l-*gulo*-heptose (**13**), GDP-6-deoxy-β-d-*manno*-heptose (**10**), GDP-6-deoxy-β-l-*gluco*-heptose (**15**), and GDP-6-deoxy-β-d-*ido*-heptose (**12**), respectively. The chemical structures of these products are listed in Figure 7.

### Epimerase Activity of Specific C4-Reductases

In the gene clusters for five of the most common serotypes (HS:8, HS:10, HS:29, HS:41, and HS:63), there are two different C4-reductases that have arbitrarily been denoted as A-type and B-type, depending on the specific location of the two genes within the gene cluster for CPS biosynthesis.^26^ Four of the B-type C4-reductases catalyze the formation of GDP-6-deoxy-β-l-*galacto*-heptose (**14**) and are found within the **r2** group of C4-reductases (Figure 6). In three of these serotypes (HS:10, HS:29, and HS:41), the associated epimerase will only catalyze the epimerization of C3 from compound **6**, and thus it was not initially apparent how product **14** could be formed.^26^ This ambiguity was solved when it was determined that all members of the **r2** group of C4-reductases can catalyze the formation of product **14** directly from substrate **6**, and thus these C4-reductases can also epimerize C3 and C5 prior to the reduction of C4.^26^ None of the other C4-reductases tested can epimerize either C3 or C5.^26^

### Biosynthesis of GDP-3,6-Dideoxy-heptoses

**Figure 8:**
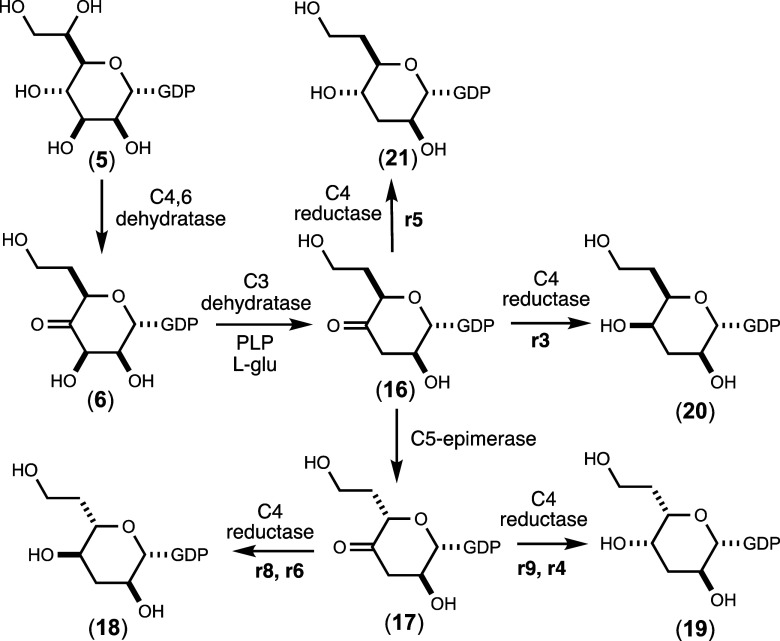
Biosynthetic pathway for the construction of GDP-3,6-dideoxy-heptoses in *C. jejuni.* Additional details are provided in the text.

It was previously reported that one of the two heptoses found in the HS:5 serotype was 3,6-dideoxy-d-*ribo*-heptose (Figure 2).^8,45,46^ The gene cluster for the biosynthesis of the CPS within the HS:5 serotype contains the three genes for the biosynthesis of GDP-d-*glycero*-α-d-*mann*o-heptose (**5**), a 4,6-dehydratase, a putative 3-dehydratase, and an epimerase and C4-reductase of unknown specificities. The apparent epimerization at C2 was of interest since none of the characterized enzymes within any of the *C. jejuni* serotypes was previously shown to catalyze the epimerization of C2 from a manno-heptose core structure. This issue was solved by the demonstration that the available enzymes from the HS:5 serotype actually synthesize GDP-3,6-dideoxy-β-l-*ribo*-heptose (**18**) rather than GDP-3,6-dideoxy-α-d-*ribo*-heptose.^27^ The newly established pathway for the biosynthesis of 3,6-dideoxy-heptoses in *C. jejuni* is presented in Figure 8.^27^

In the biosynthetic pathway for the construction of a GDP-3,6-dideoxy-heptose in the HS:5 serotype of *C. jejuni*, the 4,6-dehydratase catalyzes the formation of **6** from **5**.^27^ The putative C3-dehydratase (HS5.12) was shown to catalyze the conversion of **6** to GDP-3,6-dideoxy-4-keto-d-*threo*-heptose (**16**). This reaction was shown to require pyridoxal phosphate (PLP) and l-glutamate with the formation of α-ketoglutarate and ammonia.^27^ In the next step, the epimerase (HS5.14) catalyzes the racemization of C5 to form GDP-3,6-dideoxy-β-l-*erythro*-heptose (**17**). In the final step, the C4-reductase (HS5.13) catalyzes the NADPH-dependent reduction of **17** to GDP-3,6-dideoxy-β-l-*ribo*-heptose (**18**). The identical product was formed by using the C4-reductases from the HS:2 (**r6**) serotype.

Within the 23 serotypes of *C. jejuni* that are known to make GDP-d-*glycero*-α-d-*manno*-heptose (**5**), there are four serotypes that contain a putative 3-dehydratase within the gene cluster for CPS formation. These include HS:5, HS:11, HS:45, and HS:60. A sequence comparison of the putative C3-dehydratases from these four serotypes shows that the sequence identity among the four enzymes varies from 94 to 96% (Figure S7), indicating that each of these enzymes catalyzes the same conversion of **6** to **16** (Figure 8). Moreover, the C5-epimerases from serotypes HS:5, HS:11, and HS:45 are 88–97% identical to one another (oddly, the HS:60 serotype does not appear to contain either a C5-epimerase or a C4-reductase). The C4-reductases in the HS:5 and HS:45 serotypes are 96% identical to one another, and thus each of these serotypes makes the same ultimate product, GDP-3,6-dideoxy-β-l-*ribo*-heptose (**18**). The C4-reductase from HS:5 has been shown to make compound **18** from compound **17**.^27^ However, C4-reductase from the HS:11 serotype is different. The sequence identities in comparison with the C4-reductases from the HS:5 and HS:45 serotypes are 44 and 43%, respectively, and thus, the ultimate product from this serotype is different. Structural determination of this product demonstrates the formation of GDP-3,6-dideoxy-β-l-*xylo*-heptose (**19**).^27^ The reactions are summarized in Figure 8.

### Biosynthesis of GDP-d-*glycero*-Heptoses

**Figure 9:**
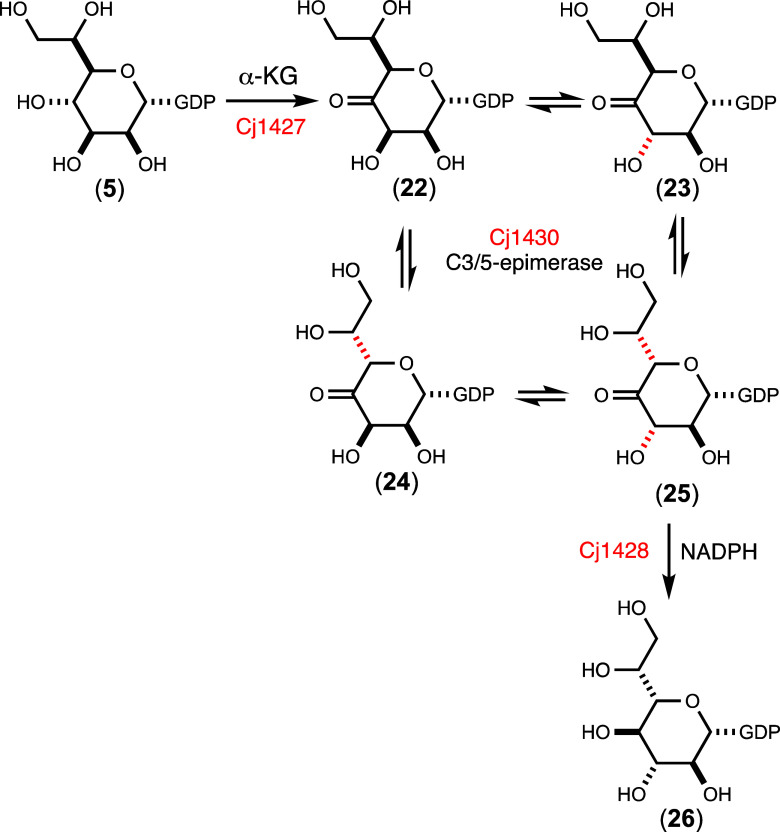
Biosynthesis of GDP-d-*glycero*-β-l-*gluco*-heptose (**26**) in the HS:2 serotype of *C. jejuni*.^23^

In addition to the 6-deoxy and 3,6-dideoxy heptoses found in the various capsular polysaccharides of *C. jejuni*, there are also heptoses that have retained the hydroxyl group at C6. These include d-*glycero*-l-*gluco*-heptose from the HS:2 serotype and d-*glycero*-d-*altr*o-heptose from HS23/36.^7,17^ These heptoses are also synthesized from GDP-d-*glycero*-α-d-*manno*-heptose (**5**). The pathway for the biosynthesis of GDP- d-*glycero*-β-l-*gluco*-heptose (**26**) in the HS:2 serotype is presented in Figure 9.^23^ In the first step, the GDP-*manno*-heptose (**5**) is oxidized at C4 by a C4-dehydrogenase. Initially, this transformation eluded characterization since the purified enzyme contains a tightly bound NADH that cannot be removed by dialysis and is not easily displaced by NAD^+^.^22,42^ Since the oxidation of C4 requires NAD^+^, the as-isolated enzyme is inactive, even in the presence of added NAD^+^.^21,22,42^ However, the tightly bound NADH can be oxidized by the addition of α-ketoglutarate, which is subsequently reduced to l-2-hydroxyglutarate.^21,22^ The enzyme functions as a ping-pong dehydrogenase that requires the presence of α-ketoglutarate and substrate **5**, which is converted to GDP-d-*glycero*-4-keto-α-d-*lyxo*-heptose (**22**). In the next step, the C3/C5-epimerase (Cj1430) catalyzes the epimerization of C3 and C5 to form a mixture of GDP-d-*glycero*-4-keto-α-d-*arabino*-heptose (**23**), GDP-d-*glycero*-4-keto-β-l-*ribo*-heptose (**24**), and GDP-d-*glycero*-4-keto-β-l-*xylo*-heptose (**25**). In the final step, the C4-reductase (Cj1428) catalyzes the NADPH-dependent reduction of **25** to GDP-d-*glycero*-β-l-*gluco*-heptose (**26**).

**Figure 10:**
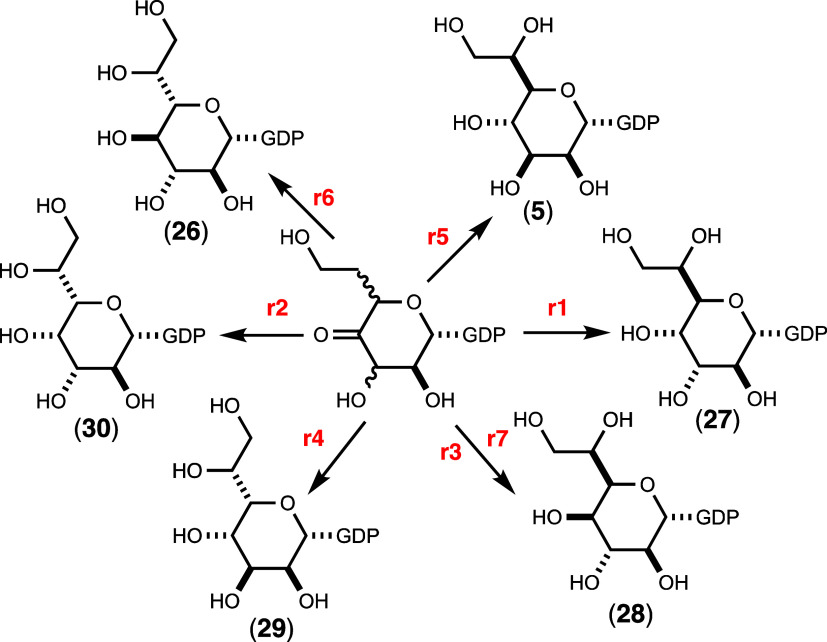
Product specificities for 7 groups of C4-reductases identified in the gene clusters for the biosynthesis of the capsular polysaccharides of *C. jejuni*. The labels (**r1** through **r7**) refer to the SSN for the C4-reductases that appear in Figure 6.

Within the other serotypes of *C. jejuni* that are known to catalyze the formation of GDP-d-*glycero*-α-d-*manno*-heptose (**5**) there are four others that possess a C4-dehydrogenase essentially identical to that of Cj1427 from serotype HS:2. These serotypes include HS:3, HS:4, HS:23/36, and HS:33 with an average sequence identity of 97–99% among the five enzymes (Table S8). Other stereochemical variations can be constructed via the same reaction scheme illustrated for the biosynthesis of GDP-d-*glycero*-4-keto-β-l-*gluco*-heptose (**26**) where a specific C4-reductase selectively reduces C4 in **23**, **24**, or **25** formed from the action of either a C3 or C3/C5-epimerase on compound **22** (Figure 10). However, this scenario does not currently explain the biosynthesis of l-*glycero*-d-*ido*-heptose in the HS:3 and HS:4 serotypes. In the HS:3 and HS:4 serotypes, the gene cluster for heptose biosynthesis contains a C4-dehydrogenase, a C4/6-dehydratase, a C3-epimerase, and a single C4-reductase (see Figures S2 and S3). The CPS structure in these two serotypes is reported to contain both l-*glycero*-d-*ido*-heptose and 6-deoxy-d-*ido*-heptose.^17^ Thus, we can conclude that the C3-epimerase and the C4-reductase are used in the biosynthesis of both products, that the C4,6-dehydratase is used for the 6-deoxy-product, and that the C4-dehydrogenase is used for the 6-hydroxy product. However, it is not at all clear how the stereochemistry at C6 is racemized from that found in GDP-d-*glycero*-d-*manno*-heptose (**5**).

Mechanistically, the stereochemistry at C6 can be isomerized by at least four possible routes. From the 4-keto intermediate (**22**), an enzyme could catalyze dehydration/rehydration of H_2_O from C5/C6. This would involve the formation of an α/β-unsaturated ketone, followed by the addition of the OH group on the opposite side of the transient double bond. Alternatively, from intermediate **22**, an enzyme could catalyze a retro-aldol reaction at C5/C6 with the transient formation of glycolaldehyde from C6/C7 and an enolate intermediate from C5/C4. The third possibility is oxidation/reduction of the hydroxyl group at C6 from either **5** or GDP-d-*glycero*-α-d-*ido*-heptose (**28**). The fourth possibility would involve the oxidation of C7 followed by deprotonation of the hydrogen at C6 and reprotonation of the enolate intermediate. However, thus far we have been unable to identify any enzyme that appears capable of epimerizing C6 within the gene cluster for the biosynthesis of the capsular polysaccharides within the HS:3 or HS:4 serotypes. These mechanistic possibilities are highlighted in Scheme S1. In a related system, the Tanner group has shown that ADP-l-*glycero*-β-d-*manno*-heptose is formed from ADP-d-*glycero*-β-d-*manno*-heptose via the oxidation/reduction of C6.^47^

### Biosynthesis of GDP-Heptofuranosides

**Figure 11:**
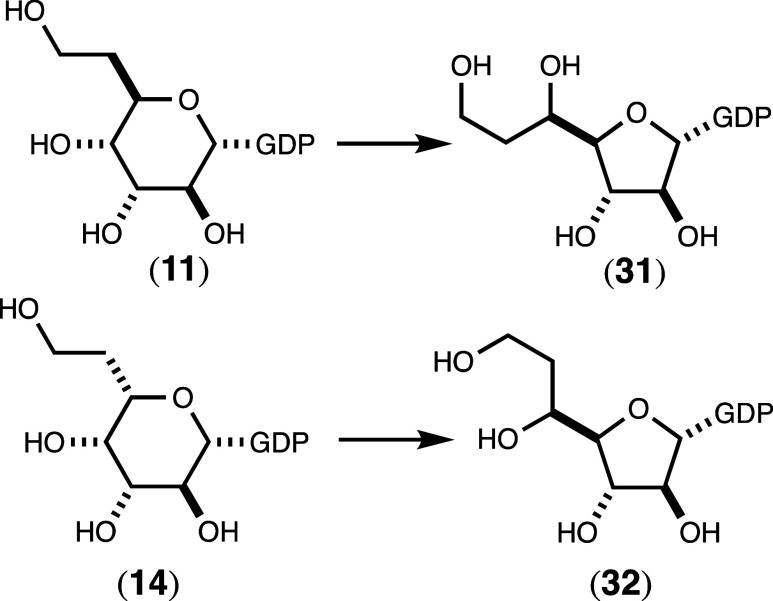
Reactions catalyzed by the pyranose/furanose mutases from serotypes HS:10 (HS10.16) and HS:41 (HS41.15).

In the capsular polysaccharides of the HS:10 and HS:41 serotypes, the heptoses are found as furanosides rather than the more common pyranosides.^15,18^ In the gene clusters for both serotypes, a pyranoside/furanoside mutase can be identified. Enzymes of this type catalyze an FADH_2_-dependent conversion of an NDP-pyranoside to an NDP-furanoside.^48^ In the HS:10 serotype, the gene is HS10.16 (Figure S6), and in the HS:41 serotype, the gene is HS41.15 (Figure S17). Neither of these enzymes has been experimentally characterized to date, but there is little doubt that these two enzymes are responsible for the conversion of their respective GDP-heptopyranoside to the corresponding GDP-heptofuranoside. The only other serotype that we can identify that possesses a pyranoside-furanoside mutase is HS:60 (HS60.11). However, the ultimate product within the HS:60 serotype cannot be determined since a C4-reductase has not been identified in the gene cluster for CPS formation. In any event, the sequence identity matrix (Table S9) indicates that HS10.16 and HS41.15 are 88% identical to one another, and each is 62% identical to HS60.11. It is interesting to recall that there are two C4-reductases in the HS:10 and HS:41 serotypes. Both serotypes have the potential to make GDP-6-deoxy-β-l-*galacto*-heptose (**14**) and GDP-6-deoxy-α-d-*altro*-heptose (**11**) and that a polyG tract at the 3′-end of the gene for the first C4-reductase (dubbed A-type) most likely dictates which of the two C4-reductases is ultimately expressed in a functional form.^26^ In serotype HS:10, the final product is 6-deoxy-β-l-*galacto*-heptofuranoside,^15^ whereas in HS:41, it is 6-deoxy-α-d-*altr*o-heptofuranoside.^18^ Since the pyranose/furanose mutases found in the HS:41 and HS:10 serotypes are ∼88% identical in sequence to one another, it is highly likely that both enzymes have the same substrate profile, and thus either one can convert **11** and **14** to GDP-6-deoxy-α-d-*altro*-heptofuranose (**31**) and GDP-6-deoxy-β-l-*galacto*-heptofuranoside (**32**), respectively. These transformations are presented in Figure 11.

### Enzymes Utilized for Heptose Biosynthesis in *C. jejuni*

Currently, there are at least 23 different strains and serotypes of *C. jejuni* that are known to synthesize heptoses for use in the construction of capsular polysaccharides. From an examination of the gene clusters for CPS formation, we have identified 20 enzymes that are used in the production of 14 different GDP-activated heptoses including four heptoses (**5,26–28**), six 6-deoxy-heptoses (**10**–**15**), two 3,6-dideoxy-heptoses (**18, 19**), and two 6-deoxy-heptofuranosides (**31, 32**). In addition to these compounds, an additional 13 intermediates (**2–4, 6–9, 16–17**, and **22**–**25**) have been identified and characterized. The enzymes needed for these transformations include eight C4-reductases, three epimerases, two dehydratases, two pyranose/furanose mutases, one C4-dehydrogenase, and four enzymes for the biosynthesis of GDP-d-*glycero*-d-*manno*-heptose (**5**) from sedoheptulose-7-phosphate (**1**). The specific enzymes are compiled in Table 1, and the expected final products for each of the 23 serotypes are provided in Table 2. The gene clusters for GDP-heptose biosynthesis for each of the *C. jejuni* serotypes known to make heptoses and the associated enzyme-catalyzed reactions are provided in Figures S1–S23.

**Table 1:** Reactions Catalyzed by the Enzymes Required for the Synthesis of GDP-Heptoses in *C. jejuni*

enzyme	primary reaction	alternative reaction
d-sedoheptulose-7-P	**1** → **2**	
1,2-isomerase		
d-*glycero*-d-*manno*-heptose-7-P	**2** → **3**	
1-kinase		
d-*glycero*-d-*manno*-heptose-1,7-*bis*-P	**3** → **4**	
7-phosphatase		
d-*glycer*o-d-*manno*-heptose-1-P guanosyltransferase	**4** → **5**	
GDP-d-*glycero*-α-d-*manno*-heptose	**5** → **22**	
4-dehydrogenase		
GDP-d-*glycero*-α-d-*manno*-heptose	**5** → **6**	
4,6-dehydratase		
GDP-4-keto-α-d-*lyxo*-heptose	**6** → **7**	
3-epimerase	**22** → **23**	
GDP-4-keto-α-d-*lyxo*-heptose	**6** → **7**, **8**, **9**	**33** → **36**
3,5-epimerase	**22** → **23**, **24**, **25**	**16** → **17**
GDP-β-l-*gluco*-heptose synthase	**9** → **15**	**17** → **18**
	**25** → **26**	**36** → **37**
GDP-β-l-*galacto*-heptose synthase	**9** → **14**	**25** → **30**
	**6** → **14**	
GDP-β-l-*gulo*-heptose synthase	**8** → **13**	**24** → **29**
		**17** → **19**
GDP-α-d-*manno*-heptose synthase	**6** → **10**	**22** → **5**
		**16** → **21**
		**33** → **34**
GDP-α-d-*altro*-heptose synthase	**7** → **11**	
	**23** → **27**	
GDP-α-d-*ido*-heptose synthase	**7** → **12**	**16** → **20**
	**23** → **28**	**33** → **35**
GDP-6-deoxy-α-d-4-keto-*lyxo*-heptose	**6** → **16**	**22** → **33**
3-dehydrase		
GDP-3,6-dideoxy-α-d-4-keto-*threo*-heptose	**16** → **17**	**33** → **36**
5-epimerase		
GDP-3,6-dideoxy-β-l-*ribo*-heptose synthase	**17** → **18**	
GDP-3,6-dideoxy-β-l-*xylo*-heptose synthase	**17** → **19**	**36** → **38**
GDP-6-deoxy-heptopyranoside mutase	**11** → **31**	
	**14** → **32**	
GDP-6-deoxy-heptopyranoside mutase	undetermined	
HS:60 (UniProt id: A0A0S2CFE4)		

**Table 2:** GDP-Heptose Products from Various *C. jejuni* Serotypes

serotype	GDP-monosaccharide	structure
HS:2	GDP-d-*glycero*-β-l-*gluco*-heptose	**26**
HS:3	GDP-6-deoxy-α-d-*ido*-heptose	**12**
	GDP-d-*glycero*-α-d-*ido*-heptose^a^	**28**
HS:4	GDP-6-deoxy-α-d-*ido*-heptose	**12**
	GDP-d-*glycero*-α-d-*ido*-heptose^b^	**28**
HS:5	GDP-d-*glycero*-α-d-*manno*-heptose	**5**
	GDP-3,6-dideoxy-α-l-*ribo*-heptose^c^	**18**
HS:8	GDP-6-deoxy-α-d-*ido*-heptose	**12**
	GDP-d-*glycero*-α-d-*altro*-heptose	**11**
HS:10	GDP-d-*glycero*-α-l-*galacto*-heptose	**14**
	GDP-d-*glycero*-α-d-*altro*-heptose	**11**
HS:11	GDP-3,6-dideoxy-β-l-*xylo*-heptose	**19**
HS:12	GDP-6-deoxy-α-d-*altro*-heptose	**11**
HS:15	GDP-6-deoxy-β-l-*gulo*-heptose	**13**
HS:18	GDP-6-deoxy-α-d-*manno*-heptose	**10**
HS23/36	GDP-6-deoxy-α-d-*altro*-heptose	**11**
	GDP-d-*glycero*-α-d-*altro*-heptose	**27**
HS:27	GDP-d-*glycero*-α-d-*manno*-heptose	**5**
HS:29	GDP-6-deoxy-α-d-*ido*-heptose	**12**
	GDP-6-deoxy-β-l-*galacto*-heptose	**14**
HS:32/HS:58	GDP-6-deoxy-β-l-*gulo*-heptose	**13**
HS:33	GDP-d-*glycero*-α-d-*ido*-heptose	**28**
HS:40	GDP-d-*glycero*-α-d-*manno*-heptose	**5**
HS:41	GDP-6-deoxy-β-l-*galacto*-heptose	**14**
	GDP-6-deoxy-α-d-*altro*-heptose	**11**
HS:42	GDP-6-deoxy-β-l-*galacto*-heptose	**14**
HS:45	GDP-3,6-dideoxy-β-l-*ribo*-heptose	**18**
HS:51/HS:53	GDP-6-deoxy-α-d-*manno*-heptose	**10**
HS:52	GDP-6-deoxy-α-d-*altro*-heptose	**11**
HS:60	unknown	unknown
HS:63	GDP-6-deoxy-β-l-*gluco*-heptose	**15**
	GDP-6-deoxy-β-l-*galacto*-heptose	**14**

a The reported heptose in the HS:3 serotype is l-*glycero*-d-*ido*-heptose.^13^

b The reported heptose in the HS:4 serotype is l-*glycero*-d-*ido*-heptose.^14^

c The reported heptose in the HS:5 serotype is 3,6-dideoxy-d-*ribo*-heptose.^8^

### *In Vitro* Enzymatic Synthesis of Additional GDP-Heptoses

The 20 enzymes identified from the various serotypes of *C. jejuni* can be exploited to construct GDP-activated heptoses that have not previously been made. Thus far, the only GDP-d-*glycero*-heptoses that have been synthesized using enzymes from *C. jejuni* are GDP-d-*glycero*-α-d-*manno*-heptose (**5**) and GDP-d-*glycero*-β-l-*gluco*-heptose (**26**).^20,34,36,37^ Additional products can potentially be made starting from **5** and the addition of the C4-dehydrogenase, C3- or C3/C5-epimerase, and the appropriate C4-reductase as originally illustrated in Figure 9 for the preparation of GDP-d-*glycero*-β-l-*gluco*-heptose (**26**). Using this procedure, GDP-d-*glycero*-α-d-*altro*-heptose (**27**), GDP-d-*glycero*-α-d-*ido*-heptose (**28**), GDP-d-*glycero*-β-l-*gulo*-heptose (**29**), and GDP-d-*glycero*-β-l-*galacto*-heptose (**30**) were isolated and chemically characterized by NMR and ESI-MS. In each case, the negative-ion ESI-MS spectra indicated an *m*/*z* for the M-1 anion of 634.08, compared to a calculated value of 634.08. When the reactions were conducted in D_2_O, the observed *m*/*z* values for the isolated products reflected an increase in mass due to deuterium solvent exchange at C3 and/or C5. For compound **5** (HS:53), there was no change in a *m*/*z* of 634.08 since no epimerase was used. For compounds **27** (HS:23/36) and **28** (HS:3 and HS:33), the observed *m*/*z* was 635.09, reflecting the required solvent exchange at C3. For compounds **26** (HS:2), **29** (HS:15), and **30** (HS:42), the observed *m*/*z* was 636.09, reflecting solvent exchange at both C3 and C5. The ^1^H NMR and ^1^H–^1^H COSY spectra are found in Figures S24–S36. The isolated yields and mass spectral data for the compounds specifically made for this investigation are listed in Table S10. The ESI-MS spectra are presented in Figures S41–S50. Compounds **5** and **26** have previously been characterized, but compounds **27**, **28**, **29**, and **30** are produced for the first time using enzymes from *C. jejuni*. The kinetic constants for the reduction of the appropriate GDP-d-*glycero*-4-keto-heptose (**22**–**25**, Figure 9) by the C4-reductases are presented in Table 3. In general, the values are similar to those previously determined for the reduction of the appropriate GDP-6-deoxy-4-keto-heptose by these same enzymes, except for the C4-reductase from HS:42 where the value of *k*_cat_ is approximately 50-fold reduced.^25^ Apart from the C4-reductase from serotype HS:42, these reductases are somewhat tolerant of whether a hydroxyl group is present at C6 of their respective substrates. Unfortunately, we did not have access to potential substrates with an *S*-stereochemistry (l-configuration) at C6.

**Table 3:** Steady-State Kinetic Parameters for the C-4 Dehydrogenases Used to Make Six GDP-d-*glycero*-Heptoses

C4-reductase (serotype)	reaction	*k*_cat_ (s^–1^)	*K*_m_ (μM)	*k*_cat_/*K*_m_ (M^–1^ s^–1^)
HS-2	**25** → **26**	3.7 ± 0.1	52 ± 4	(7.2 ± 0.5) × 10^4^
HS-3	**23** → **28**	9.4 ± 0.4	13 ± 2	(7.1 ± 0.8) × 10^5^
HS-15	**24** → **29**	0.29 ± 0.01	30 ± 4	(9.7 ± 1.1) × 10^3^
HS-23/36	**23** → **27**	2.5 ± 0.1	11 ± 2	(2.3 ± 0.1) × 10^5^
HS-42	**25** → **30**	0.010 ± 0.001	150 ± 12	(6.6 ± 0.8) × 10^1^
HS-53	**22** → **5**	7.0 ± 0.3	53 ± 6	(1.3 ± 0.2) × 10^5^

### *In Vitro* Enzymatic Synthesis of Additional GDP-3,6-Dideoxy-heptoses

In Figure 8, we have outlined the biosynthetic pathways for the formation of two GDP-3,6-dideoxy-heptoses. This pathway uses the 4,6-dehydratase to convert **5** into **6** and then the 3-dehydratase uses PLP and l-glutamate to make GDP-3,6-dideoxy-4-keto-α-d-*thre*o-heptose (**16**). In serotypes HS:5 and HS:11, the C5-epimerase inverts the stereochemistry at C5 to form GDP-3,6-dideoxy-4-keto-β-l-*xylo*-heptose (**17**). We have previously shown that the C4-reductases from HS:3 and HS:53 can reduce the 4-keto group of **16** to form the previously unknown compounds, GDP-3,6-dideoxy-α-d-*arabino*-heptose (**20**) and GDP-3,6-dideoxy-α-d-*lyxo*-heptose (**21**), respectively.^27^ These results demonstrate that the C4-reductases that appear in gene clusters for the biosynthesis of GDP-6-deoxy-heptoses can also function with GDP-4-keto-3,6-dideoxy substrates to generate 3,6-dideoxy heptoses that have not previously been identified within the CPS structures of *C. jejuni*.

### *In Vitro* Enzymatic Synthesis of Novel GDP-d-*glycero*-3-deoxy-heptoses

**Figure 12:**
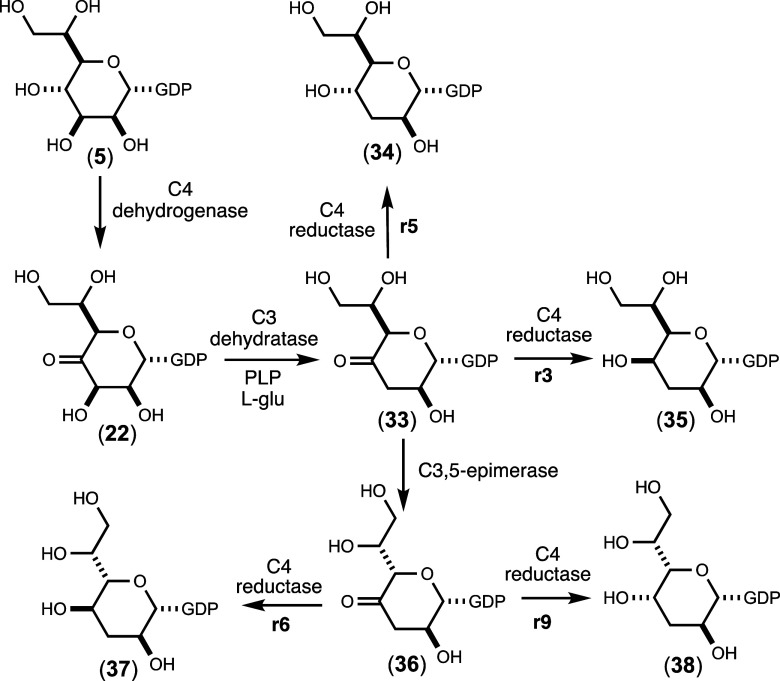
Pathway for the construction of four variants of GDP-d-*glycero*-3-deoxy-heptose with differences in stereochemistry at C4 and C5.

If some of the C4-reductases from *C. jejuni* can function with 3-deoxy substrates, then it is possible to envision the synthesis of GDP-d-*glycero*-3-deoxy-heptoses according to the scheme presented in Figure 12. Toward this end, we used the C4-dehydrogenase (Cj1427 from HS:2) with compound **5** to make **22** and then used the C3-dehydratase from HS:5 to make GDP-d-*glyero*-3-deoxy-4-keto-α-d-*threo*-heptose (**33**). The compound was purified using HPLC with the PA1 column, and the composition was confirmed by ESI-MS where the isolated material had a *m*/z of 616.08, compared with a calculated value of 616.08. In the absence of an added epimerase, the C4-reductase from serotype HS:53 was used to reduce the C4-keto group to generate GDP-d-*glycero*-3-deoxy-α-d-*lyx*o-heptose (**34**) and the C4-reductase from the HS:3 serotype to make GDP-d-*glycero*-3-deoxy-α-d-*arabino*-heptose (**35**). In the presence of a C3,5-epimerase and compound **33**, we made GDP-d-*glycero*-3-deoxy-4-keto-β-l-*erythro*-heptose (**36**), which was reduced by the C4-reductase from HS:2 to make GDP-d-*glyero*-3-deoxy-β-l-*ribo*-heptose (**37**) and by the reductase from HS:11 to make GDP-d-*glyero*-3-deoxy-β-l-*xylo*-heptose (**38**). The ^1^H NMR and ^1^H–^1^H COSY NMR spectra are presented in Figures S37–S40, respectively. For all four compounds, the *m*/*z* for the M-1 anion was found to be 618.09, compared with a calculated value of 618.08.

## Conclusions

The exterior surface of the human pathogen *C. jejuni* is coated with a capsular polysaccharide that helps it to evade the host immune system. Different strains and serotypes of *C. jejuni* have different repeating sequences of 2–5 carbohydrates. Some of the most common monosaccharides found in the CPS of *C. jejuni* are various flavors of heptoses. These include 6-deoxy-heptoses, 3,6-dideoxy-heptoses, 6-hydroxy-heptoses, and heptofuranosides with variations in stereochemistry at C3, C4, and C5. From the most common serotypes of *C. jejuni* identified to date, we and others have characterized 20 different enzymes that are utilized to make 14 different GDP-activated heptoses. It is highly likely that the modified heptoses all originate from GDP-d-*glycero*-α-d-*manno*-heptose (**5**). Formation of the 6-deoxy-heptoses proceeds from the 4,6-dehydration of **5** to generate the 4-keto-activated intermediate **6**, which renders C3 and C5 susceptible to racemization by either a C3 or C3/C5-epimerase followed by reduction of 4-keto group using a substrate-specific C4-reductase that results in the formation of any of 6 different 6-deoxy-heptoses. Formation of the 3,6-deoxy-heptoses is enabled by a PLP-dependent 3-dehydratase that eliminates the hydroxyl group at C3 from the GDP-6-deoxy-4-keto-*lyxo*-heptose (**6**) intermediate. Racemization at C5 by a C5-epimerase followed by reduction with a C4-reductase generates two of the four possible GDP-3,6-dideoxy-heptoses. GDP-d-*glycero*-heptose products are initiated by oxidation of **5** by a C4-dehydrogenase that is dependent on the recycling of NADH with α-ketoglutarate. The activated intermediate is subjected to racemization at C3 or C5 by the same two epimerases that have been shown to epimerize intermediate **6**. Again, different GDP-d-*glycero*-heptoses are produced by the substrate-specific C4-reductases used to reduce the corresponding 6-deoxy intermediates. However, it is still not clear how the l-*glycero*-d-*ido*-heptose is synthesized for the CPS from the HS:3 and HS:4 serotypes. The 20 enzymes that have been identified within the gene clusters for CPS formation in *C. jejuni* can be differentially combined to create novel GDP-activated heptoses. The largest remaining unsolved problem is the identification of the appropriate glycosyl transferase contained within the gene clusters for capsular polysaccharide formation for the elongation of the growing polysaccharide chain. Unusual polysaccharides are made, and it will be of significant interest to decipher the specificity requirements for the identification of the specific NTP-activated sugar and the appropriate sugar acceptor.

## Supporting Information Available

Sequence similarity matrices for enzymes required for heptose biosynthesis is *C. jejuni*; gene clusters for heptose biosynthesis from various serotypes of *C. jejuni*; and NMR spectra of reaction products (PDF)

## Accession Codes

C4-dehydrogenase (UniProt id: Q0P8I7) from serotype HS:2; C3-dehydratase (UniProt id: A0A0U3ALB0) from HS:5; epimerases and associated C4-reductases from serotypes HS:2 (UniProt id: Q0P8I4 and Q0P8I6), HS:3 (UniProt id: F2 × 702 and F2 × 701), HS:23/36 (UniProt id: Q6EF58 and Q5M6Q6), HS:33 (UniProt id: A0A0S2CGT2), HS:11 (UniProt id: A0A0U2QGV6 and A0A0U3ANW2), HS:15 (UniProt ids: A0A3Z9HSX9 and F2 × 7A7), HS:42 (UniProt ids: F2 × 7E5 and F2 × 7E4), and HS:51/HS:53 (UniProt id: Q5HSZ2).
