# Supplementary material for: Metabolic Pathways for the Biosynthesis of Heptoses Used in the Construction of Capsular Polysaccharides in the Human Pathogen Campylobacter jejuni
Source: Biochemistry. 2023 Oct 27;62(21):3145–58. doi: 10.1021/acs.biochem.3c00390 (PMC10680097; doi:10.1021/acs.biochem.3c00390)
Supplement: Supplementary file 1 — bi3c00390_si_001.pdf [file bi3c00390_si_001.pdf]

SUPPORTING INFORMATION

Metabolic Pathways for the Biosynthesis of Heptoses Used in the Construction of Capsular Polysaccharides in the Human Pathogen *Campylobacter jejuni*

Dao Feng Xiang, Maggie Xu, Manas K. Ghosh, and Frank M. Raushel*

Department of Chemistry, Texas A&M University, College Station, Texas, USA 77843

*To whom correspondence may be sent: raushel@tamu.edu

Table S1: Sequence identity matrix for 23 D-sedoheptulose-7-P isomerases from *C. jejuni.*

%ID (UniProt ID)	HS: 2	HS: 3	HS: 4	HS: 5	HS: 8	HS: 10	HS: 11	HS: 12	HS: 15	HS: 18	HS: 23	HS: 27	HS: 29	HS: 32	HS: 33	HS: 40	HS: 41	HS: 42	HS: 45	HS: 51	HS: 52	HS: 60	HS: 63
HS:2 (Q0P8J0/Q9PMN3)	100	82	96	98	97	96	97	99	97	97	98	95	95	97	97	96	96	99	98	98	97	93	93
HS:3 (F2X6Z6)		100	82	84	81	81	83	82	84	81	84	82	81	83	82	84	82	82	82	84	82	81	78
HS:4 (F2X742)			100	97	95	96	96	96	95	96	97	96	96	97	96	95	95	96	96	97	96	95	92
HS:5 (Q5M6R0)				100	98	97	98	99	97	98	100	98	96	98	99	97	97	99	97	100	98	95	93
HS:8 (F2X758)					100	97	98	98	97	99	98	97	95	96	96	95	97	98	97	98	98	92	95
HS:10 (F2X779)						100	98	96	95	97	97	97	96	98	95	95	97	96	96	97	98	93	93
HS:11 (A0A0U3C280)							100	97	96	98	98	98	96	97	97	96	98	97	96	98	99	94	93
HS:12 (F2X7E1)								100	98	98	99	96	95	97	98	96	96	100	99	99	97	93	94
HS:15 (F2X7A3)									100	97	97	95	95	97	96	95	95	98	98	97	96	93	93
HS:18 (A0A0S2UNQ1)										100	98	97	95	96	96	95	97	98	97	98	98	92	95
HS:23 (Q5M6R0)											100	98	96	98	99	97	97	99	97	100	98	95	93
HS:27 (A0A0S2CG71)												100	96	97	96	95	97	96	96	98	97	93	95
HS:29 (A0A0U3AB71)													100	97	95	94	95	95	95	96	96	94	91
HS:32 (A0A0S2CFX0)														100	97	96	96	97	98	98	98	95	93
HS:33 (A0A0S2CG62)															100	97	96	98	97	99	96	94	92
HS:40 (A0A0S2CGW3)																100	96	96	95	97	96	97	92
HS41 (Q5M6U3)																	100	96	95	97	99	93	93
HS:42 (F2X7E1)																		100	99	99	97	93	94
HS:45 (A0A0S2CFS5)																			100	97	96	94	93
HS:51 (Q5HSZ5)																				100	98	95	93
HS:52 (A0A0S2CGQ9)																					100	93	93
HS:60 (A0A0S2CFH2)																						100	91
HS:63 (A0A0S2CG85)																							100

Table S2: Sequence identity matrix for 23 D-*glycero*-D-*manno*-heptose 7-P kinases from *C. jejuni.*

% identity (UniProt id)	HS: 2	HS: 3	HS: 4	HS: 5	HS: 8	HS: 10	HS: 11	HS: 12	HS: 15	HS: 18	HS: 23	HS: 27	HS: 29	HS: 32	HS: 33	HS: 40	HS: 41	HS: 42	HS: 45	HS: 51	HS: 52	HS: 60	HS: 63
HS:2 (Q0P8I9)	100	97	97	97	97	96	96	97	96	96	97	96	98	97	97	94	95	97	97	97	96	96	96
HS:3 (F2X6Z8)		100	98	98	99	98	98	97	97	97	98	98	98	98	100	95	96	97	98	99	97	96	97
HS:4 (F2X743)			100	98	98	97	97	98	98	97	98	96	98	99	98	95	97	98	99	98	97	98	98
HS:5 (A0A0Q3QYE2)				100	98	97	97	97	96	97	99	96	98	98	98	97	98	97	98	98	96	96	97
HS:8 (F2X759)					100	98	96	97	96	97	98	97	98	98	99	96	96	97	98	97	96	96	97
HS:10 (F2X780)						100	96	97	96	97	98	96	98	98	98	95	96	97	98	97	97	96	97
HS:11 (A0A0U3C7E4)							100	96	96	96	96	98	97	97	98	93	95	96	97	99	97	95	96
HS:12 (F2X7E2)								100	98	96	97	96	97	98	97	96	96	100	98	97	97	97	97
HS:15 (F2X7A4)									100	96	96	95	96	97	97	95	96	98	97	97	96	96	96
HS:18 (A0A0S2UNM3)										100	97	95	97	98	97	94	96	96	98	97	96	96	97
HS:23 (Q6EF86)											100	97	98	98	98	97	98	97	98	97	96	96	97
HS:27 (A0A0S2CH33)												100	97	96	98	94	95	96	96	98	96	95	96
HS:29 (A0A0U3CEQ2)													100	99	98	95	97	97	99	98	97	97	97
HS:32 (A0A0S2CGR1)														100	98	95	96	98	100	98	97	98	97
HS:33 (F2X6Z8)															100	95	96	97	98	99	97	96	97
HS:40 (A0A0S2CFX2)																100	96	96	95	95	95	96	96
HS41 (Q5M6U1)																	100	96	96	96	95	96	97
HS:42 (F2X7E2)																		100	98	97	97	97	97
HS:45 (A0A0S2CGR1)																			100	98	97	98	97
HS:51 (Q5HSZ4)																				100	98	96	97
HS:52 (A0A0S2CFP2)																					100	96	97
HS:60 (A0A0S2CGI5)																						100	96
HS:63 (A0A0S2CGB1)																							100

Table S3: Sequence identity matrix for 23 D-*glycero*-α-D-*manno*-heptose-1-P guanylyltransferases from *C. jejuni.*

% identity (UniProt ID)	HS: 2	HS: 3	HS: 4	HS: 5	HS: 8	HS: 10	HS: 11	HS: 12	HS: 15	HS: 18	HS: 23	HS: 27	HS: 29	HS: 32	HS: 33	HS: 40	HS: 41	HS: 42	HS: 45	HS: 51	HS: 52	HS: 60	HS: 63
HS:2 (Q0P8J1)	100	93	92	91	91	87	89	88	91	89	89	92	91	91	89	91	86	88	97	85	91	91	91
HS:3 (F2X6Z5)		100	90	90	90	87	88	90	90	87	89	89	91	91	92	90	88	90	94	85	89	91	87
HS:4 (F2X741)			100	92	89	85	87	88	92	87	88	88	90	90	87	89	86	88	91	84	90	92	88
HS:5 (A0A0Q3MD31)				100	93	88	88	90	99	89	94	88	94	92	86	92	88	90	92	83	88	94	91
HS:8 (F2X757)					100	90	91	94	93	91	96	90	96	94	86	93	91	94	91	84	90	93	93
HS:10 (F2X778)						100	91	89	87	90	89	86	93	88	87	88	91	89	88	82	88	89	91
HS:11 (A0A0U3AL78)							100	91	88	90	90	87	91	91	87	89	92	91	88	83	87	90	91
HS:12 (F2X7C2)								100	90	89	91	86	92	94	86	91	91	100	89	83	88	92	89
HS:15 (F2X7A2)									100	90	94	88	94	92	86	92	88	90	92	83	88	94	91
HS:18 (A0A0S2UNK0)										100	93	93	93	89	85	88	90	89	87	85	94	90	92
HS:23 (Q5M6R1)											100	90	96	92	85	92	91	91	90	84	91	93	92
HS:27 (A0A0S2CG77)												100	92	89	86	87	89	86	90	85	93	87	89
HS:29 (A0A0U3A9Z0)													100	94	86	93	92	92	92	84	91	95	93
HS:32 (A0A0S2CFE5)														100	86	93	91	94	91	82	88	92	89
HS:33 (A0A0S2CH11)															100	86	87	86	88	85	87	87	86
HS:40 (A0A0S2CG02)																100	90	91	92	82	88	92	90
HS41 (Q5M6U4)																	100	91	87	83	89	90	91
HS:42 (F2X7C2)																		100	89	83	88	92	89
HS:45 (A0A0S2CFT5)																			100	84	89	91	89
HS:51 (Q5HSZ6)																				100	87	85	83
HS:52 (A0A0S2CFN6)																					100	88	90
HS:60 (A0A0S2CFD2)																						100	91
HS:63 (A0A0S2CH69)																							100

Table S4: Sequence identity matrix for 19 GDP-D-*glycero*-α-D-*manno*-heptose 4,6-dehydratases from *C. jejuni.*

% Identity (UnitProt id)	HS: 3	HS: 4	HS: 5	HS: 8	HS: 10	HS: 11	HS: 12	HS: 15	HS: 18	HS: 23	HS: 29	HS: 32	HS: 41	HS: 42	HS; 45	HS: 51	HS: 52	HS: 60	HS: 63
HS:3 (F2X700)	100	98	89	93	93	90	93	92	91	98	90	92	93	93	92	91	93	94	97
HS:4 (F2X745)		100	89	92	92	89	92	91	90	98	91	91	92	91	92	90	92	94	97
HS:5 (A0A0U2JTD3)			100	92	92	94	93	91	92	89	93	92	92	93	92	93	92	93	90
HS:8 (F2X760)				100	97	93	96	95	95	92	94	95	97	96	95	96	96	96	91
HS:10 (F2X781)					100	93	95	94	94	92	93	95	96	95	95	94	96	97	91
HS:11 (A0A0U2SRK8)						100	94	92	92	89	91	93	93	93	93	94	94	94	89
HS:12 (A0A0U3BGH1)							100	95	94	92	93	94	96	99	94	95	97	97	92
HS:15 (F2X7A5)								100	94	91	94	94	96	95	94	94	95	95	91
HS:18 (A0A0S2UNQ0)									100	90	94	94	94	93	94	95	94	95	90
HS:23 (Q5M6Q7)										100	90	90	92	91	90	90	92	93	97
HS:29 (A0A0U2JTC1)											100	93	94	93	94	95	94	95	90
HS:32 (A0A0S2CFG4)												100	96	94	97	95	96	96	91
HS:41 (Q5M6U0)													100	97	95	94	98	97	92
HS:42 (F2X7E3)														100	94	94	96	97	91
HS:45 (A0A0S2CFT2)															100	96	95	97	90
HS:51 (Q5HSZ3)																100	94	95	89
HS:52 (A0A0S2CGA9)																	100	97	92
HS:60 (A0A0S2CFH3)																		100	93
HS:63 (A0A0S2CHE1)																			100

Table S5: Sequence identity matrix for 18 GDP-4-keto-α-D-*lyxo*-heptose epimerases from *C. jejuni*. Those shaded blue are C5-epimerases, those shaded green are C3-epimerases, and those shaded tan are C3/C5-epimerases.

% Identity (UniProt id)	HS:11	HS:5	HS:45	HS:23	HS:41	HS:10	HS:29	HS:8	HS:33	HS:3	HS:4	HS:12	HS:52	HS:63	HS:2	HS:42	HS:15	HS:32
HS:11 (A0A0U2QGV6)	100	88	89	71	72	71	71	73	71	73	73	72	73	59	73	69	69	68
HS:5 (A0A0Q3UFL0)		100	97	73	72	73	73	73	73	75	74	73	73	63	77	72	72	71
HS:45 (A0A0S2CGS4)			100	72	72	72	72	73	72	74	73	72	73	63	76	71	71	70
HS:23 (Q6EF58)				100	92	94	93	94	92	91	91	90	91	67	81	76	76	75
HS:41 (Q5M6T7)					100	97	95	91	93	94	94	94	95	68	80	78	78	77
HS:10 (F2X784)						100	97	91	94	94	94	96	96	68	79	80	79	78
HS:29 (A0A0U3C7G1)							100	90	93	92	92	93	93	68	78	78	78	77
HS:8 (F2X764)								100	93	94	94	94	95	68	82	76	76	75
HS:33 (A0A0S2CG67)									100	94	94	96	96	68	79	78	78	77
HS:3 (F2X702)										100	99	96	97	69	80	78	78	77
HS:4 (F2X747)											100	96	97	69	80	78	78	77
HS:12 (A0A0U3C3H8)												100	99	69	79	78	78	77
HS:52 (A0A0S2CFK3)													100	68	80	79	79	78
HS:63 (A0A0S2CG98)														100	85	80	81	80
HS:2 (Q0P8I4)															100	88	89	88
HS:42 (F2X7E5)																100	98	97
HS:15 (F2X7A7)																	100	99
HS:32 (A0A0S2CFK0)																		100

Table S6: Sequence identity matrix for 25 C4 reductases from *C. jejuni* sorted by similarity. Additional details are found in the primary text.

%ID	HS: 11	HS: 18	HS: 51	HS: 33	HS: 29A	HS: 4	HS: 3	HS: 8B	HS: 5	HS: 45	HS: 2	HS: 63A	HS: 15	HS: 32	HS: 29B	HS: 63B	HS: 10B	HS: 41B	HS: 42	HS: 52	HS: 12	HS: 23	HS: 8A	HS: 10A	HS: 41A
HS:11 (A0A0U3ANW2)	100	47	51	50	46	45	47	47	44	43	46	46	54	54	53	52	52	51	52	48	48	48	47	46	47
HS:18 (A0A0S2UNM9)		100	97	48	45	43	44	44	44	44	47	48	45	44	50	50	50	51	50	51	52	51	52	52	53
HS:51 (Q5HSZ2)			100	50	48	46	47	47	45	45	47	48	50	50	54	54	54	54	54	54	55	54	54	55	55
HS:33 (A0A0S2CGT2)				100	59	58	58	58	57	57	59	60	50	50	56	55	56	55	55	59	59	59	59	59	58
HS:29A (A0A0U3C2A7)					100	92	94	93	56	55	60	62	46	46	51	51	51	51	51	53	52	53	53	52	53
HS:4 (F2X746)						100	97	96	55	55	59	59	49	49	53	54	53	54	53	54	54	55	52	50	50
HS:3 (F2X701)							100	98	56	55	60	60	49	49	53	53	53	53	53	53	53	55	51	50	51
HS:8B (F2X761)								100	57	55	60	60	49	49	53	53	53	54	53	53	53	54	51	50	50
HS:5 (A0A0U3C2F2)									100	96	77	76	46	45	55	54	55	54	54	53	54	55	53	52	52
HS:45 (A0A0S2CFV4)										100	76	75	45	44	55	53	55	53	54	53	54	55	53	52	52
HS:2 (Q0P8I6)											100	96	46	45	56	54	56	55	54	56	57	57	56	56	56
HS:63A (A0A0S2CGZ7)												100	46	46	56	54	56	55	55	58	57	57	58	59	59
HS:15 (F2X7A6)													100	99	57	58	56	57	57	53	53	53	51	50	50
HS:32 (A0A0S2CFG6)														100	57	58	56	57	57	53	53	53	51	50	50
HS:29B (A0A0U3AL93)															100	95	96	96	95	63	64	63	62	60	60
HS:63B (A0A0S2CGF0)																100	95	97	97	64	64	64	62	61	61
HS:10B (F2X782)																	100	97	96	64	64	64	62	61	61
HS:41B (Q5M6T9)																		100	97	63	64	63	62	60	60
HS:42 (F2X7E4)																			100	63	63	63	62	60	60
HS:52 (A0A0S2CFL3)																				100	98	97	97	95	95
HS:12 (A0A0U3BZJ9)																					100	98	95	94	94
HS:23 (Q5M6Q6)																						100	95	93	93
HS:8A (F2X762)																							100	97	97
HS:10A (F2X783)																								100	99
HS:41A (Q5M6T8)																									100

Table S7: Sequence identity matrix for four GDP-6-deoxy-α-D-*manno*-heptose 3-dehydratases from *C. jejuni*.

% Identity (UniProt id)	HS:5	HS:11	HS:45	HS:60
HS:5 (A0A0U3ALB0)	100	95	94	95
HS:11 (A0A0U2RG51)		100	94	96
HS:45 (A0A0S2CGN8)			100	96
HS:60 (A0A0S2CG22)				100

Table S8: Sequence identity matrix for five GDP-D-*glycero*-α-D-*manno*-heptose C4-dehydrogenases from *C. jejuni* .

% Identity (UniProt id)	HS:2	HS:3	HS:4	HS:23	HS:33
HS:2 (Q0P8I7)	100	97	98	97	98
HS:3 (F2X722)		100	99	98	99
HS:4 (F2X744)			100	99	99
HS:23/36 (Q5M6Q8)				100	99
HS:33 (F2X6Z9)					100

Table S9: Sequence identity matrix for three pyranose/furanose mutases from *C. jejuni.*

% Identity (UniProt id)	HS:10	HS:41	HS:60
HS:10 (F2X785)	100	88	62
HS:41 (Q5M6T6)		100	62
HS:60 (A0A0S2CFE4)			100

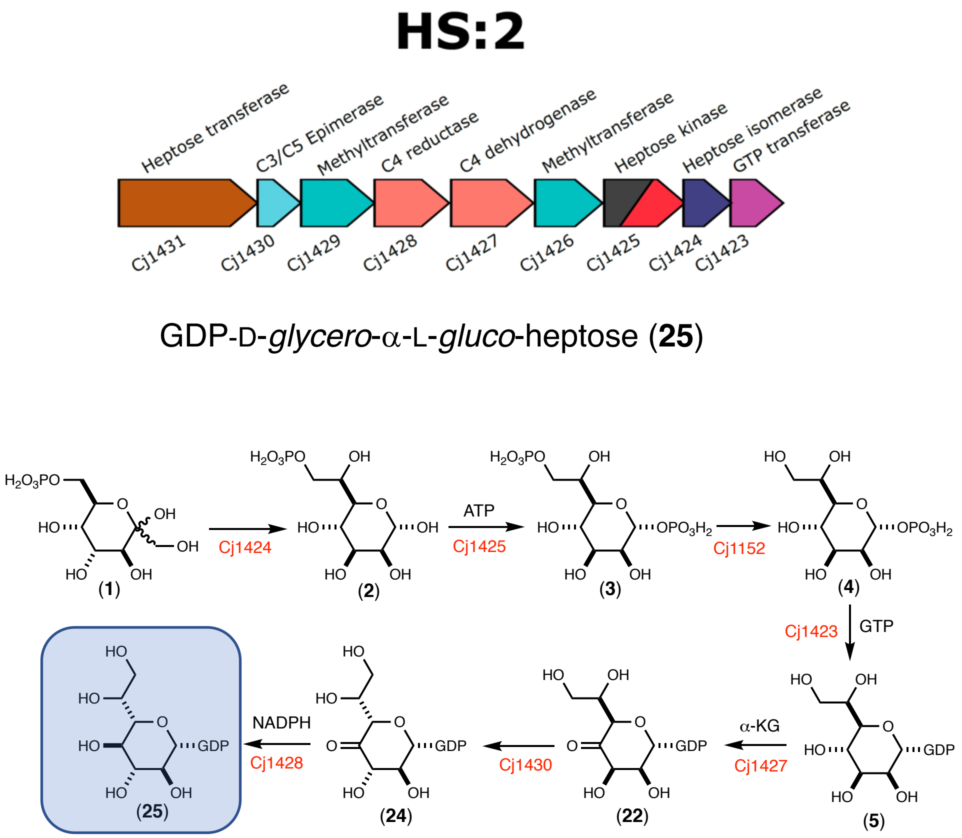

Notes

1. D-*glycero*-α-L-*gluco*-heptose has been identified in the CPS of serotype HS:2 (*1*).

Figure S1: Gene cluster for GDP-heptose formation in the HS:2 serotype of *C. jejuni* (GenBank Accession id: AL111168.1) and enzyme-catalyzed reactions for the formation of GDP-D-*glycero*-α-L-*gluco*-heptose (**25**) from D-sedoheptulose-7-phosphate (**1**).

HS:3

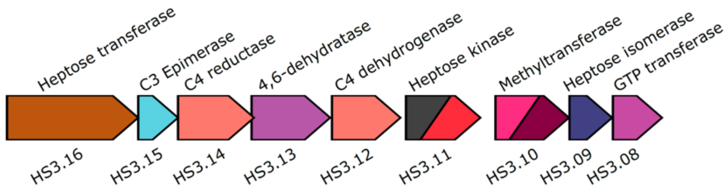

GDP-6-deoxy-α-D-*ido*-heptose (12)
GDP-D-*glycero*-α-D-*ido*-heptose (28)

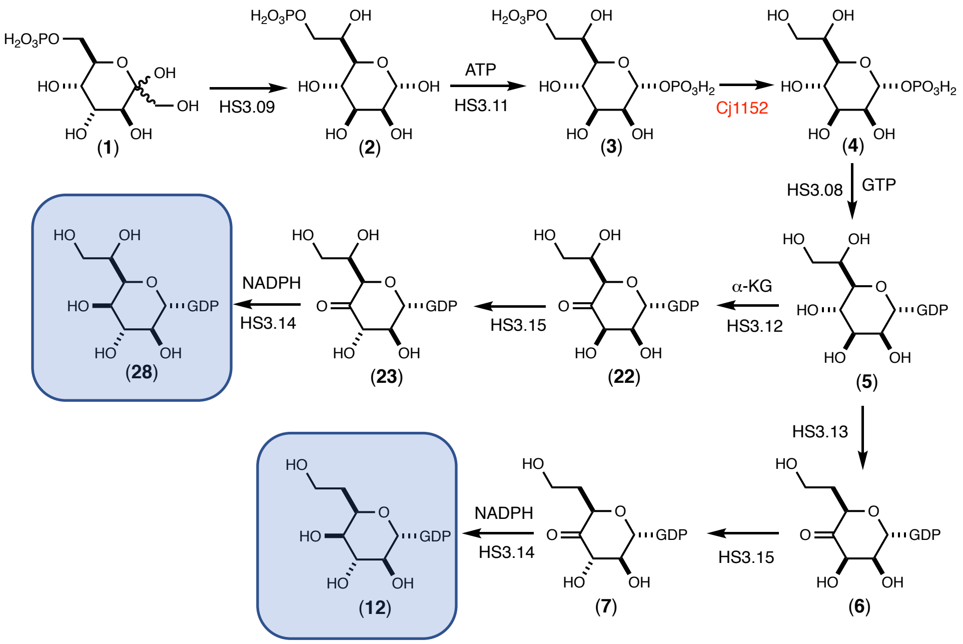

Notes

1. The reported heptoses in the HS:3 serotype of *C. jejuni* are L-*glycero*-α-D-*ido*-heptose and 6-deoxy-α-D-*ido*-heptose (*1*). It is not apparent how the relative stereochemistry at C6 of L-*glycero*-α-D-*ido*-heptose is inverted from that found in GDP-D-*glycero*-α-D-*manno*-heptose (**5**).

Figure S2: Gene cluster for GDP-heptose formation in the HS:3 serotype of *C. jejuni* (GenBank Accession id: HQ343268.1) and enzyme-catalyzed reactions for the formation of GDP-6-deoxy-α-D-*ido*-heptose (**12**) and GDP-D-*glycero*-α-D-*ido*-heptose (**2**8) from D-sedoheptulose-7-phosphate (**1**).

HS:4

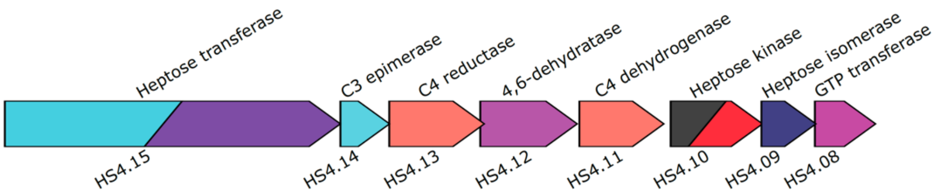

GDP-6-deoxy-α-D-*ido*-heptose (12)
GDP-D-*glycero*-α-D-*ido*-heptose (28)

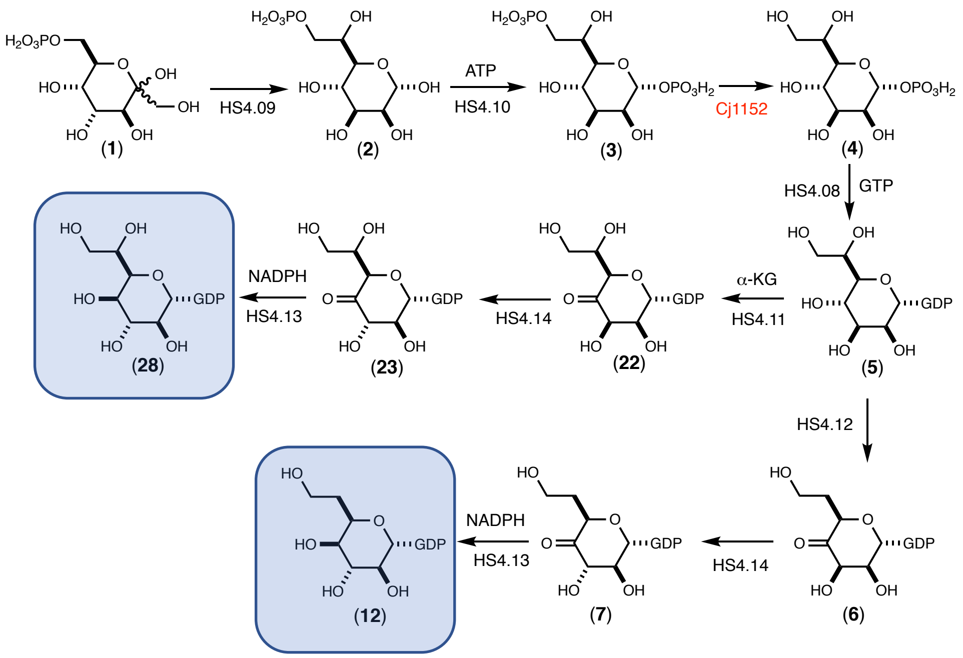

Notes

1. The reported heptoses in the HS:4 serotype of *C. jejuni* are L-*glycero*-α-D-*ido*-heptose and 6-deoxy-α-D-*ido*-heptose (*1*). It is not apparent how the relative stereochemistry at C6 of L-*glycero*-α-D-*ido*-heptose is inverted from that found in GDP-D-*glycero*-α-D-*manno*-heptose (**5**).

Figure S3: Gene cluster for GDP-heptose formation in the HS:3 serotype of *C. jejuni* (GenBank Accession id: HQ343269.1) and enzyme-catalyzed reactions for the formation of GDP-6-deoxy-α-D-*ido*-heptose (**12**) and GDP-D-*glycero*-α-D-*ido*-heptose (**28**) from D-sedoheptulose-7-phosphate (**1**).

HS:5

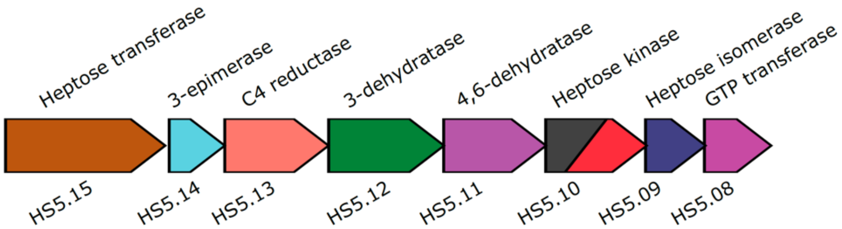

GDP-3,6-dideoxy-β-L-*ribo*-heptose (18)
GDP-D-*glycero*-α-D-*manno*-heptose (5)

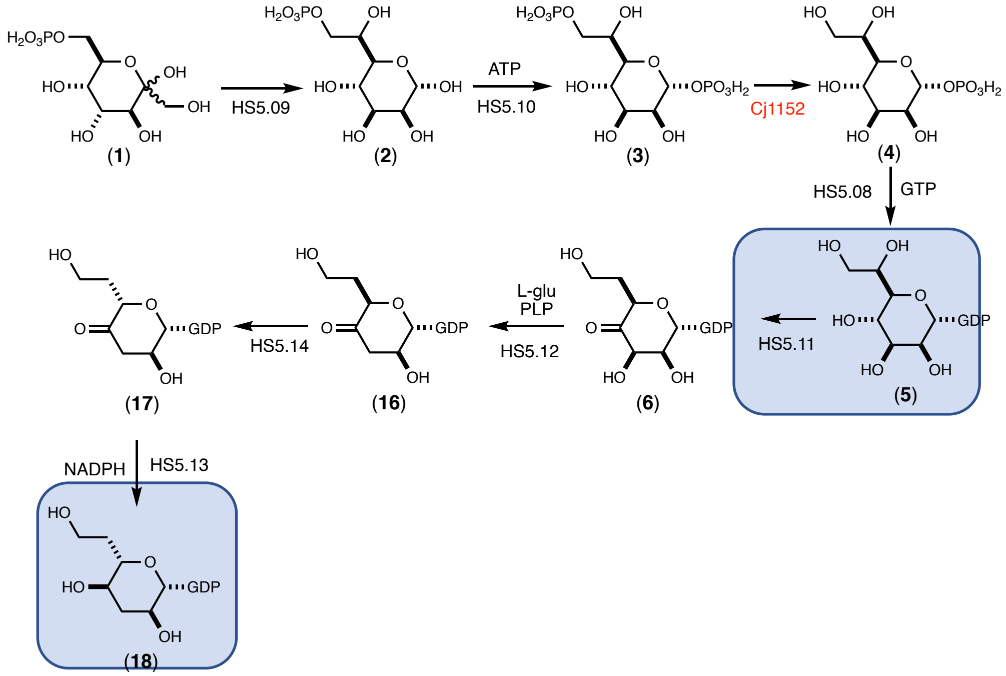

Notes

1. The reported heptoses in the HS:5 serotype of *C. jejuni* are D-*glycero*-α-D-*manno*-heptose and 3,6-dideoxy-α-D-*ribo*-heptose (*1*). It has been demonstrated that the enzymes from this gene cluster make GDP-3,6-dideoxy-β-L-*ribo*-heptose (**18**) (*2*).

Figure S4: Gene cluster for GDP-heptose formation in the HS:5 serotype of *C. jejuni* (GenBank Accession id: KT868843.1) and enzyme-catalyzed reactions for the formation of GDP-D-glycero-α-D-*manno*-heptose (**5**) and GDP-3,6-dideoxy-β-L-*ribo*-heptose (**18**) from D-sedoheptulose-7-phosphate (**1**).

HS:8

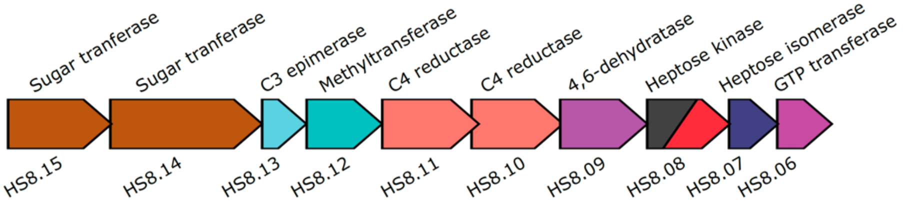

GDP-6-deoxy-α-D-*ido*-heptose (12)
GDP-6-deoxy-α-D-*altro*-heptose (11)

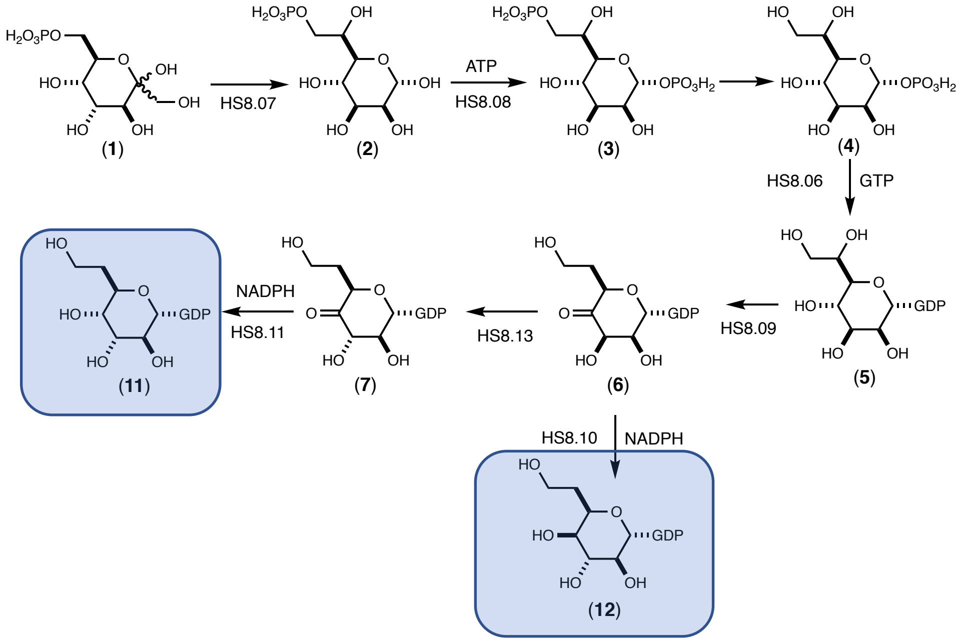

Notes

1. At the 3’-end of the gene for HS8.11 there is a poly-G tract that may influence whether HS8.11 or HS8.10 is expressed in a catalytically active form (*3*).

Figure S5: Gene cluster for GDP-heptose formation in the HS:5 serotype of *C. jejuni* (GenBank Accession id: HQ343270.1) and enzyme-catalyzed reactions for the formation of GDP-6-deoxy-α-D-*ido*-heptose (12) and GDP-6-deoxy-α-D-*altro*-heptose (**11**) from D-sedoheptulose-7-phosphate (**1**).

HS:10

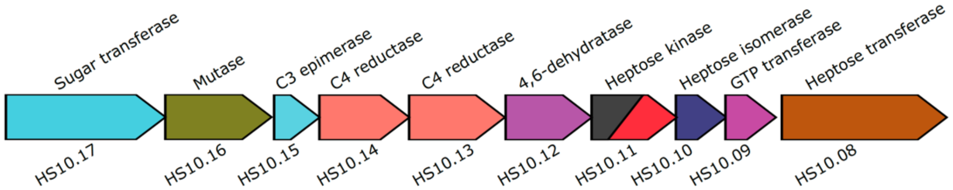

GDP-6-deoxy-β-L-*galacto*-heptofuranoside (32)
GDP-6-deoxy-α-D-*altro*-heptofuranoside (31)

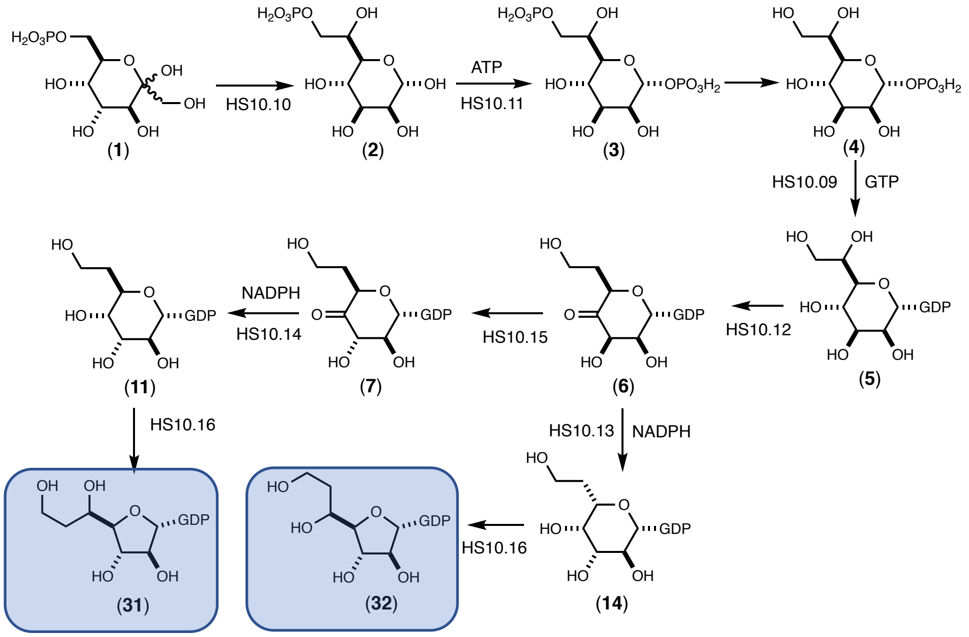

Notes

1. The reported heptose in the HS:10 serotype of *C. jejuni* is 6-deoxy-L-*galacto*-heptofuranose (*1*). At the 3'-end of the gene for HS10.14 there is a poly-G tract that may influence whether HS10.14 or HS10.13 is expressed in a catalytically active form (*3*).

Figure S6: Gene cluster for GDP-heptose formation in the HS:10 serotype of *C. jejuni* (GenBank Accession id: HQ343271.1) and enzyme-catalyzed reactions for the formation of GDP-6-deoxy-β-L-*galacto*-heptofuranoside (**32**) and GDP-6-deoxy-α-D-*altro*-heptofuranoside (**31**) from D-sedoheptulose-7-phosphate (**1**).

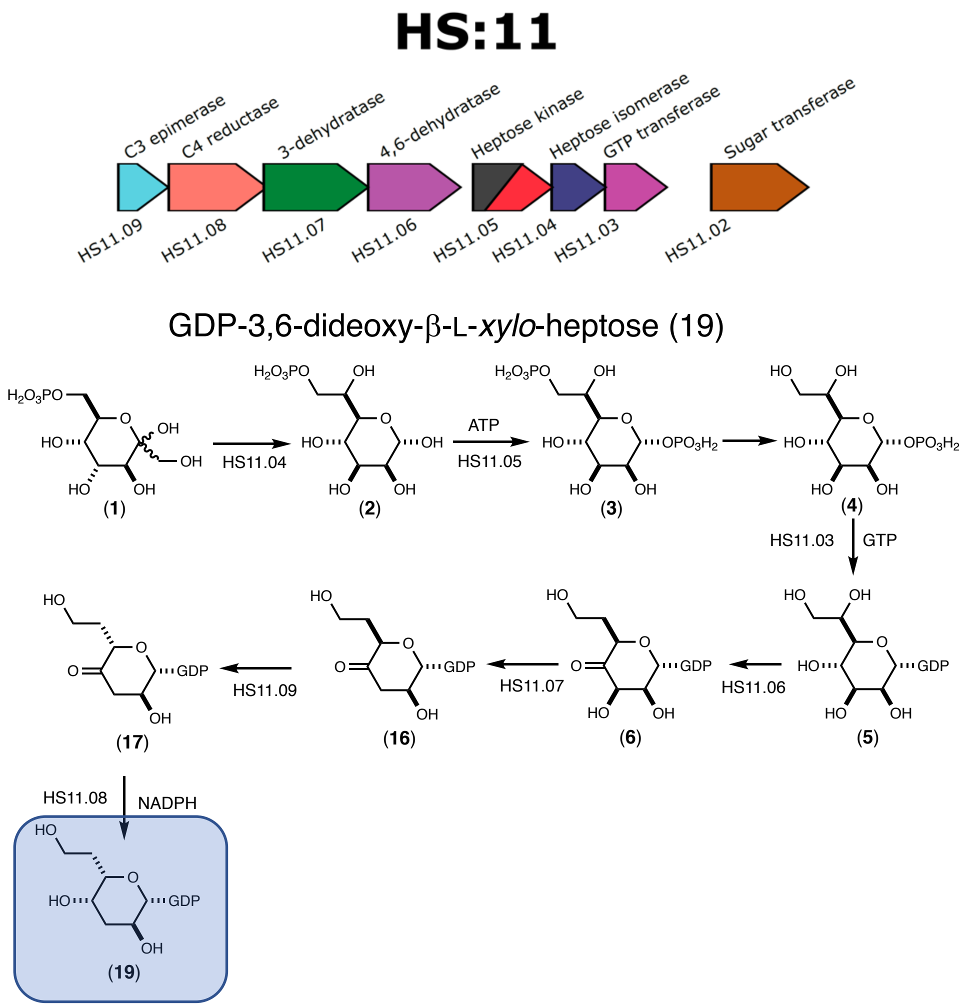

Figure S7: Gene cluster for GDP-heptose formation in the HS:11 serotype of *C. jejuni* (GenBank Accession id: KT868845.1) and enzyme-catalyzed reactions for the formation of GDP-3,6-dideoxy-β-L-*xylo*-heptose (**19**) from D-sedoheptulose-7-phosphate (**1**).

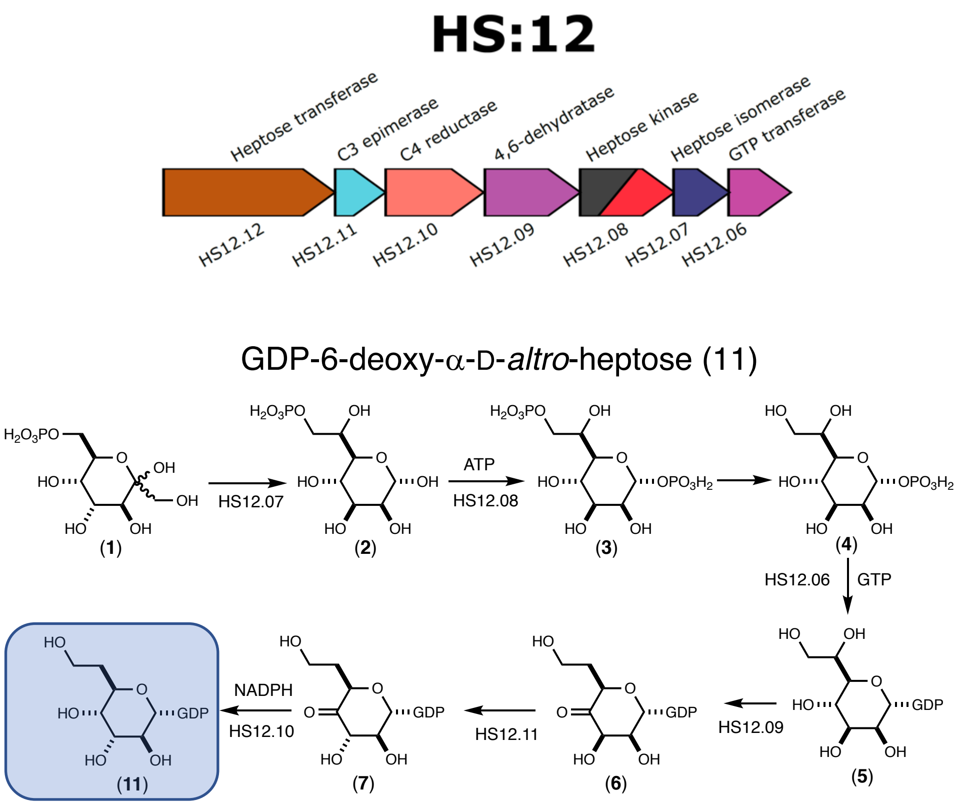

Figure S8: Gene cluster for GDP-heptose formation in the HS:12 serotype of *C. jejuni* (GenBank Accession id: KT868845.1) and enzyme-catalyzed reactions for the formation of GDP-6-deoxy-α-D-*altro*-heptose (**11**) from D-sedoheptulose-7-phosphate (**1**).

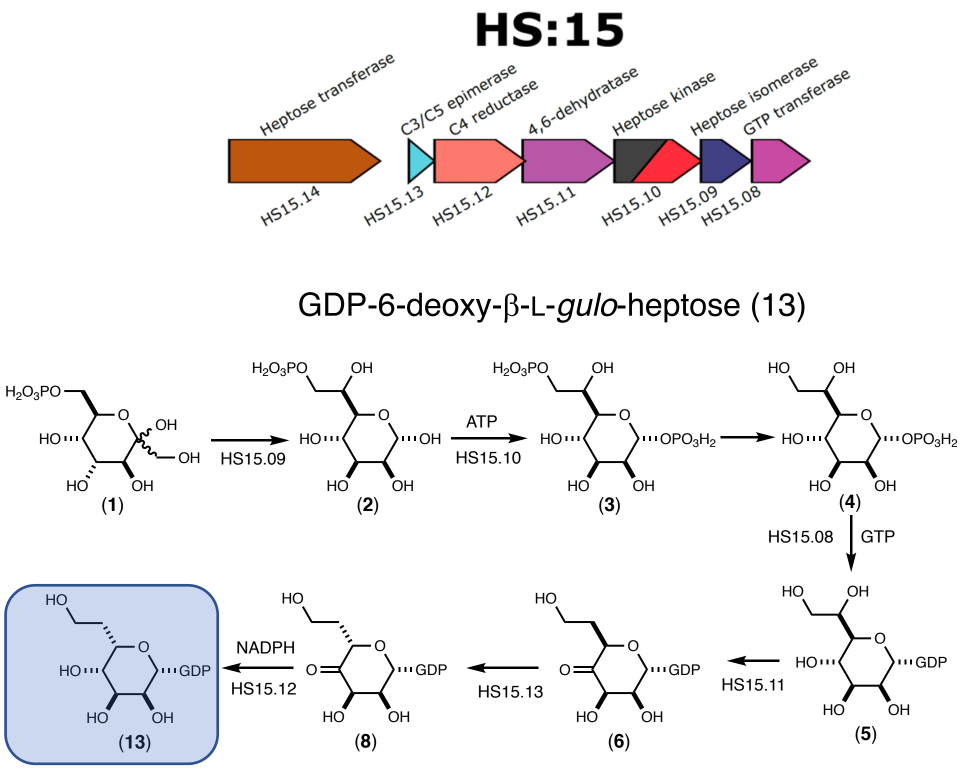

Notes

1. The reported heptose in the HS:15 serotype of *C. jejuni* is 6-deoxy-α-L-*gulo*-heptose (*1*).

Figure S9: Gene cluster for GDP-heptose formation in the HS:15 serotype of *C. jejuni* (GenBank Accession id: HQ343272.1) and enzyme-catalyzed reactions for the formation of GDP-6-deoxy-β-L-*gulo*-heptose (**13**) from D-sedoheptulose-7-phosphate (**1**).

HS:18

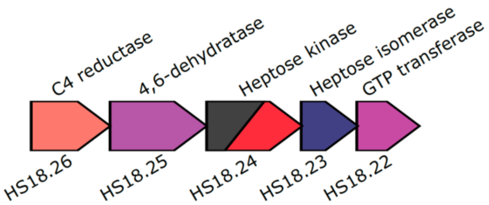

GDP-6-deoxy-α-D-*manno*-heptose (10)

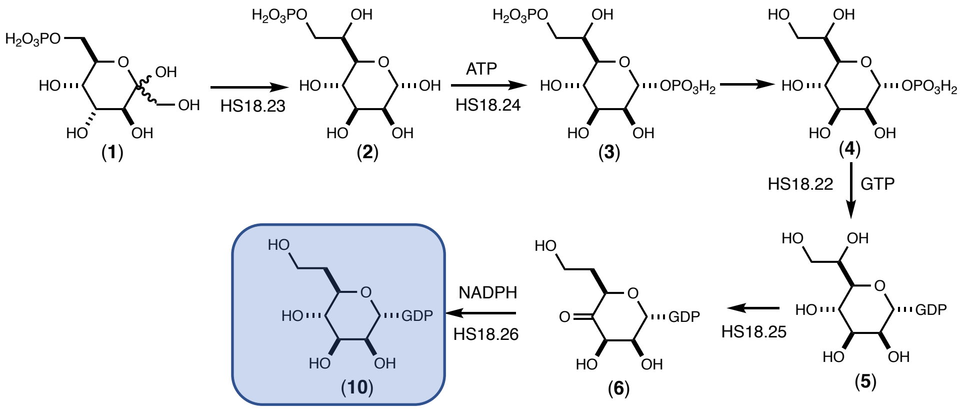

Figure S10: Gene cluster for GDP-heptose formation in the HS:12 serotype of *C. jejuni* (GenBank Accession id: KT932997.1) and enzyme-catalyzed reactions for the formation of GDP-6-deoxy-α-D-*manno*-heptose (**10**) from D-sedoheptulose-7-phosphate (**1**).

HS23/36

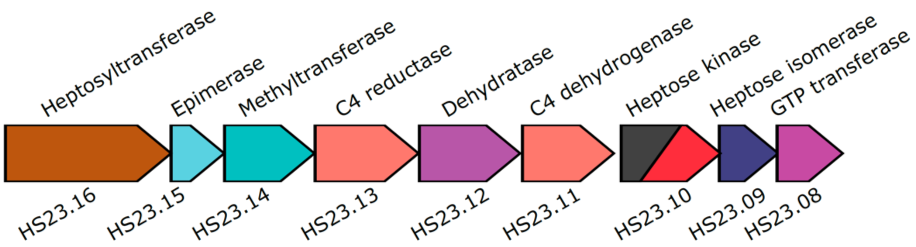

GDP-6-deoxy-α-D-*altro*-heptose (11)
GDP-D-*glycero*-α-D-*altro*-heptose (27)

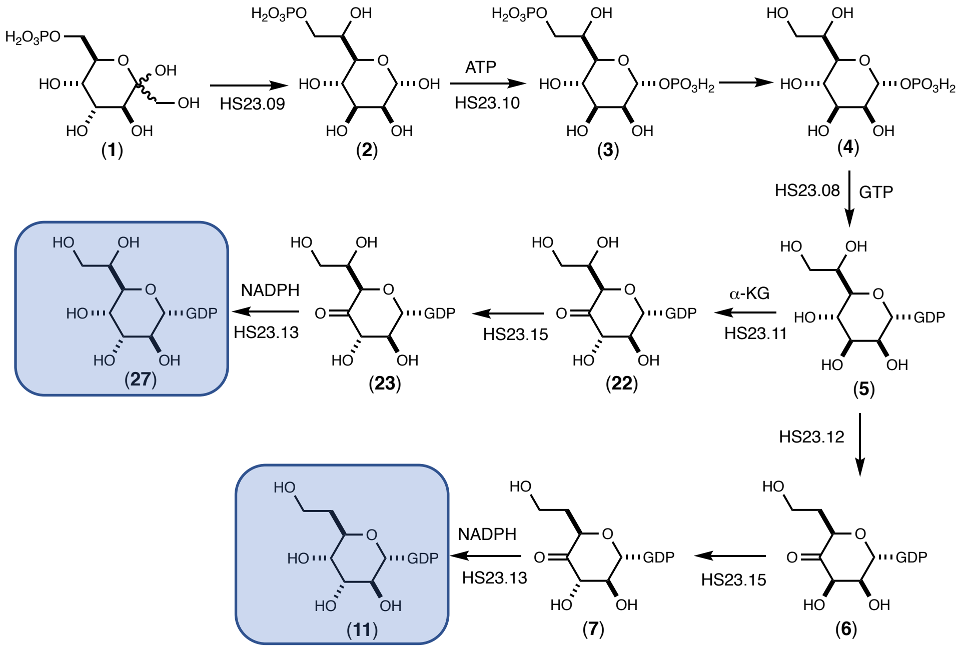

Notes

1. The reported heptoses in the HS:23/36 serotype of *C. jejuni* are 6-deoxy-α-D-*altro*-heptose and D-*glycero*-α-D-*altro*-heptose (*1*).

Figure S11: Gene cluster for GDP-heptose formation in the HS:15 serotype of *C. jejuni* (GenBank Accession id: HQ343272.1) and enzyme-catalyzed reactions for the formation of GDP-6-deoxy-β-L-*gulo*-heptose (**13**) from D-sedoheptulose-7-phosphate (**1**).

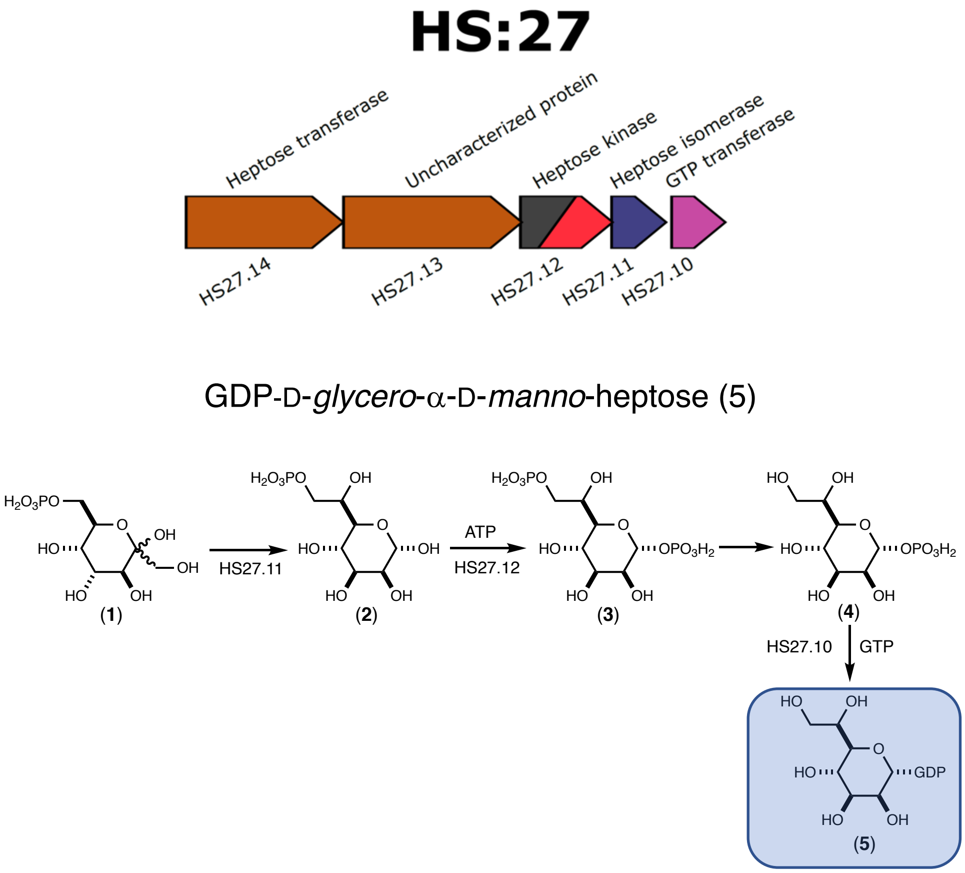

Figure S12: Gene cluster for GDP-heptose formation in the HS:27 serotype of *C. jejuni* (GenBank Accession id: KT893437.1) and enzyme-catalyzed reactions for the formation of GDP-D-*glycero*-α-D-*manno*-heptose (**5**) from D-sedoheptulose-7-phosphate (**1**).

HS:29

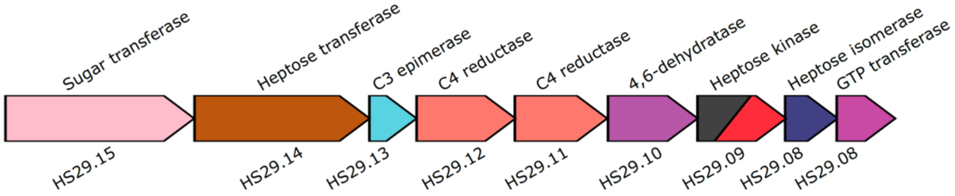

GDP-6-deoxy-α-D-*ido*-heptose (12)
GDP-6-deoxy-β-L-*galacto*-heptose (14)

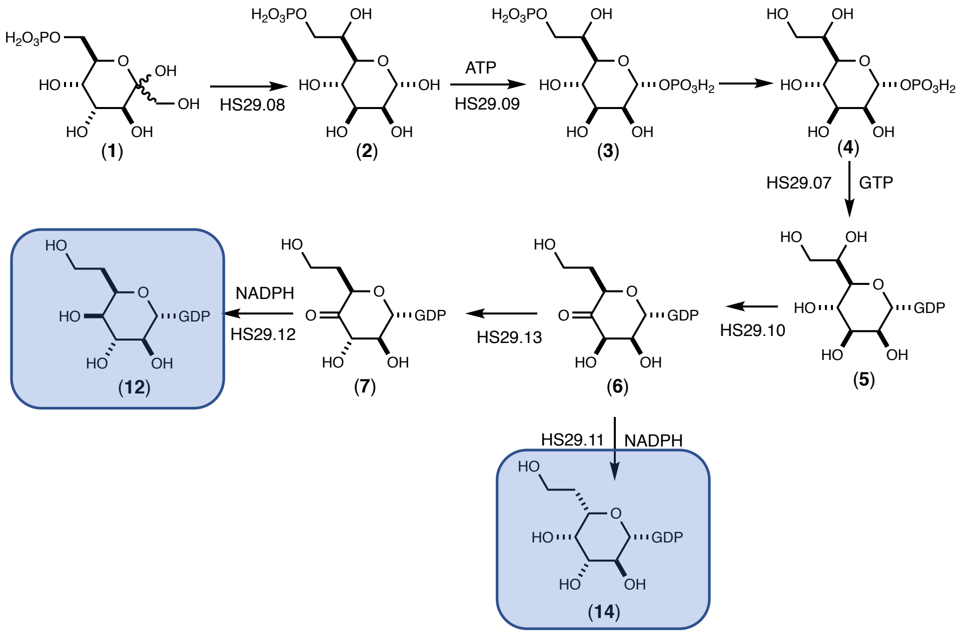

Notes

1. At the 3'-end of the gene for HS29.12 there is a poly-G tract that may influence whether HS29.12 or HS29.11 is expressed in a catalytically active form (*3*).

Figure S13: Gene cluster for GDP-heptose formation in the HS:29 serotype of *C. jejuni* (GenBank Accession id: KT868846.1) and enzyme-catalyzed reactions for the formation of GDP-6-deoxy-α-D-*ido*-heptose (**12**) and GDP-6-deoxy-β-L-*galacto*-heptose (**14**) from D-sedoheptulose-7-phosphate (**1**).

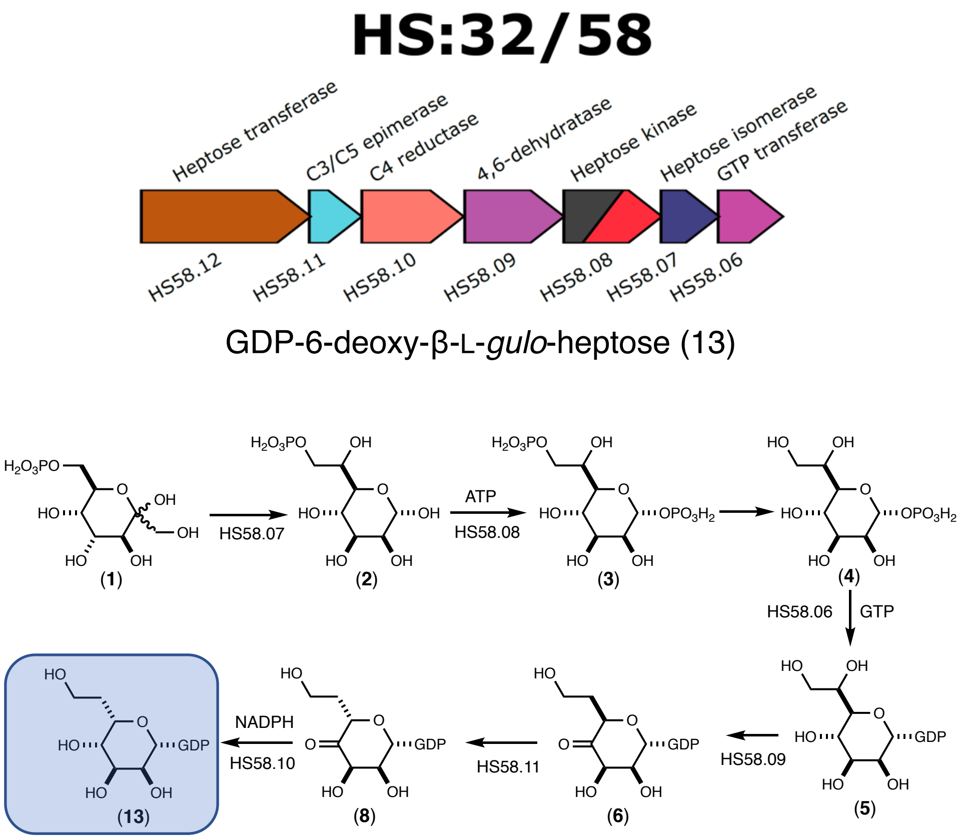

Figure S14: Gene cluster for GDP-heptose formation in the HS:58 serotype of *C. jejuni* (GenBank Accession id: KT893427.1) and enzyme-catalyzed reactions for the formation of GDP-6-deoxy-β-L-*gulo*-heptose (**13**) from D-sedoheptulose-7-phosphate (**1**).

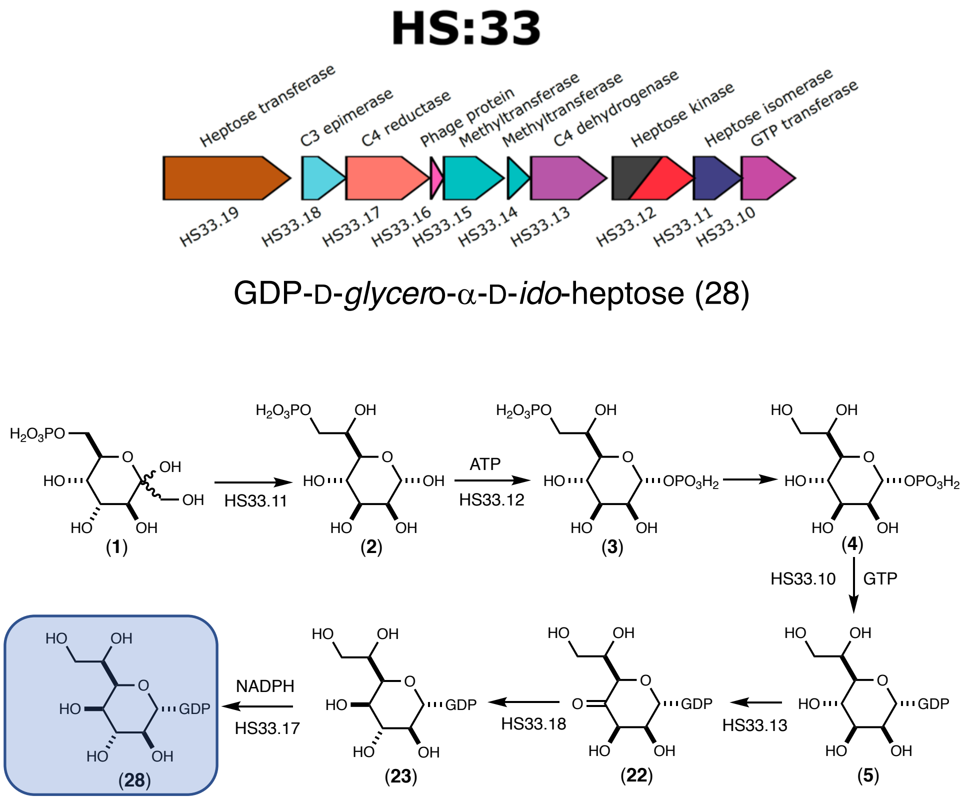

Figure S15: Gene cluster for GDP-heptose formation in the HS:33 serotype of *C. jejuni* (GenBank Accession id: KT893436.1) and enzyme-catalyzed reactions for the formation of GDP-D-*glycero*-α-D-*ido*-heptose (**28**) from D-sedoheptulose-7-phosphate (**1**).

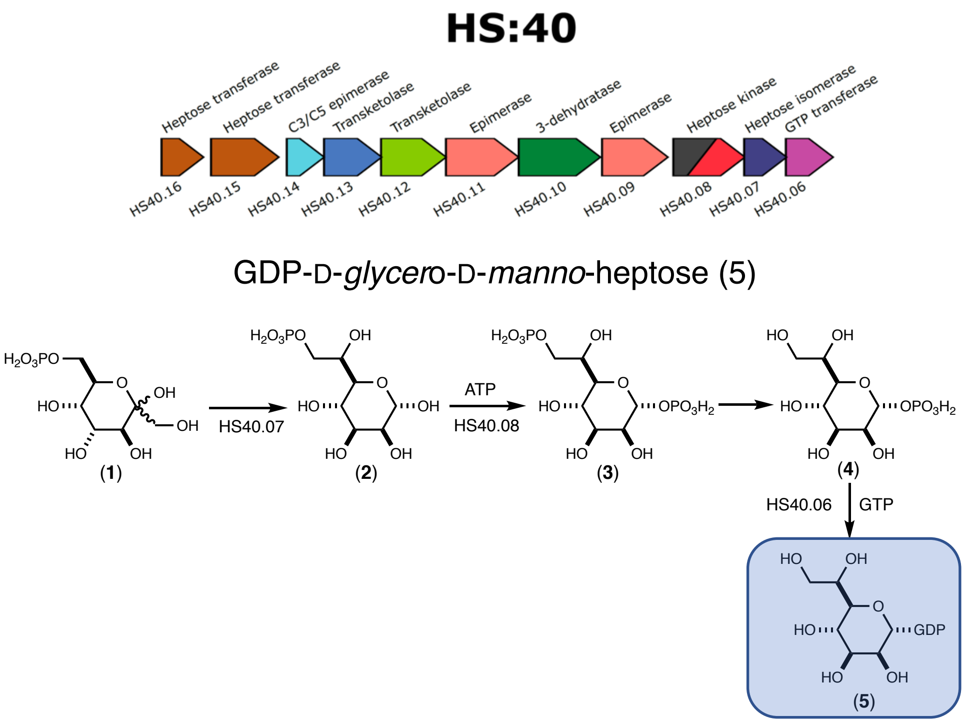

Figure S16: Gene cluster for GDP-heptose formation in the HS:40 serotype of *C. jejuni* (GenBank Accession id: KT893434.1) and enzyme-catalyzed reactions for the formation of GDP-D-*glycero*-α-D-*manno*-heptose (**5**) from D-sedoheptulose-7-phosphate (**1**).

HS:41

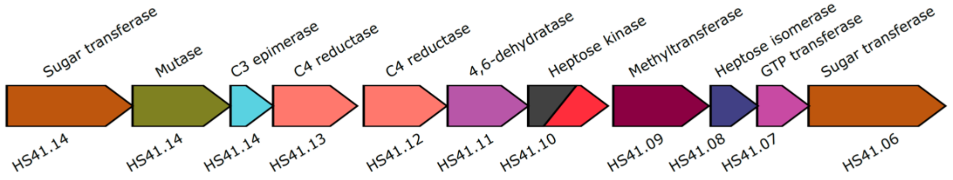

GDP-6-deoxy-β-L-*galacto*-heptofuranoside (32)
GDP-6-deoxy-α-D-*altro*-heptofuranoside (31)

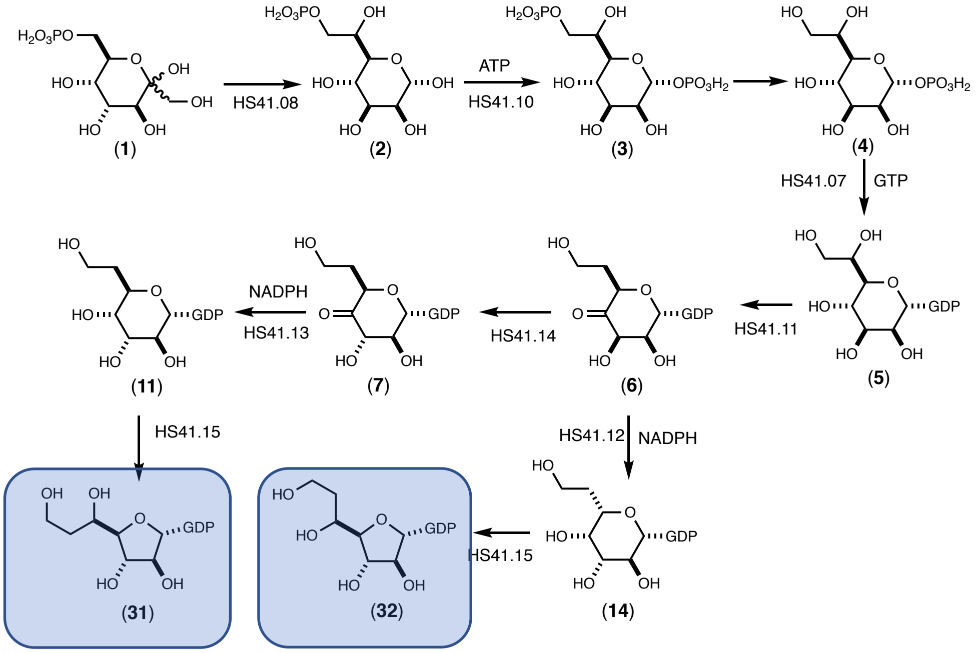

Notes

1. At the 3'-end of the gene for HS41.13 there is a poly-G tract that may influence whether HS41.13 or HS41.12 is expressed in a catalytically active form (*3*).
2. The CPS from serotype HS:41 was shown to contain 6-deoxy-β-D-*altro*-heptofuranoside (*1*).

Figure S17: Gene cluster for GDP-heptose formation in the HS:29 serotype of *C. jejuni* (GenBank Accession id: BX545857.1) and enzyme-catalyzed reactions for the formation of GDP-6-deoxy-β-L-*galacto*-heptofuranoside (**32**) and GDP-6-deoxy-α-D-*altro* heptofuranoside (**31**) from D-sedoheptulose-7-phosphate (**1**).

HS:42

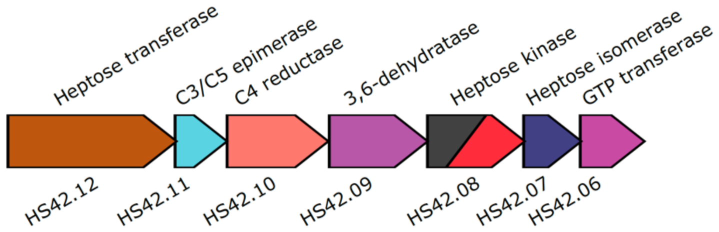

GDP-6-deoxy-β-L-*galacto*-heptose (**14**)

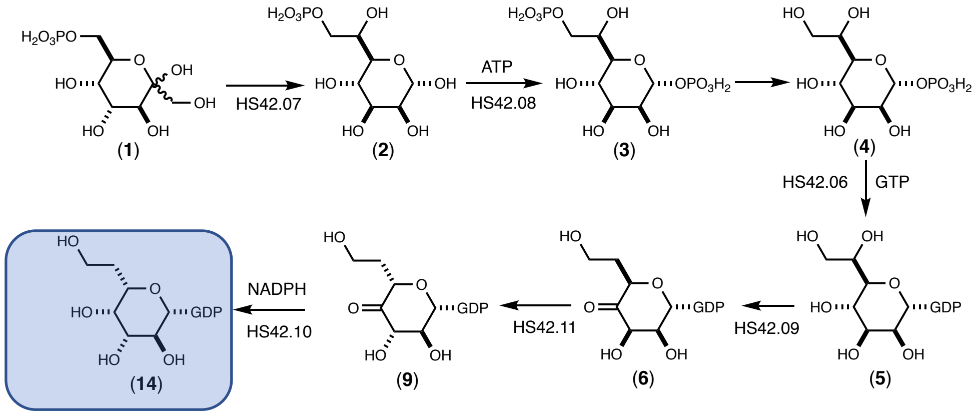

Figure S18: Gene cluster for GDP-heptose formation in the HS:42 serotype of *C. jejuni* (GenBank Accession id: HQ343274.1) and enzyme-catalyzed reactions for the formation of GDP-6-deoxy-β-L-*galacto*-heptose (14) from D-sedoheptulose-7-phosphate (**1**).

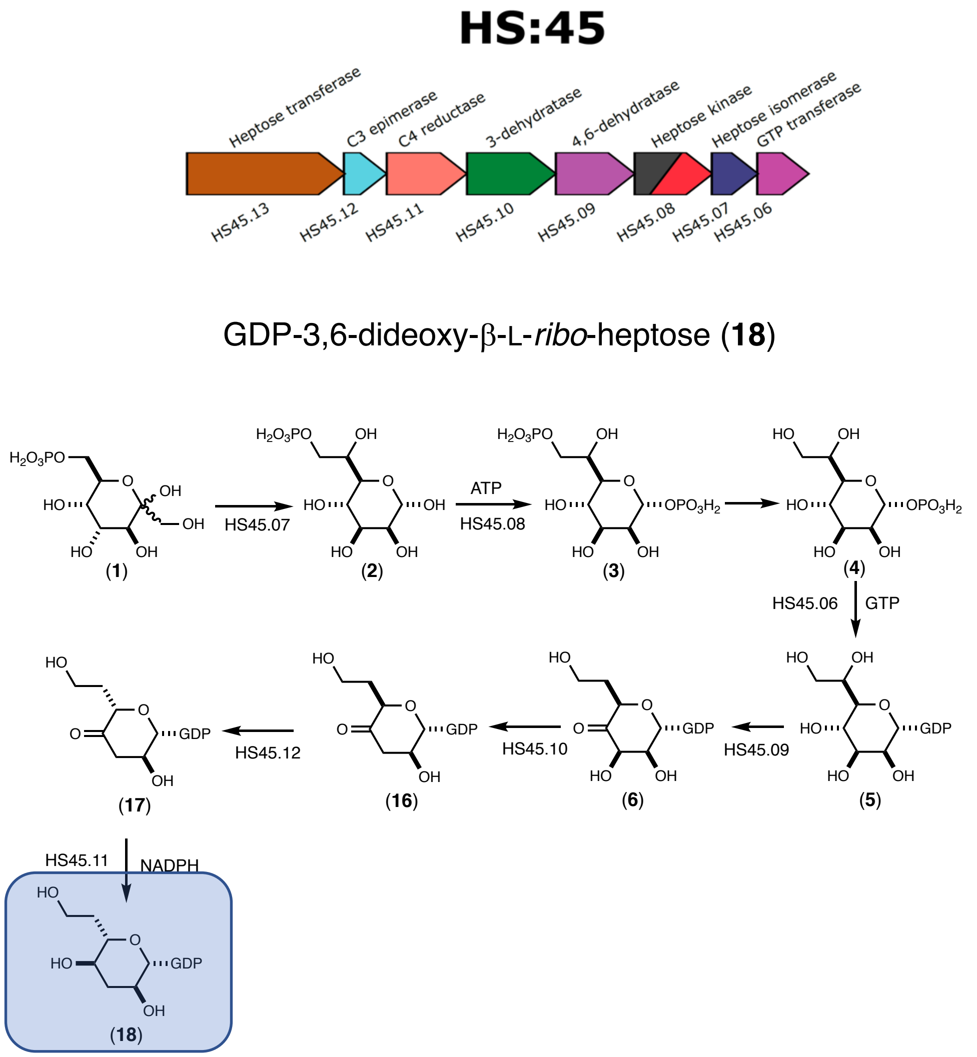

Figure S19: Gene cluster for GDP-heptose formation in the HS:45 serotype of *C. jejuni* (GenBank Accession id: KT893432.1) and enzyme-catalyzed reactions for the formation of GDP-3,6-dideoxy-β-L-*ribo*-heptose (**18**) from D-sedoheptulose-7-phosphate (**1**).

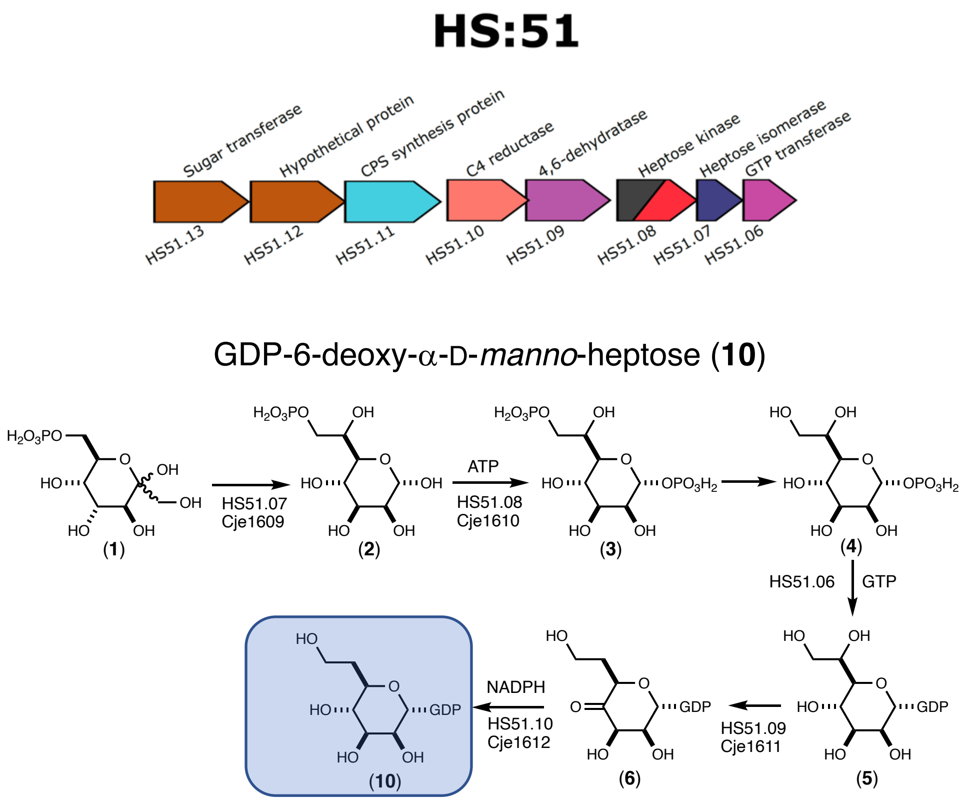

Figure S20: Gene cluster for GDP-heptose formation in the HS:51/HS:53 serotype of *C. jejuni* (GenBank Accession id: KT893432.1) and enzyme-catalyzed reactions for the formation of GDP-6-deoxy-α-D-*manno*-heptose (**10**) from D-sedoheptulose-7-phosphate (**1**).

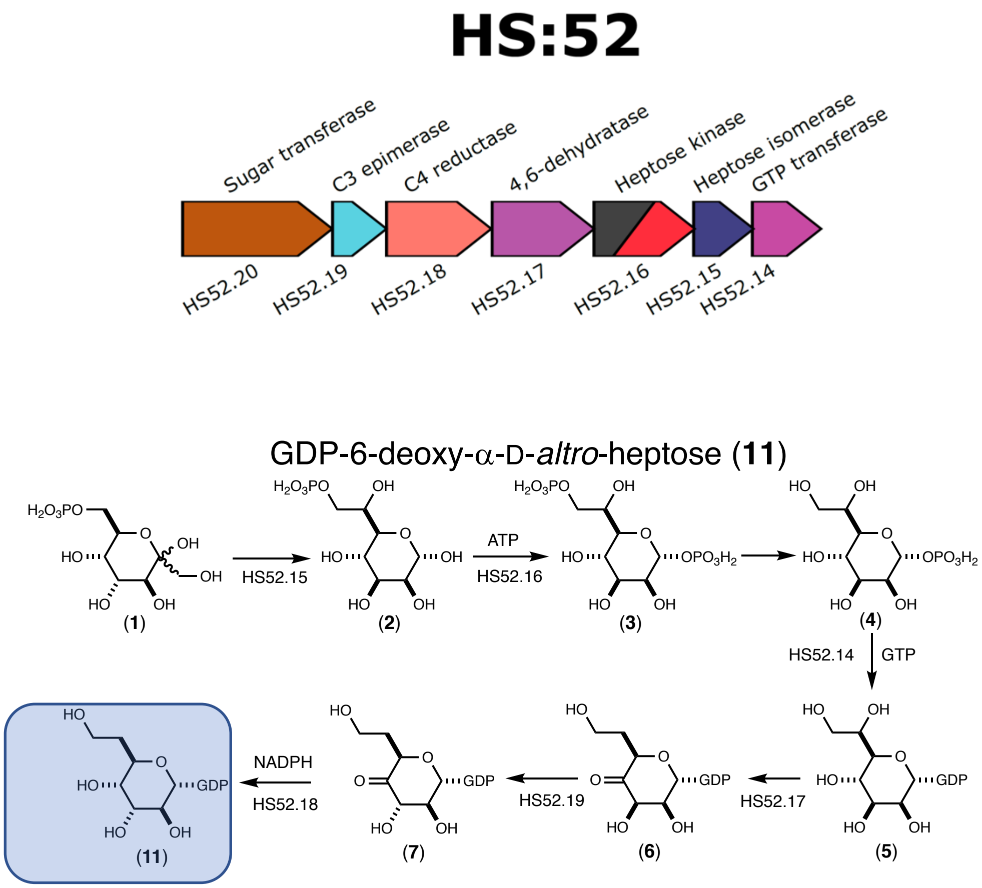

Figure S21: Gene cluster for GDP-heptose formation in the HS:52 serotype of *C. jejuni* (GenBank Accession id: KT893429.1) and enzyme-catalyzed reactions for the formation of GDP-6-deoxy-α-D-*altro*-heptose (**11**) from D-sedoheptulose-7-phosphate (**1**).

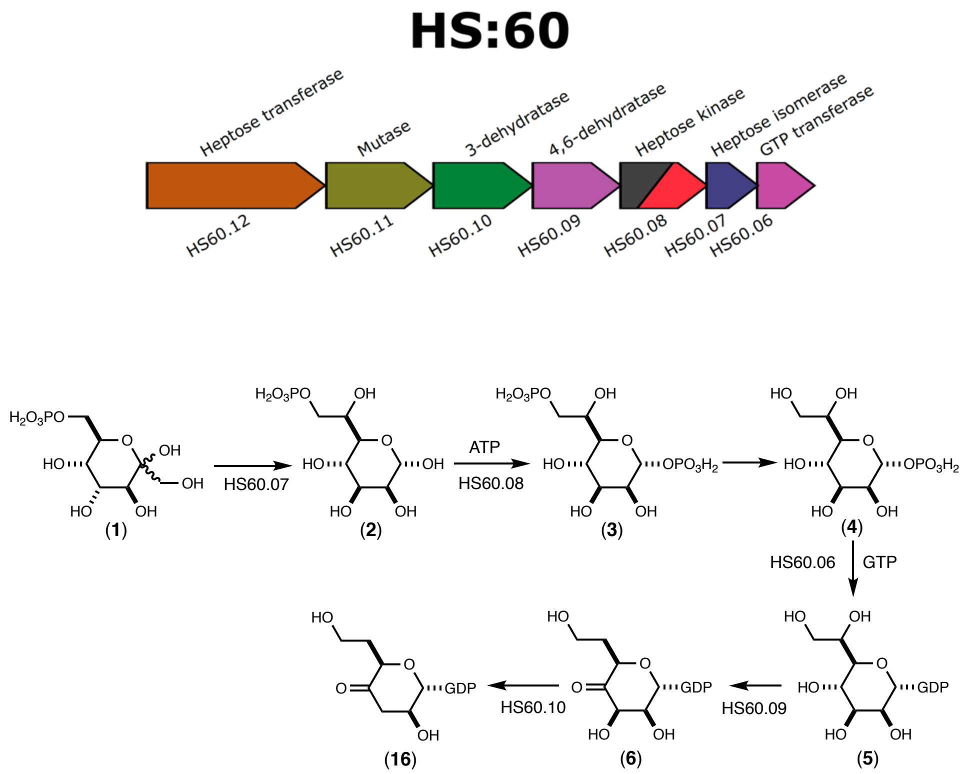

Figure S22: Gene cluster for GDP-heptose formation in the HS:60 serotype of *C. jejuni* (GenBank Accession id: KT893426.1). The final heptose cannot be determined since there is not an apparent C4-reductase.

HS:63

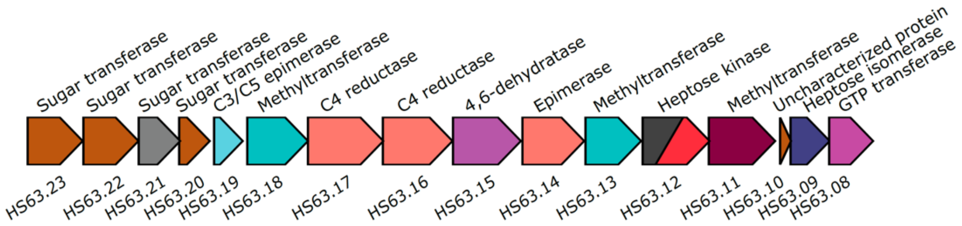

GDP-6-deoxy-β-L-*gluco*-heptose (15)
GDP-6-deoxy-β-L-*galacto*-heptose (14)

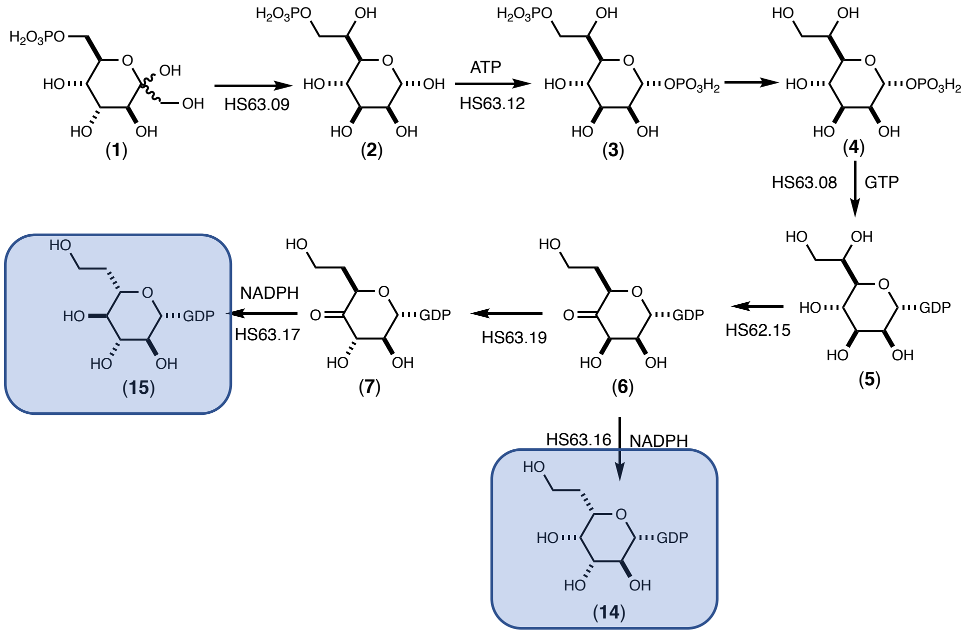

Notes

1. At the 3'-end of the gene for HS63.17 there is a poly-G tract that may influence whether HS63.17 or HS63.16 is expressed in a catalytically active form (*3*).

Figure S23: Gene cluster for GDP-heptose formation in the HS:63 serotype of *C. jejuni* (GenBank Accession id: KT893438.1) and enzyme-catalyzed reactions for the formation of GDP-6-deoxy-b-L-*gluco*-heptose (**15**) and GDP-6-deoxy-β-L-*galacto*-heptose (**1**4) from D-sedoheptulose-7-phosphate (**1**).

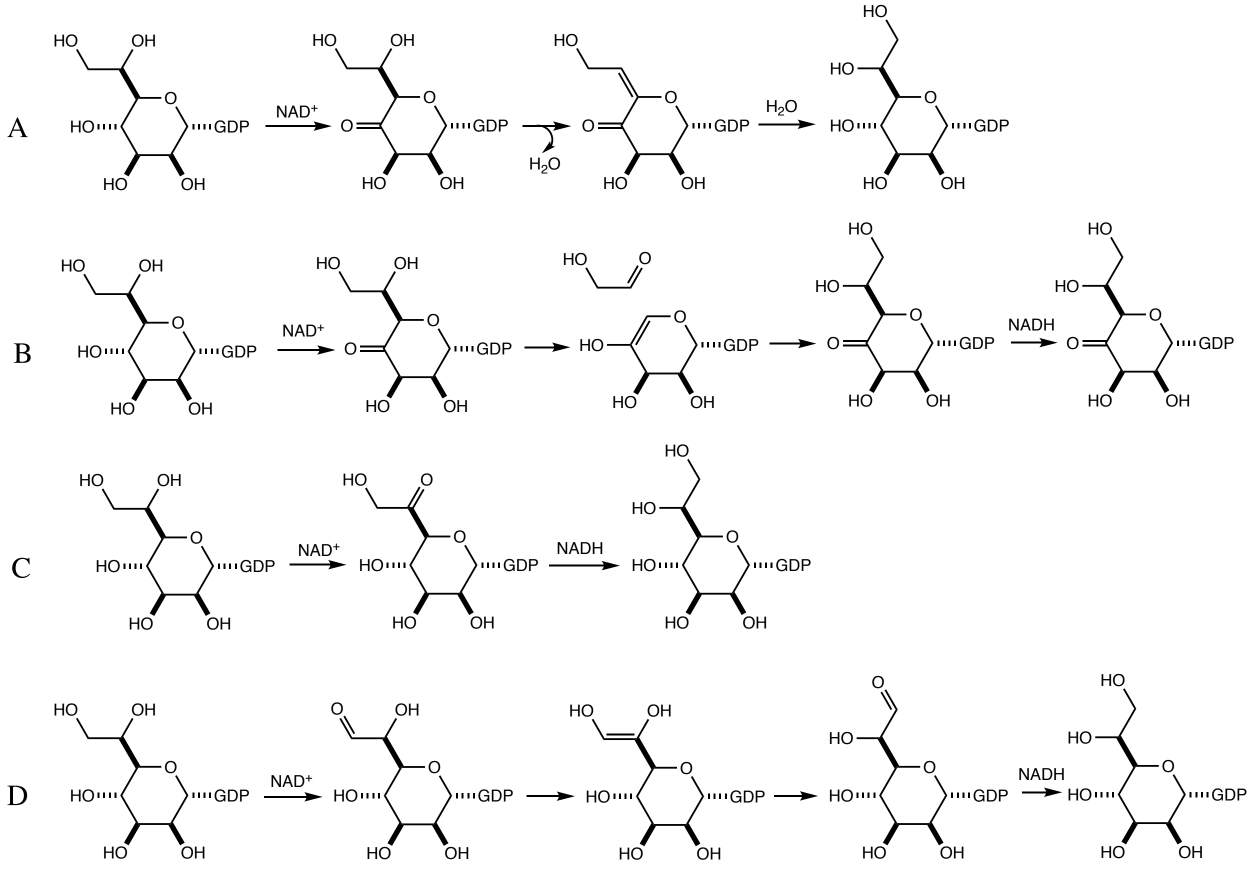

Scheme S1. Potential mechanisms for the enzymatic isomerization of C6 within GDP-D-*glycero*-α-D-*manno*-heptose.

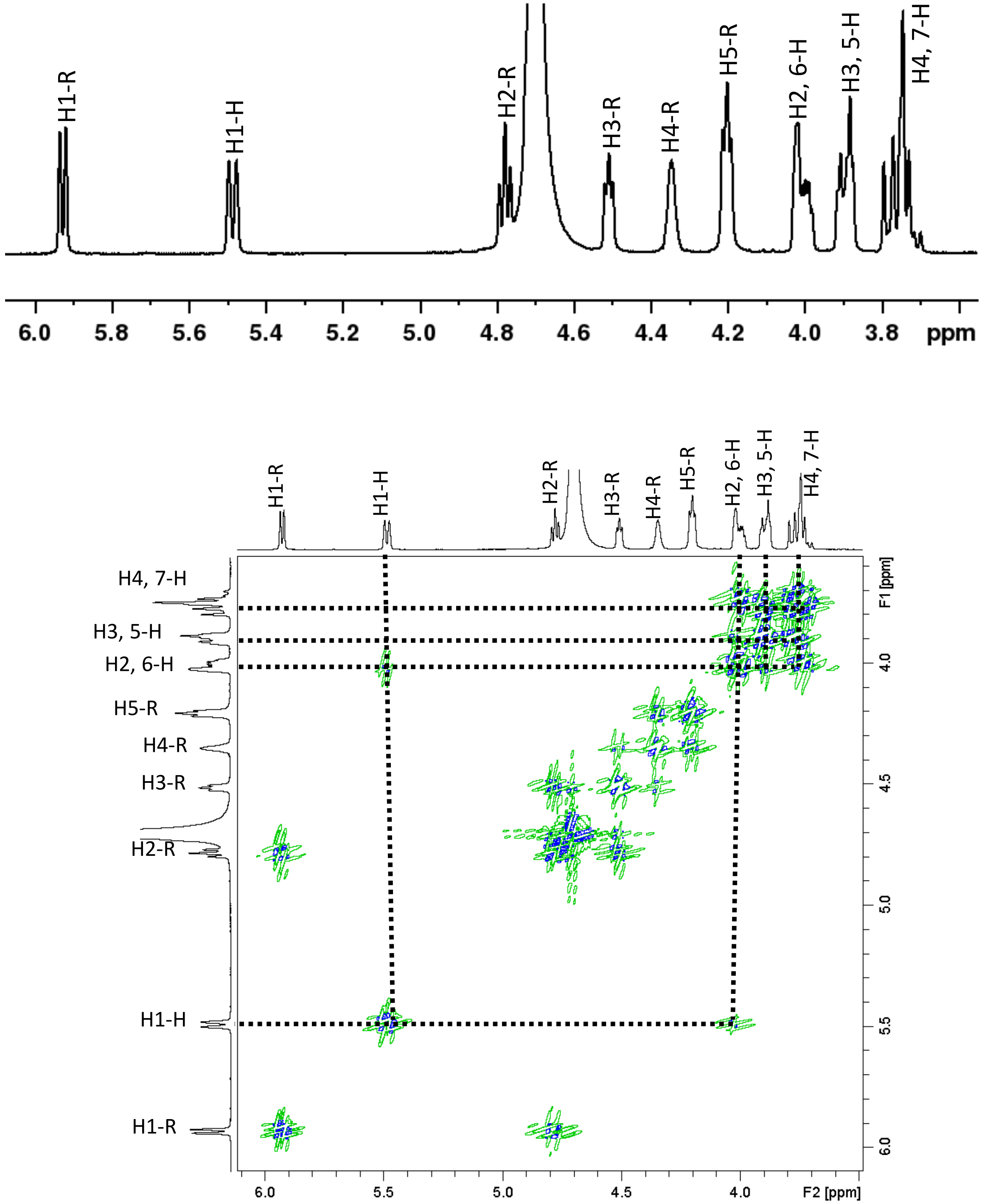

Figure S24. ^{1}H-NMR and ^{1}H-^{1}H COSY NMR spectra of GDP-D-*glycero*-α-D-*manno*-heptose (**5**). Resonances for the hydrogen labeled with an "R" correspond to the ribose moiety of GDP, while those labeled with an "H" correspond to those of the heptose moiety. (top) ^{1}H NMR spectra; (bottom)^{1}H-^{1}H COSY NMR spectrum

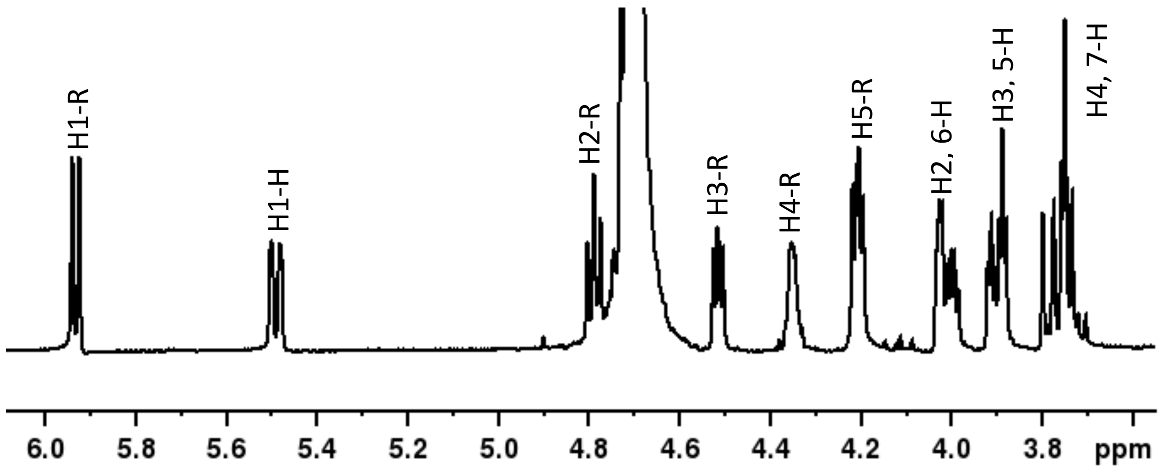

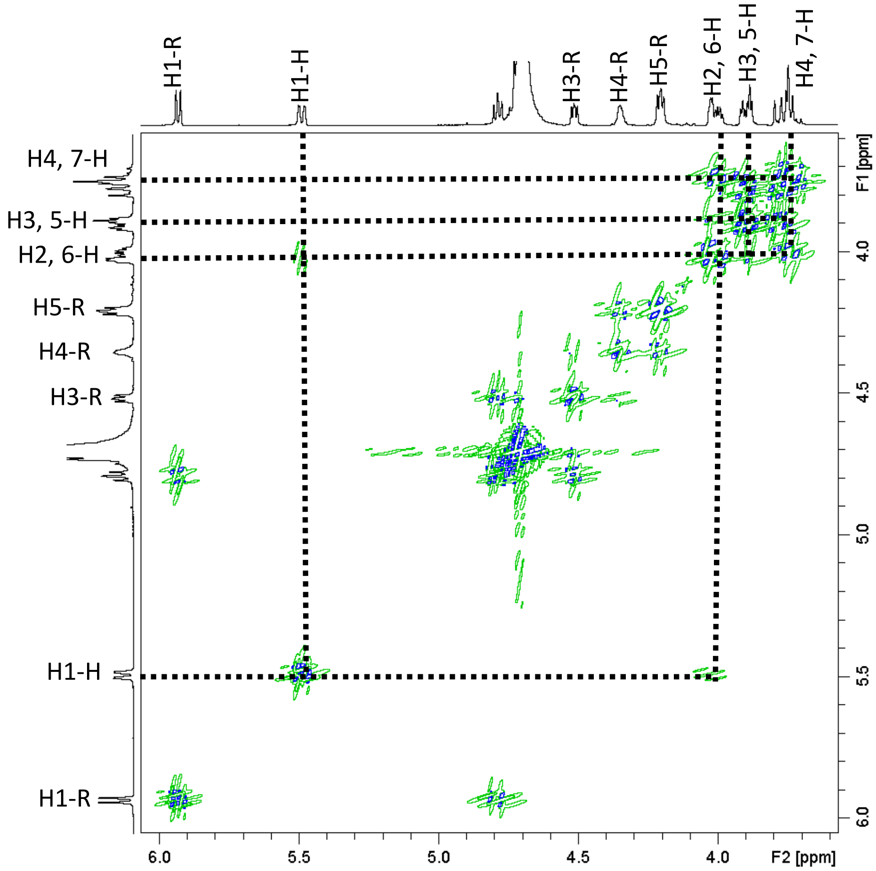

Figure S25. ^{1}H NMR and ^{1}H-^{1}H COSY NMR spectra of **5** produced by the reduction of **22** with the C4-reductase from serotype HS:53 in H_2O. Resonances for the hydrogen labeled with an "R" correspond to the ribose moiety of GDP, while those labeled with an "H" correspond to those of the heptose moiety. (top) ^{1}H NMR spectra; (bottom) ^{1}H-^{1}H COSY NMR spectra.

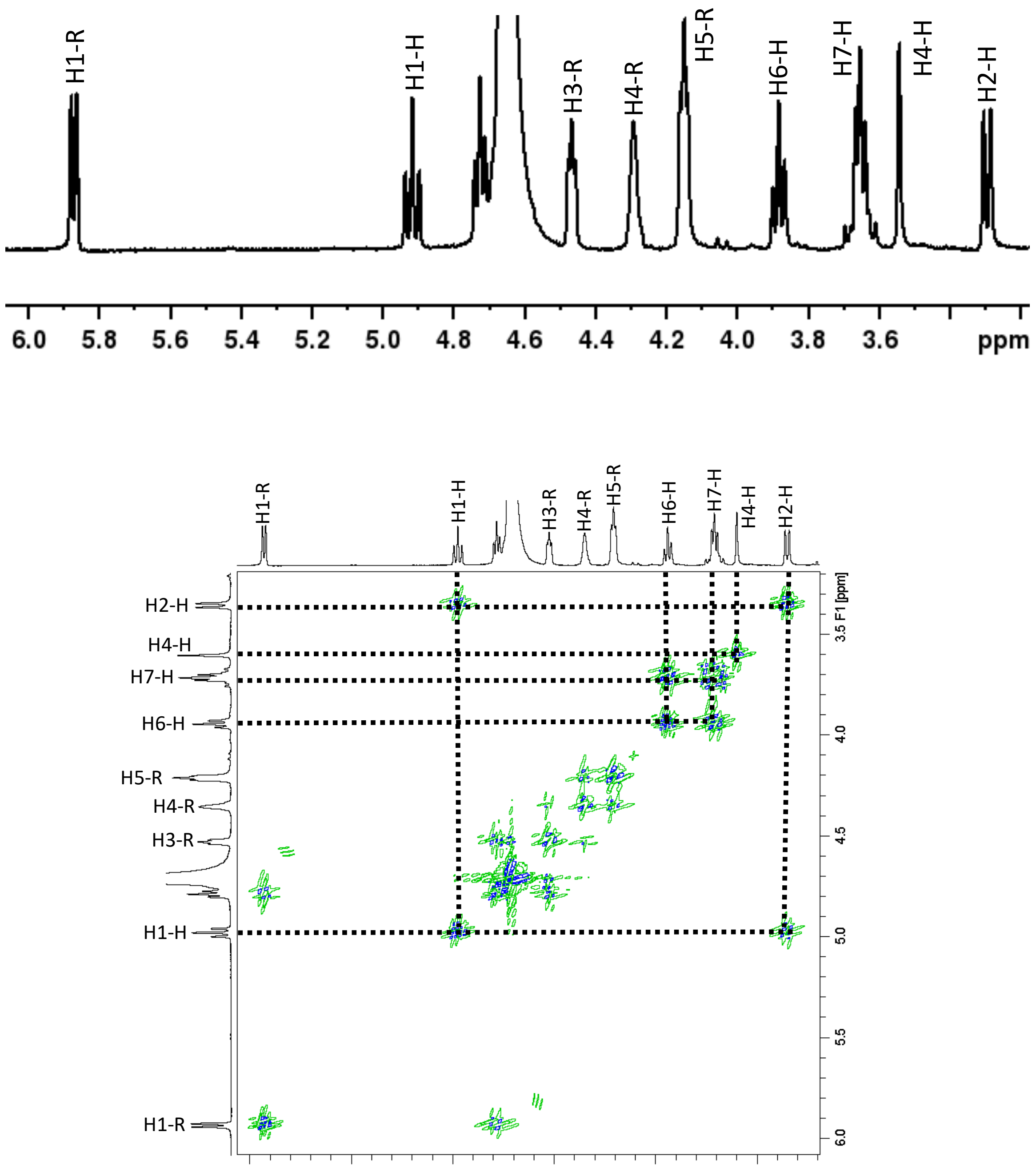

Figure S26. ^{1}H NMR and ^{1}H-^{1}H COSY NMR spectra of compound **26** formed from the reduction of compound **25** by the C4-reductase from serotype HS:2 in D_2O. Resonances for the hydrogen labeled with an "R" correspond to the ribose moiety of GDP, while those labeled with an "H" correspond to those of the heptose moiety. (top) ^{1}H NMR spectra. (bottom) ^{1}H-^{1}H COSY NMR spectrum.

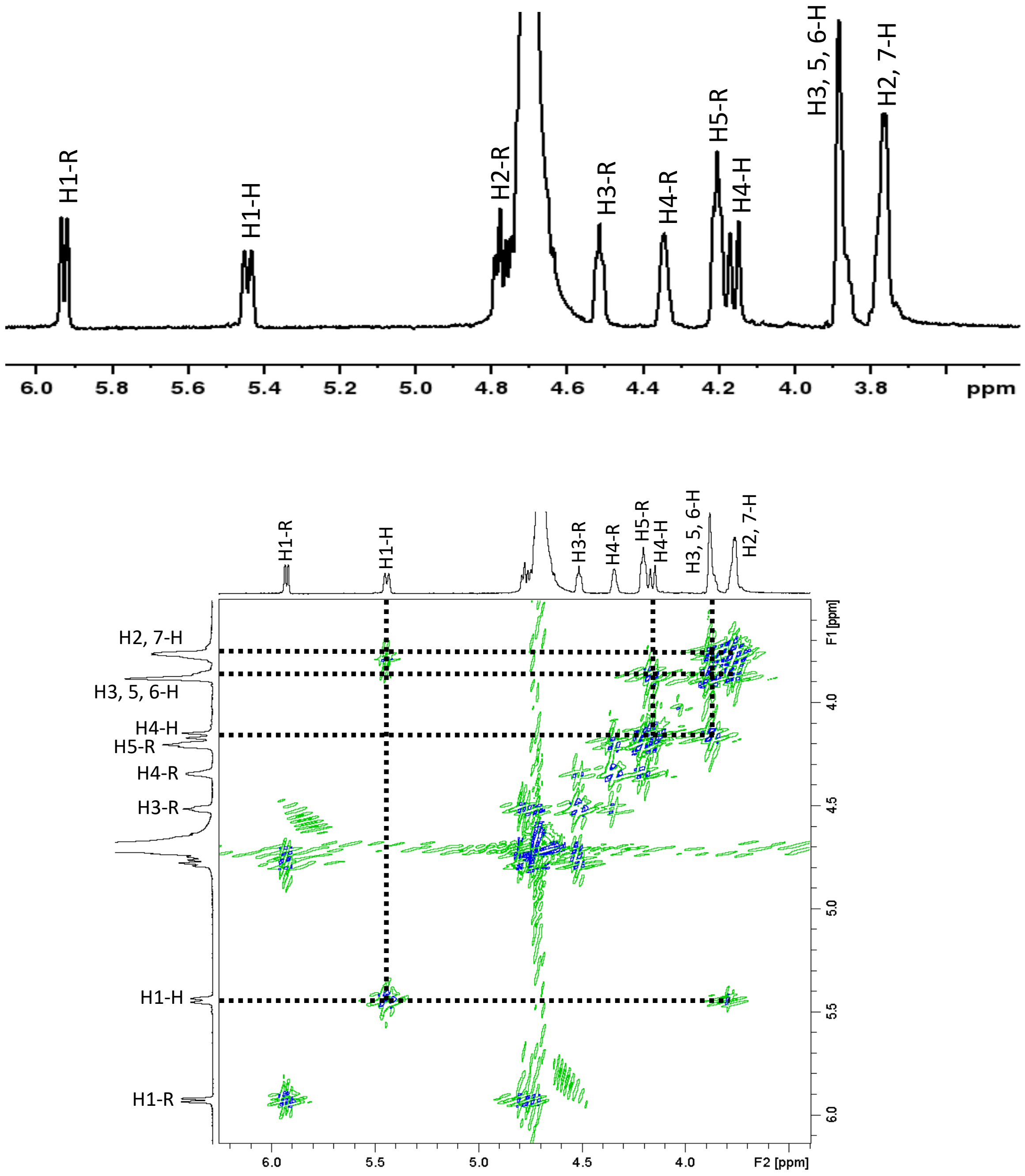

Figure S27. ^1H NMR and ^1H-^1H COSY NMR spectra of compound **28** formed by the reduction of compound **23** by the C4-reductase from HS:3 in H_2O. Resonances for the hydrogen labeled with an "R" correspond to the ribose moiety of GDP, while those labeled with an "H" correspond to those of the heptose moiety. (top) ^1H NMR spectrum; (bottom) ^1H-^1H COSY NMR spectrum.

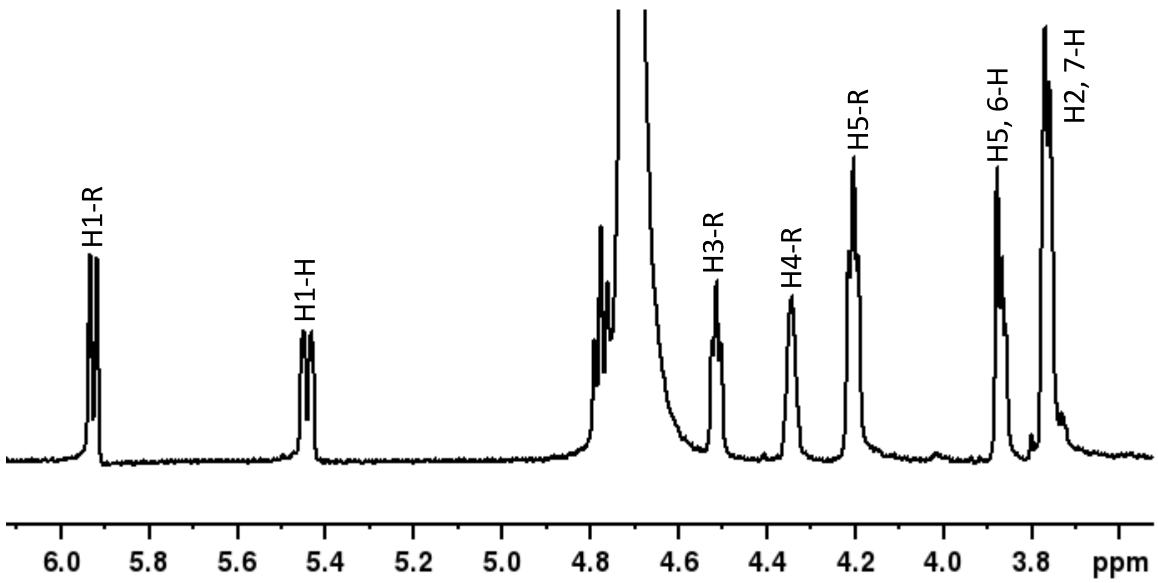

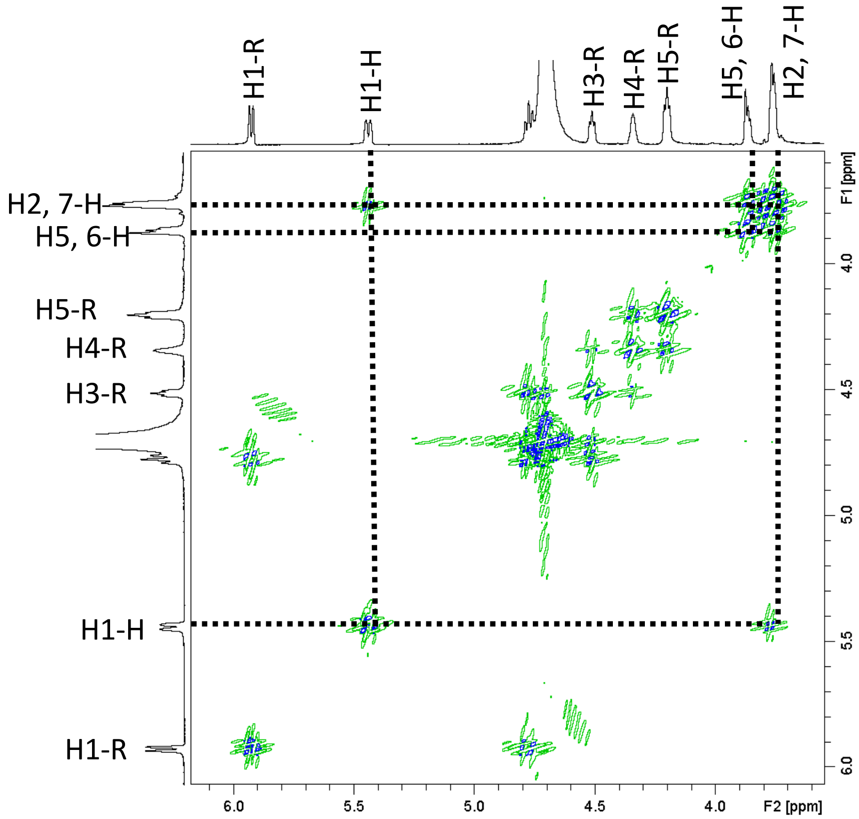

Figure S28. ^1H NMR and ^1H-^1H COSY NMR spectra of compound **28** formed by the reduction of compound **23** by the C4-reductase from HS:3 in D_2O. Resonances for the hydrogen labeled with an "R" correspond to the ribose moiety of GDP, while those labeled with an "H" correspond to those of the heptose moiety. (top) ^1H NMR spectrum. (bottom) ^1H-^1H COSY NMR spectrum.

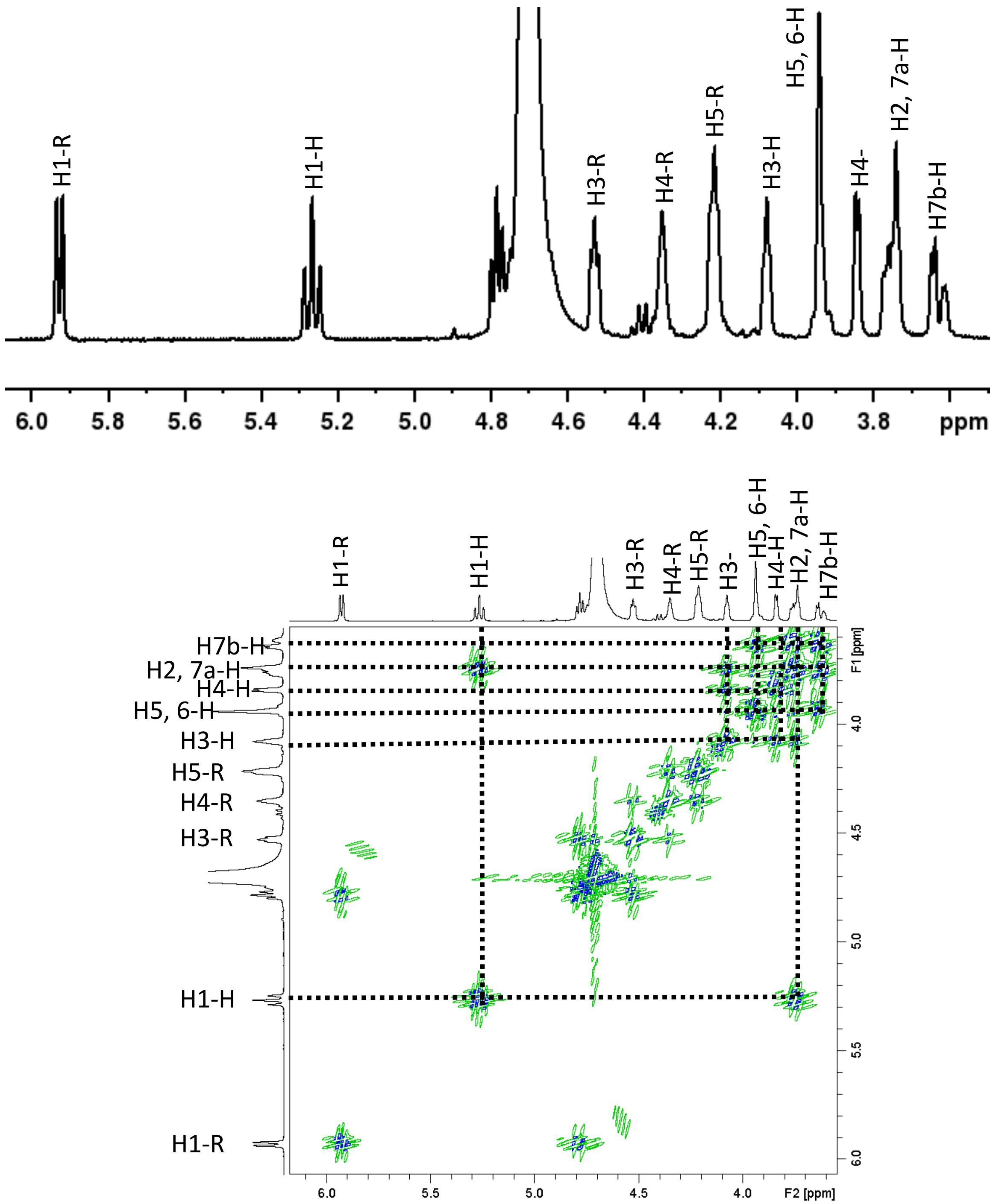

Figure S29. ^1H NMR and ^1H-^1H COSY NMR spectra of compound **29** formed by the reduction of compound **24** by the C4-reductase from HS:15 in H_2O. Resonances for the hydrogen labeled with an "R" correspond to the ribose moiety of GDP, while those labeled with an "H" correspond to those of the heptose moiety. (top) ^1H NMR spectra; (bottom) ^1H-^1H COSY NMR spectra.

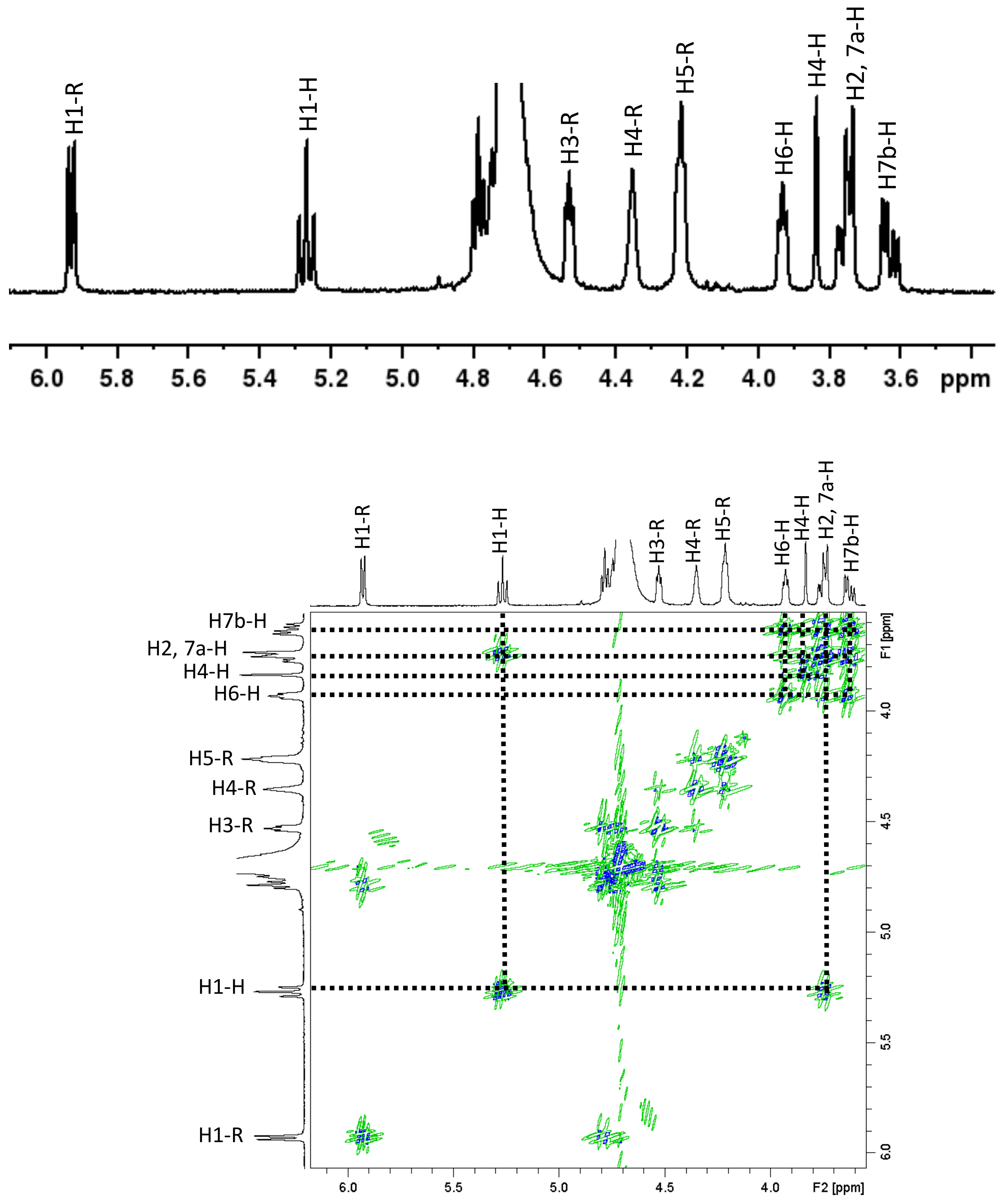

Figure S30. ^{1}H NMR and ^{1}H-^{1}H COSY NMR spectra of compound **29** formed by the reduction of compound **24** by the C4-reductase from HS:15 in D_2O. Resonances for the hydrogen labeled with an "R" correspond to the ribose moiety of GDP, while those labeled with an "H" correspond to those of the heptose moiety. (top) ^{1}H NMR spectra; (bottom) ^{1}H-^{1}H COSY NMR spectra.

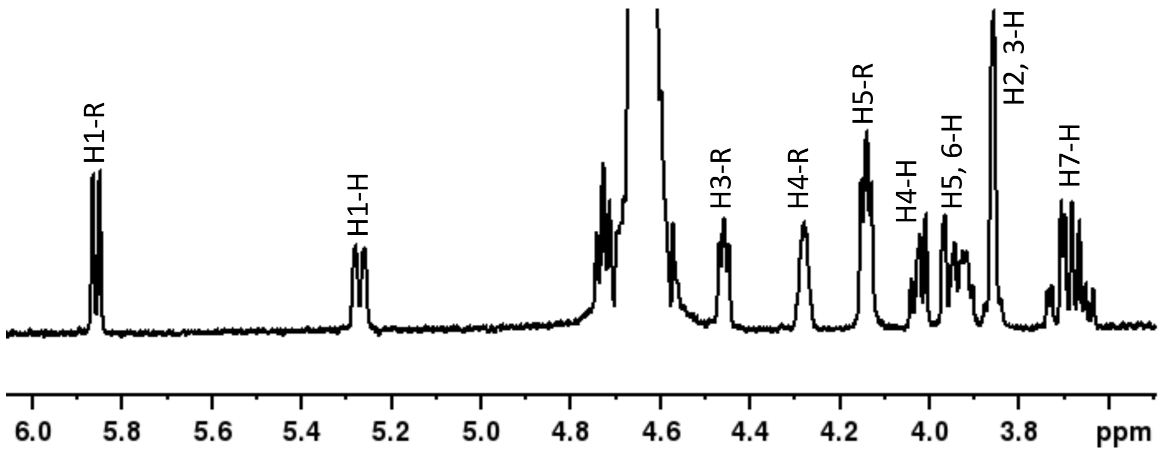

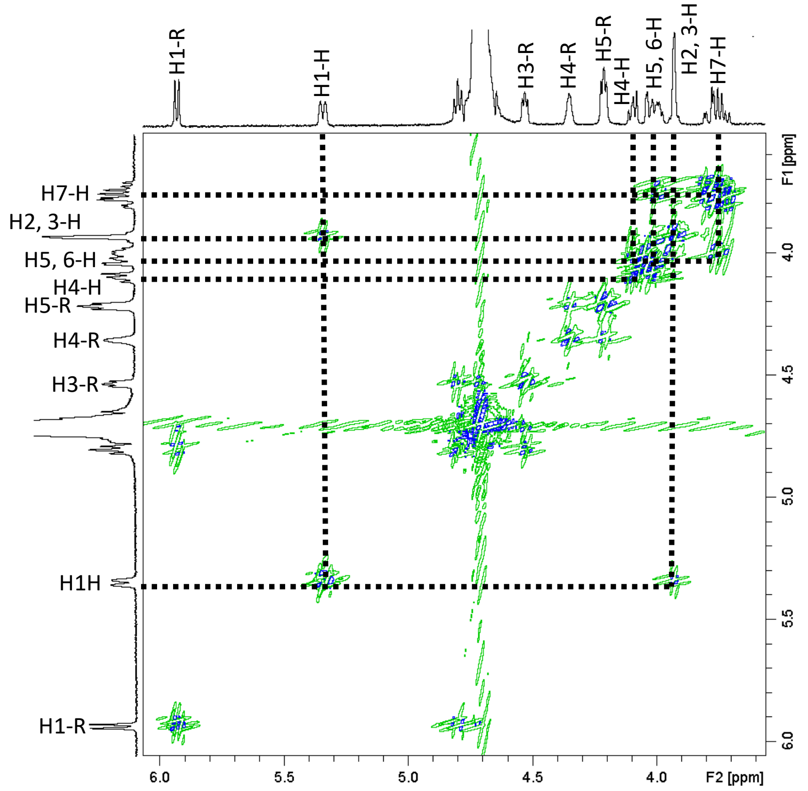

Figure S31. ^{1}H NMR and ^{1}H-^{1}H COSY NMR spectra of compound **27** formed by the reduction of compound **23** by the C4-reductase from HS:23/36 in H_2O. Resonances for the hydrogen labeled with an "R" correspond to the ribose moiety of GDP, while those labeled with an "H" correspond to those of the heptose moiety. (top) ^{1}H NMR spectrum; (bottom) ^{1}H-^{1}H COSY NMR spectrum.

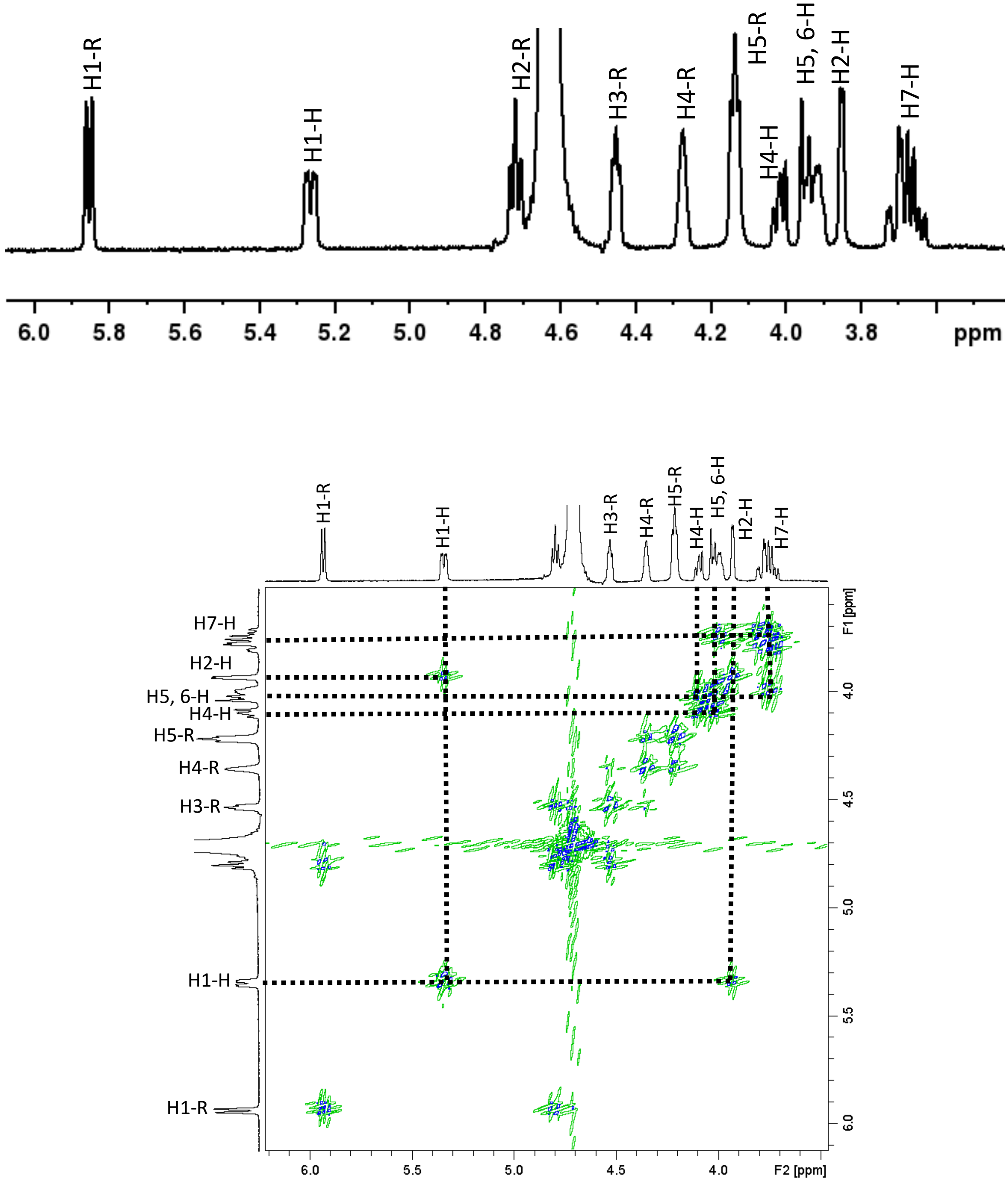

Figure S32. ^{1}H NMR and ^{1}H-^{1}H COSY NMR spectra of compound **27** formed by the reduction of compound **23** by the C4-reductase from HS:23/36 in D_2O. Resonances for the hydrogen labeled with an "R" correspond to the ribose moiety of GDP, while those labeled with an "H" correspond to those of the heptose moiety. (top) ^{1}H NMR spectrum; (bottom) ^{1}H-^{1}H COSY NMR spectrum.

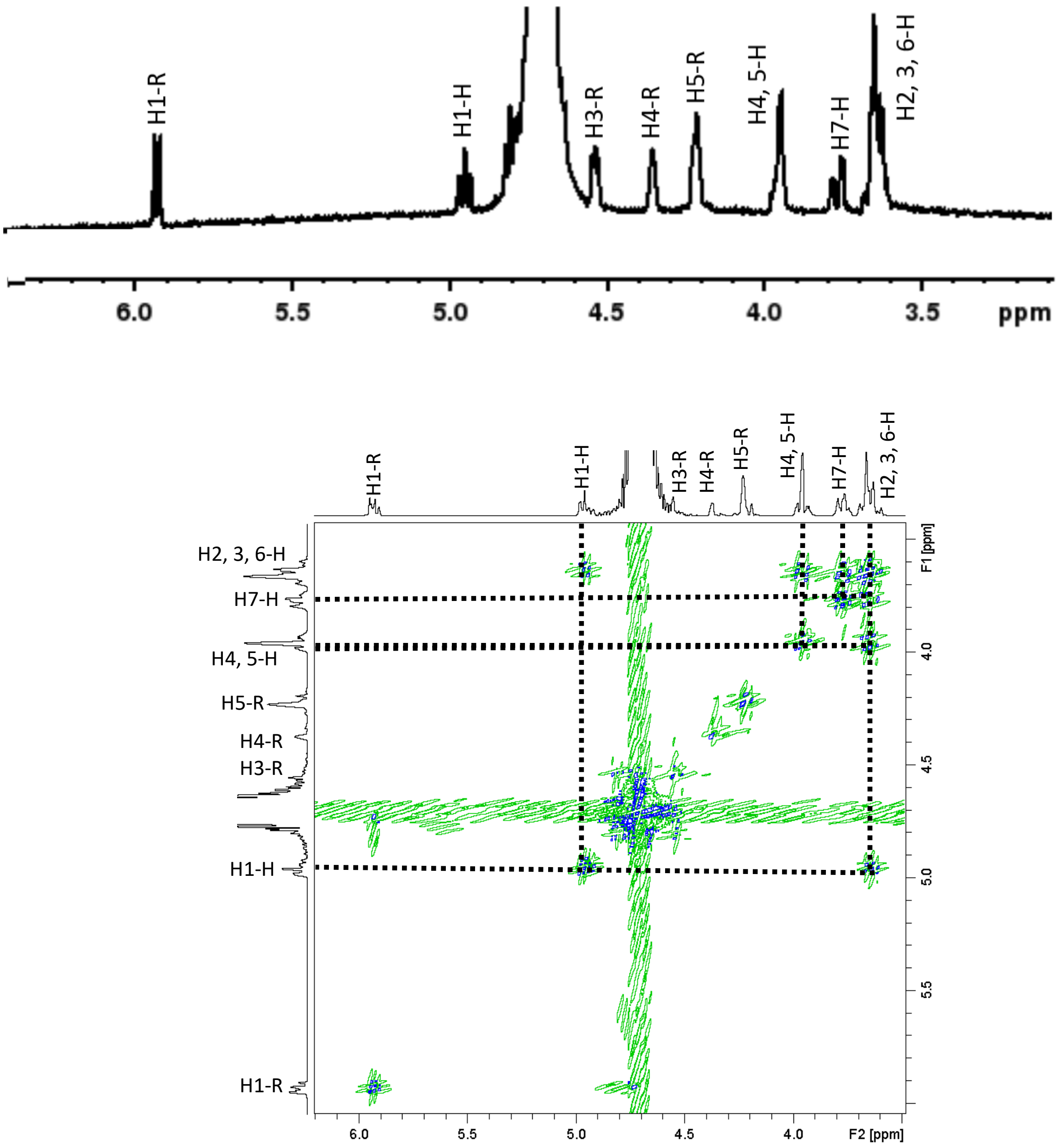

Figure S33. ^{1}H NMR and ^{1}H-^{1}H COSY NMR spectra of compound 30 formed from the reduction of compound 25 by the C4-reductase from serotype HS:42 in H_2O. Resonances for the hydrogen labeled with an "R" correspond to the ribose moiety of GDP, while those labeled with an "H" correspond to those of the heptose moiety. (top) ^{1}H NMR spectra; (bottom) ^{1}H-^{1}H COSY NMR spectra.

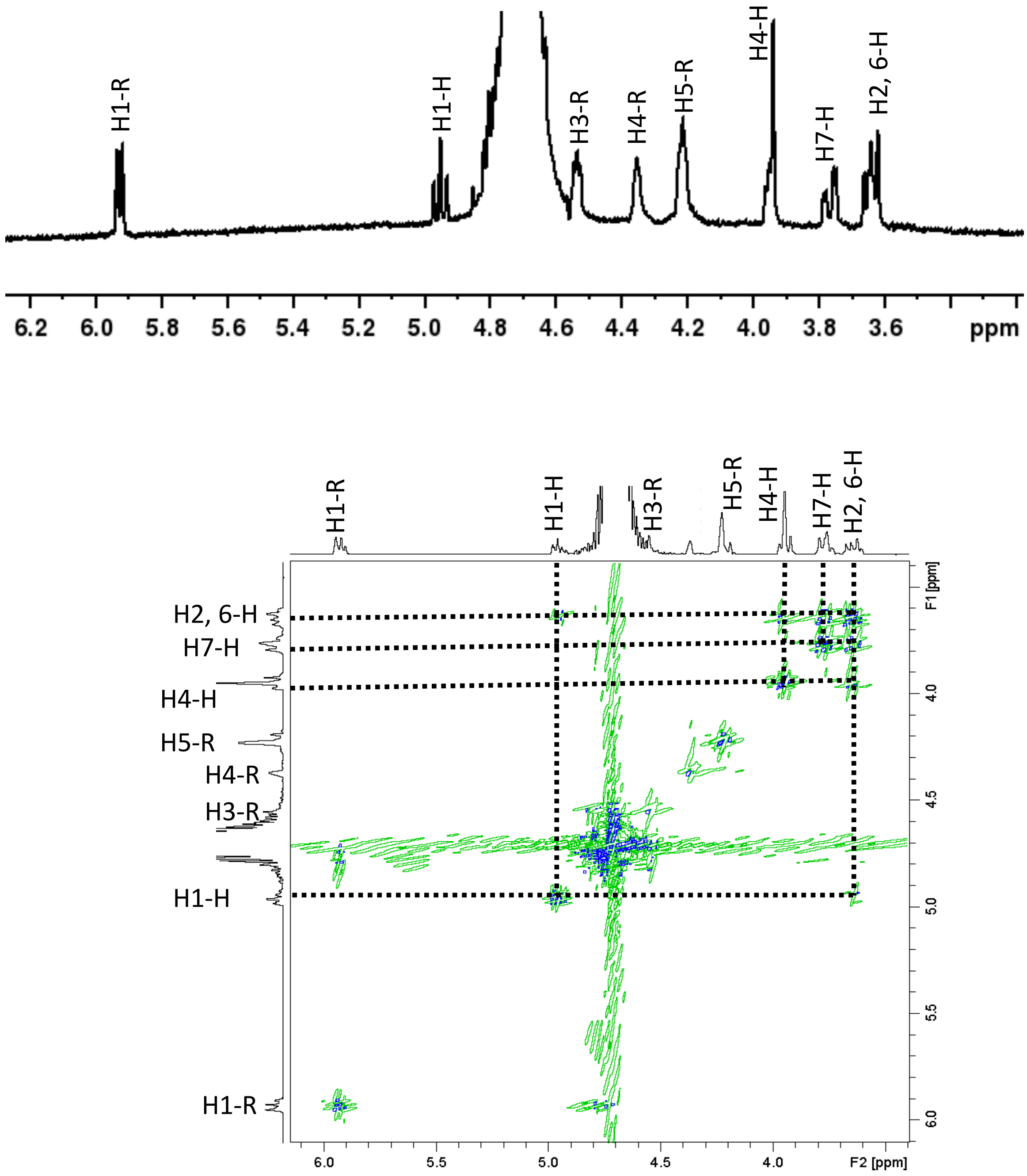

Figure S34. ^{1}H NMR and ^{1}H-^{1}H COSY NMR spectra of compound **30** formed from the reduction of compound **25** by the C4-reductase from serotype HS:42 in D_2O. Resonances for the hydrogen labeled with an "R" correspond to the ribose moiety of GDP, while those labeled with an "H" correspond to those of the heptose moiety. (top) ^{1}H NMR spectrum; (bottom) ^{1}H-^{1}H COSY NMR spectrum.

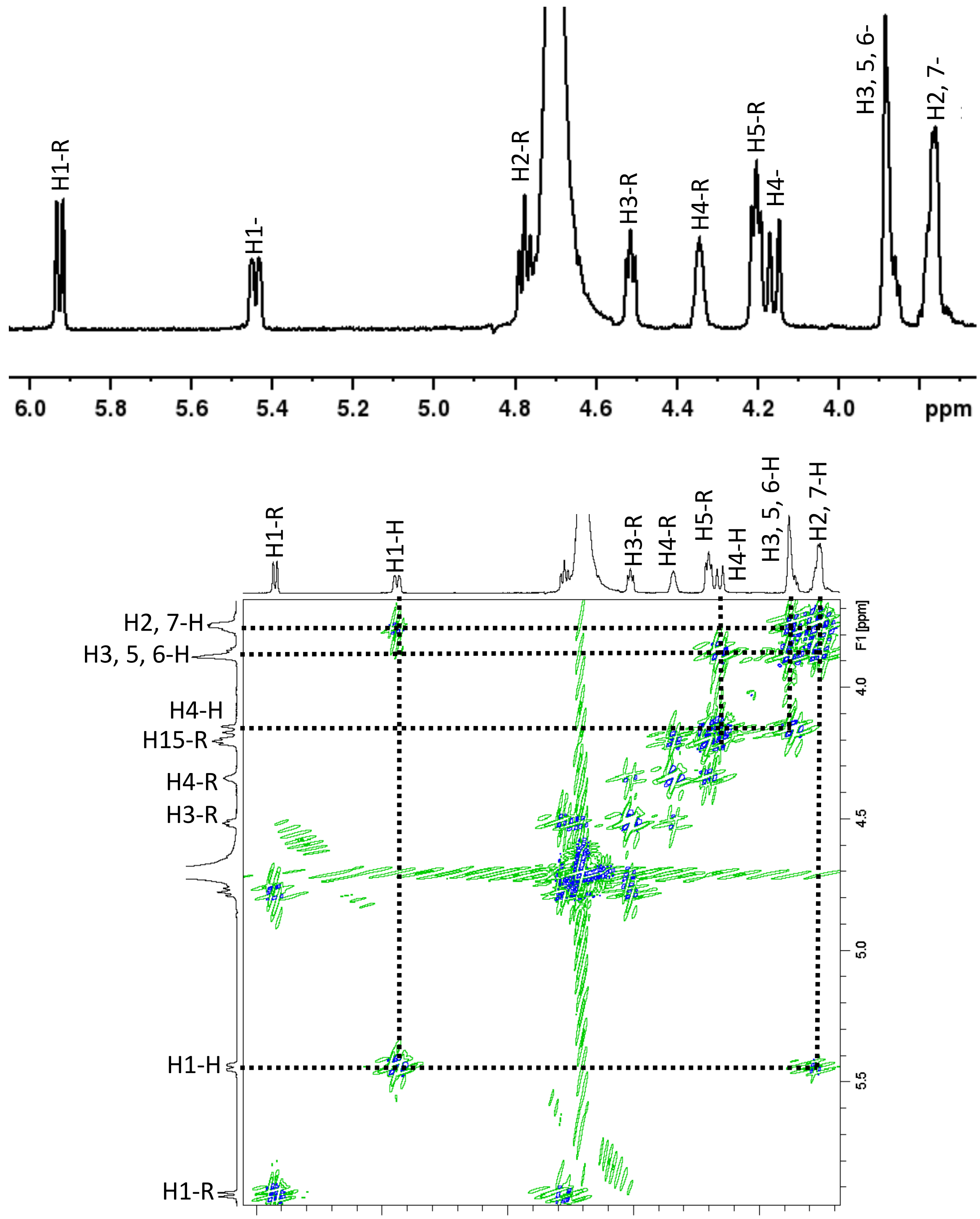

Figure S35. ${}^{1}H$ NMR and ${}^{1}H$-${}^{1}H$ COSY NMR spectra of compound **28** formed by the reduction of compound **23** by the C4-reductase from HS:33 in H_2O. Resonances for the hydrogen labeled with an "R" correspond to the ribose moiety of GDP, while those labeled with an "H" correspond to those of the heptose moiety. (top) ${}^{1}H$ NMR spectrum; (bottom) ${}^{1}H$-${}^{1}H$ COSY NMR spectrum.

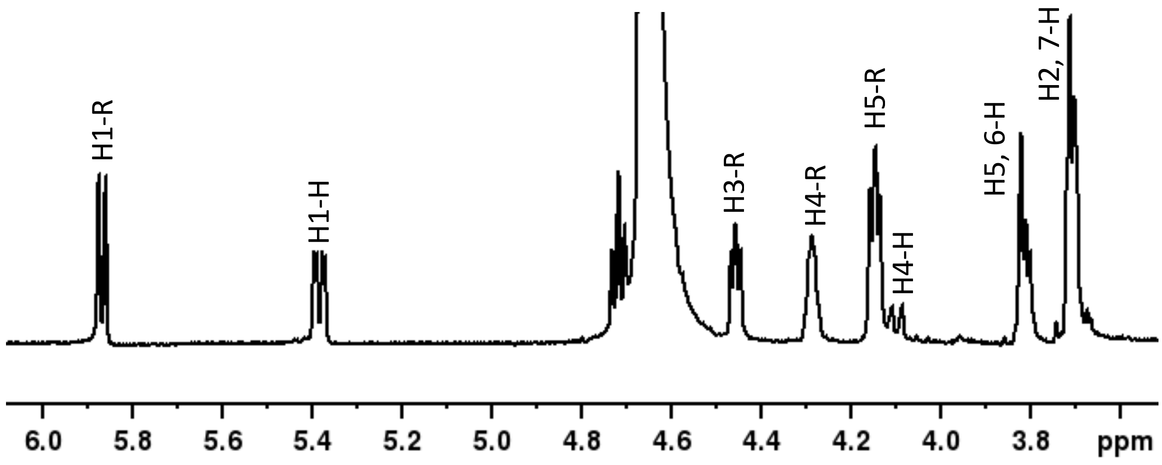

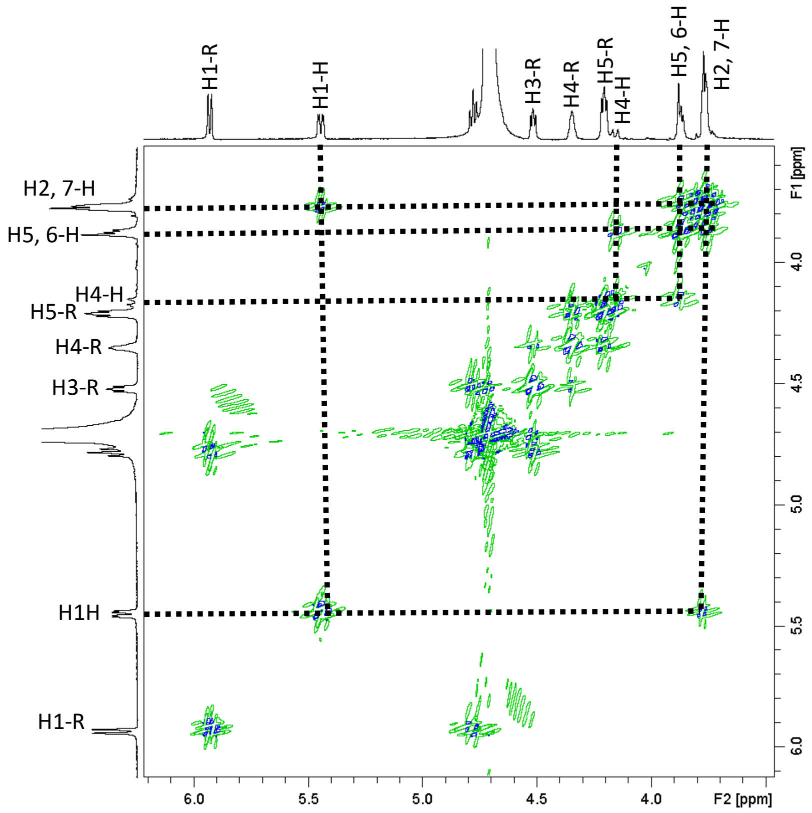

Figure S36. ^{1}H NMR and ^{1}H-^{1}H COSY NMR spectra of compound **28** formed by the reduction of compound **23** by the C4-reductase from HS:3 in D_2O. Resonances for the hydrogen labeled with an "R" correspond to the ribose moiety of GDP, while those labeled with an "H" correspond to those of the heptose moiety. (top) ^{1}H NMR spectrum; (bottom) ^{1}H-^{1}H COSY NMR spectrum.

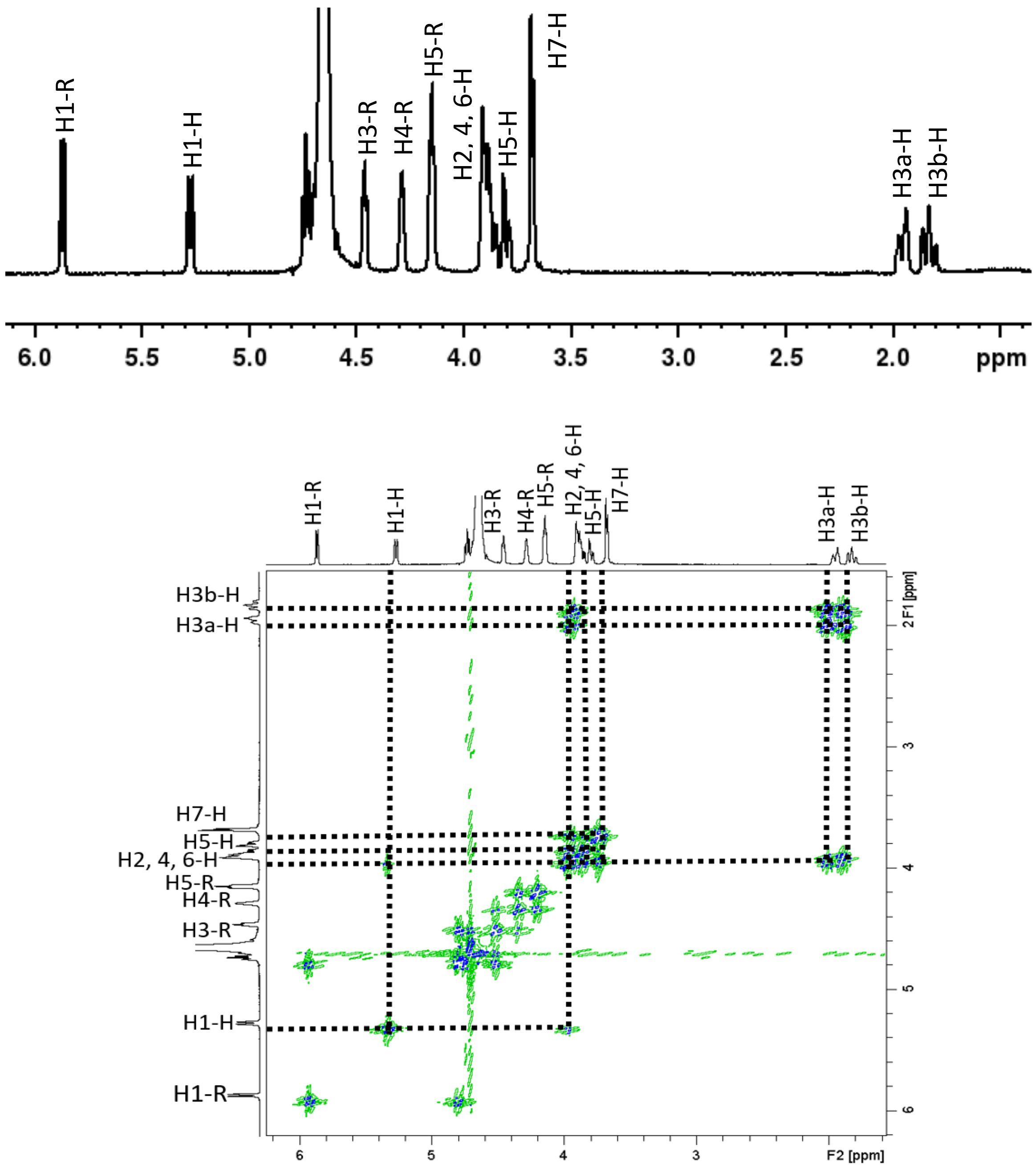

Figure S37. ^{1}H NMR and ^{1}H-^{1}H COSY NMR spectra of compound **34** formed by the reduction of compound **33** by the C4-reductase from HS:53 in H_2O. Resonances for the hydrogen labeled with an "R" correspond to the ribose moiety of GDP, while those labeled with an "H" correspond to those of the heptose moiety. (top) ^{1}H NMR spectrum;. (bottom) ^{1}H-^{1}H COSY NMR spectrum.

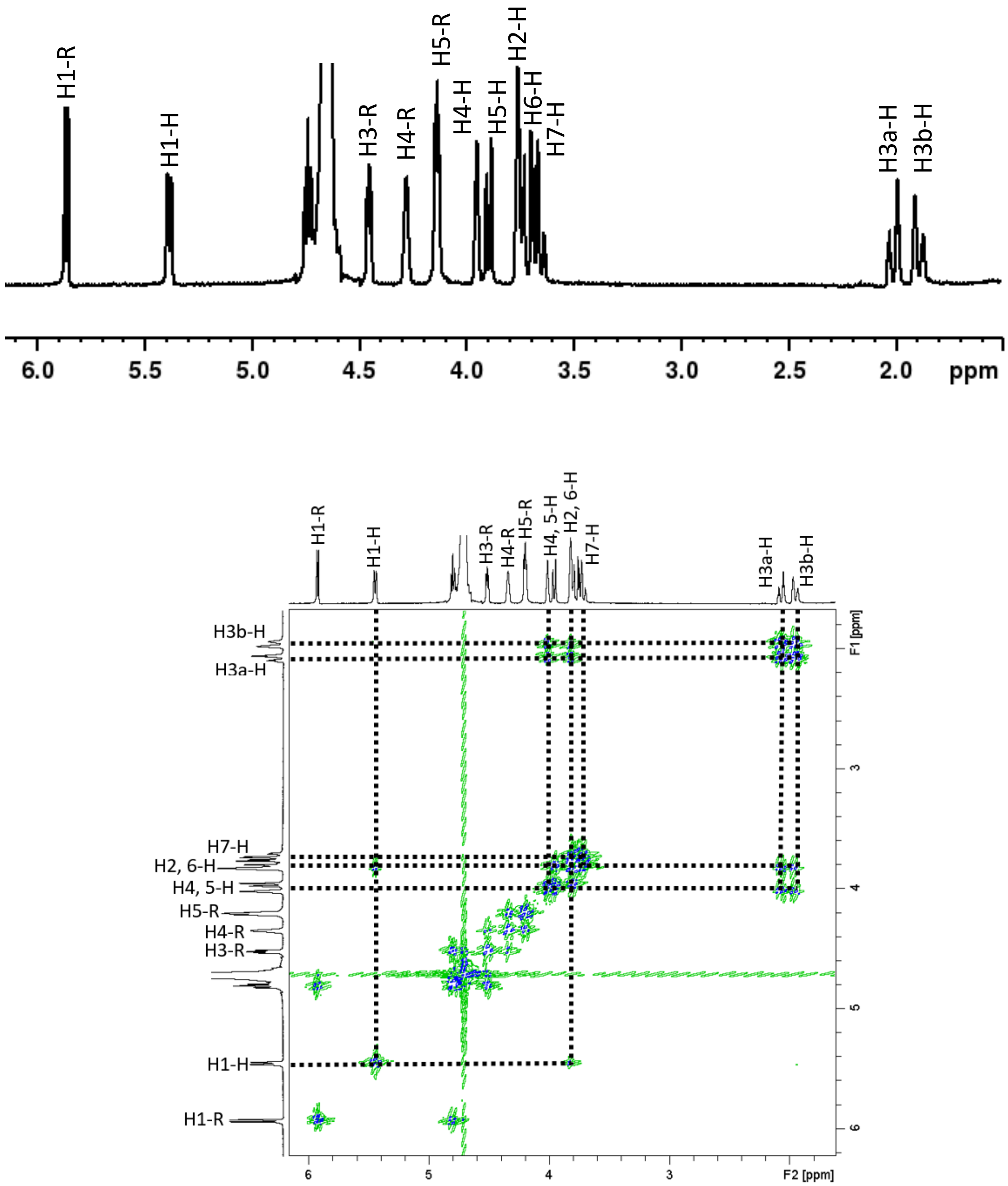

Figure S38. ^{1}H NMR and ^{1}H-^{1}H COSY NMR spectra of compound **35** formed by the reduction of compound **33** by the C4-reductase from HS:3 in H_2O. Resonances for the hydrogen labeled with an "R" correspond to the ribose moiety of GDP, while those labeled with an "H" correspond to those of the heptose moiety. (top) ^{1}H NMR spectra. (bottom) ^{1}H-^{1}H COSY NMR spectrum.

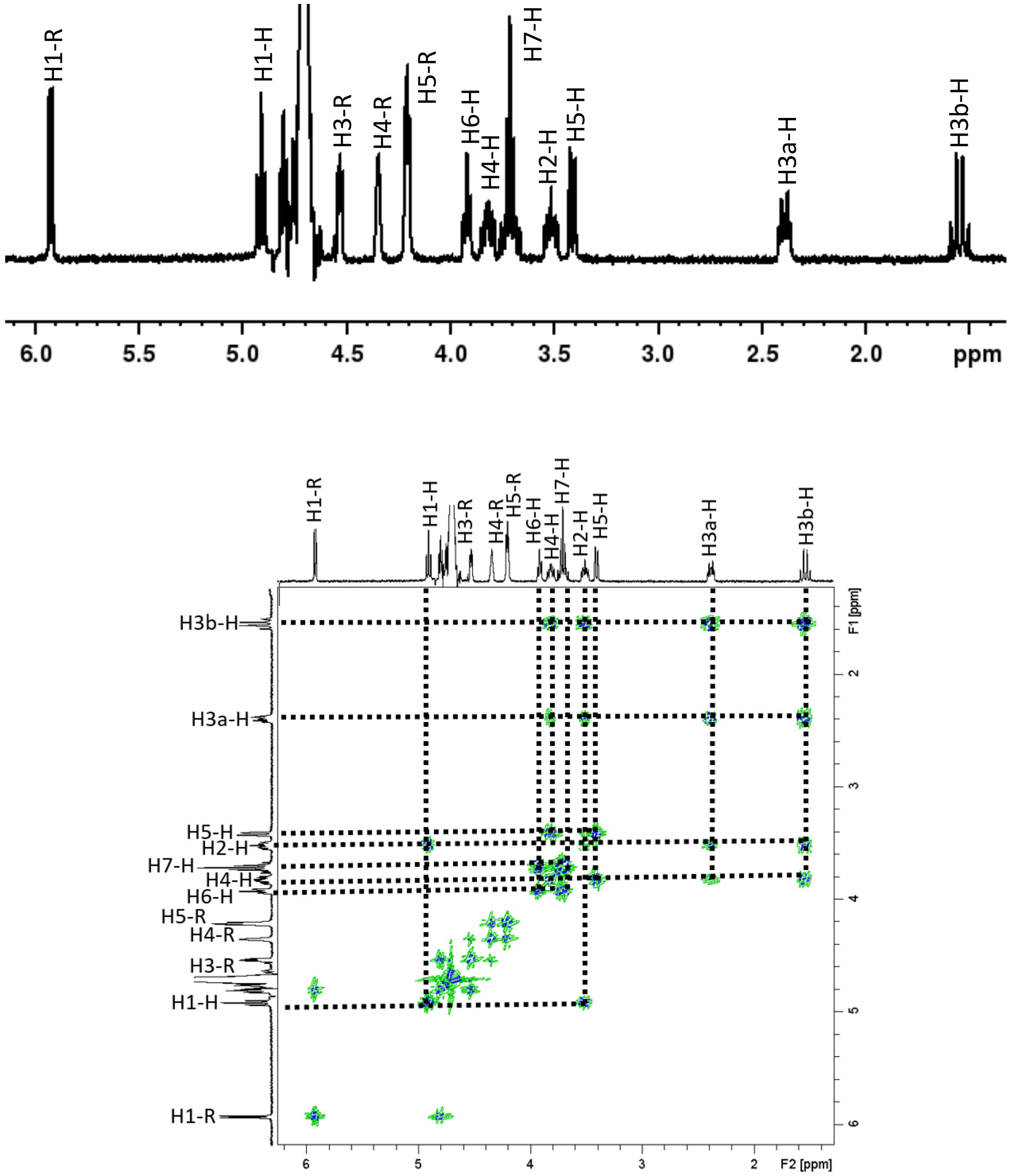

Figure S39. ^{1}H NMR and ^{1}H-^{1}H COSY NMR spectra of compound **37** formed by the reduction of compound **36** by the C4-reductase from HS:2 in H_2O. Resonances for the hydrogen labeled with an "R" correspond to the ribose moiety of GDP, while those labeled with an "H" correspond to those of the heptose moiety. (top) ^{1}H NMR spectra. (bottom) ^{1}H-^{1}H COSY NMR spectrum.

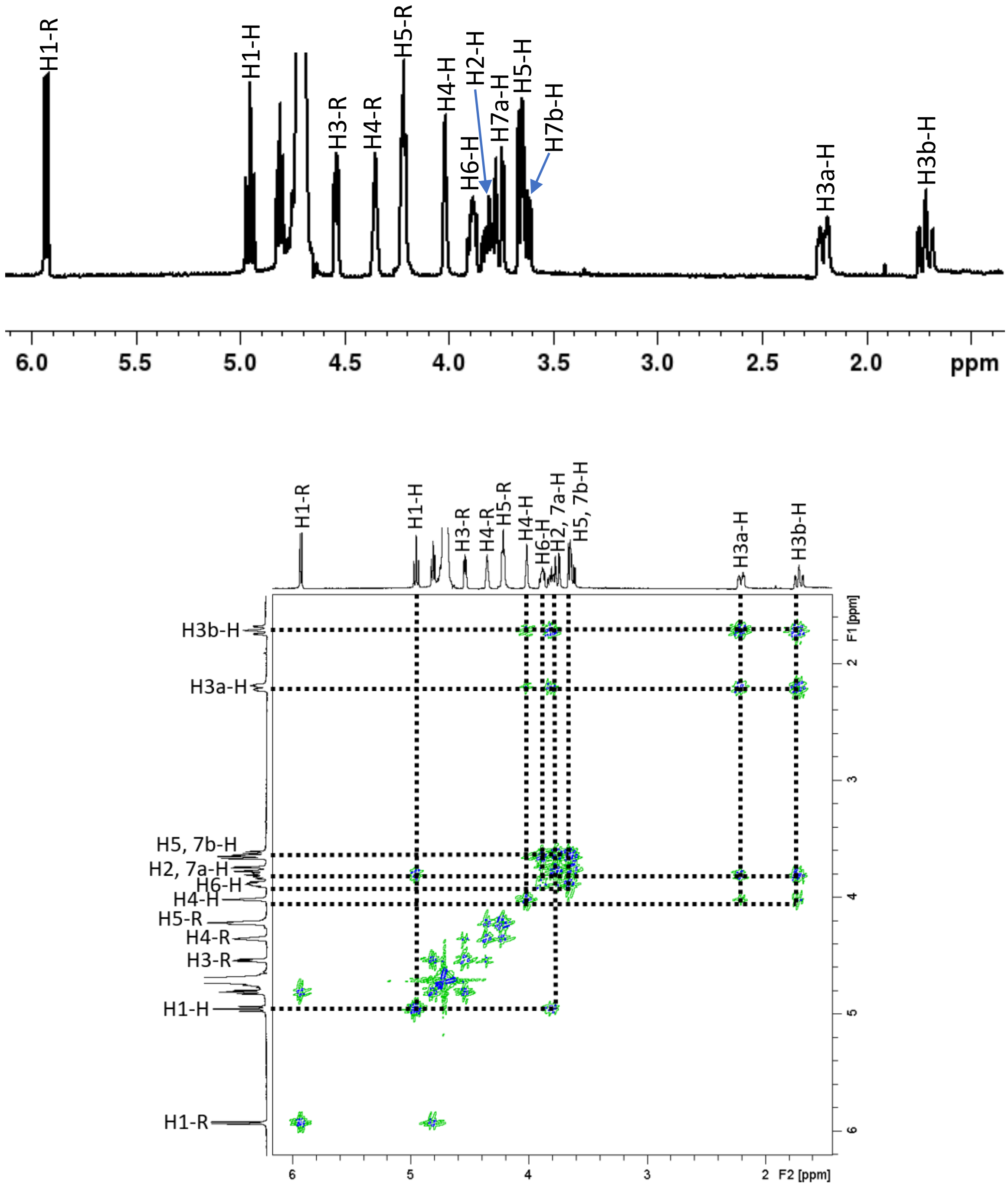

Figure S40. ^1H NMR and ^1H-^1H COSY NMR spectra of compound **38** formed by the reduction of compound **36** by the C4-reductase from HS:11 in H_2O. Resonances for the hydrogen labeled with an "R" correspond to the ribose moiety of GDP, while those labeled with an "H" correspond to those of the heptose moiety. (top) ^1H NMR spectrum. (bottom) ^1H-^1H COSY NMR spectrum.

Table S10. Isolated yields and mass spectral data for compounds prepared for this investigation.

Mass Spectrum	Compound number	Isolated Yield	ESI-MS $[M-1]^{-1}$	Related NMR Spectrum
Figure S41A	**5**	32%	634	Figure S24
Figure S41B	**5**	61%	634	Figure S25
Figure S42A	**22**	29%	632	none
Figure S42B	**26**	55%	636	Figure S26
Figure S43A	**28**	63%	634	Figure S27
Figure S43B	**28**	62%	635	Figure S28
Figure S44A	**29**	51%	634	Figure S29
Figure S44B	**29**	53%	636	Figure S30
Figure S45A	**27**	54%	634	Figure S31
Figure S45B	**27**	50%	635	Figure S32
Figure S46A	**30**	20%	634	Figure S33
Figure S46B	**30**	19%	636	Figure S34
Figure S47A	**28**	59%	634	Figure S35
Figure S47B	**28**	61%	635	Figure S36
Figure S48A	**34**	47%	618	Figure S37
Figure S48B	**35**	52%	618	Figure S38
Figure S49A	**37**	49%	618	Figure S39
Figure S49B	**38**	44%	618	Figure S40
Figure 50	**33**	61%	616	none

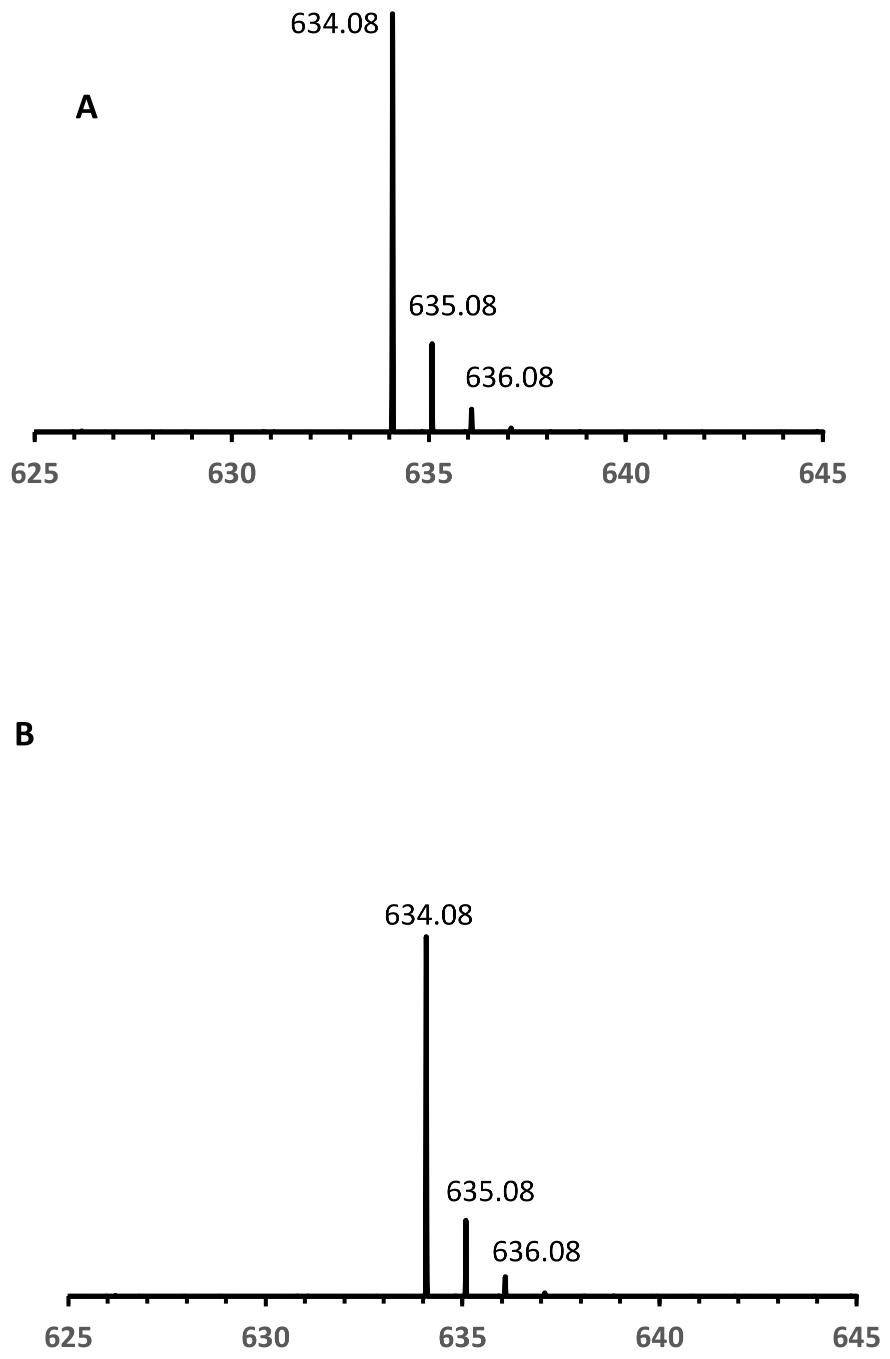

Figure S41. MS spectra of compound **5** (**A**), and **5** produced by the reduction of **22** with the C4-reductase from serotype HS:53 in H_2O (**B**)

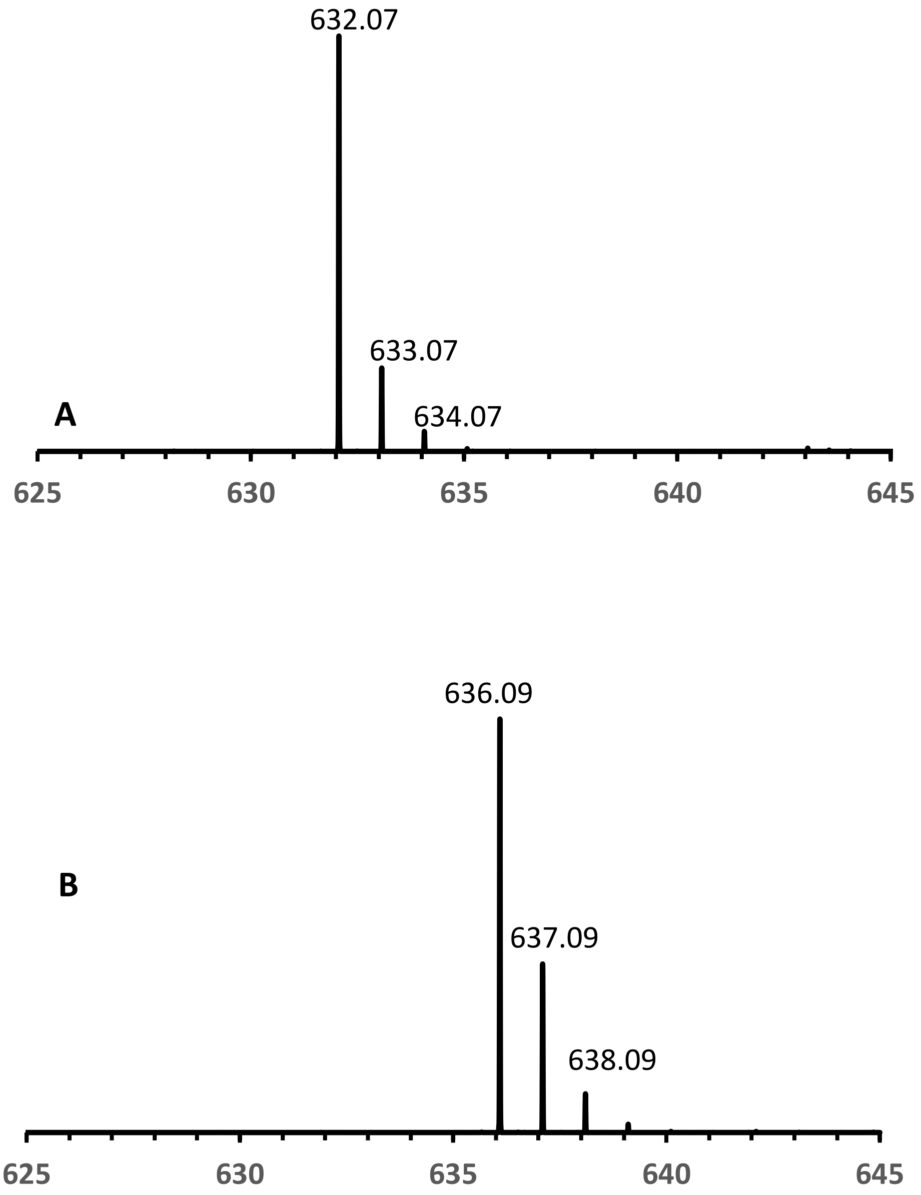

Figure S42. MS spectra of compound **22** (**A**) and of compound **26** formed from the reduction of **25** by the C4-reductase from serotype HS:2 in D_2O (**B**)

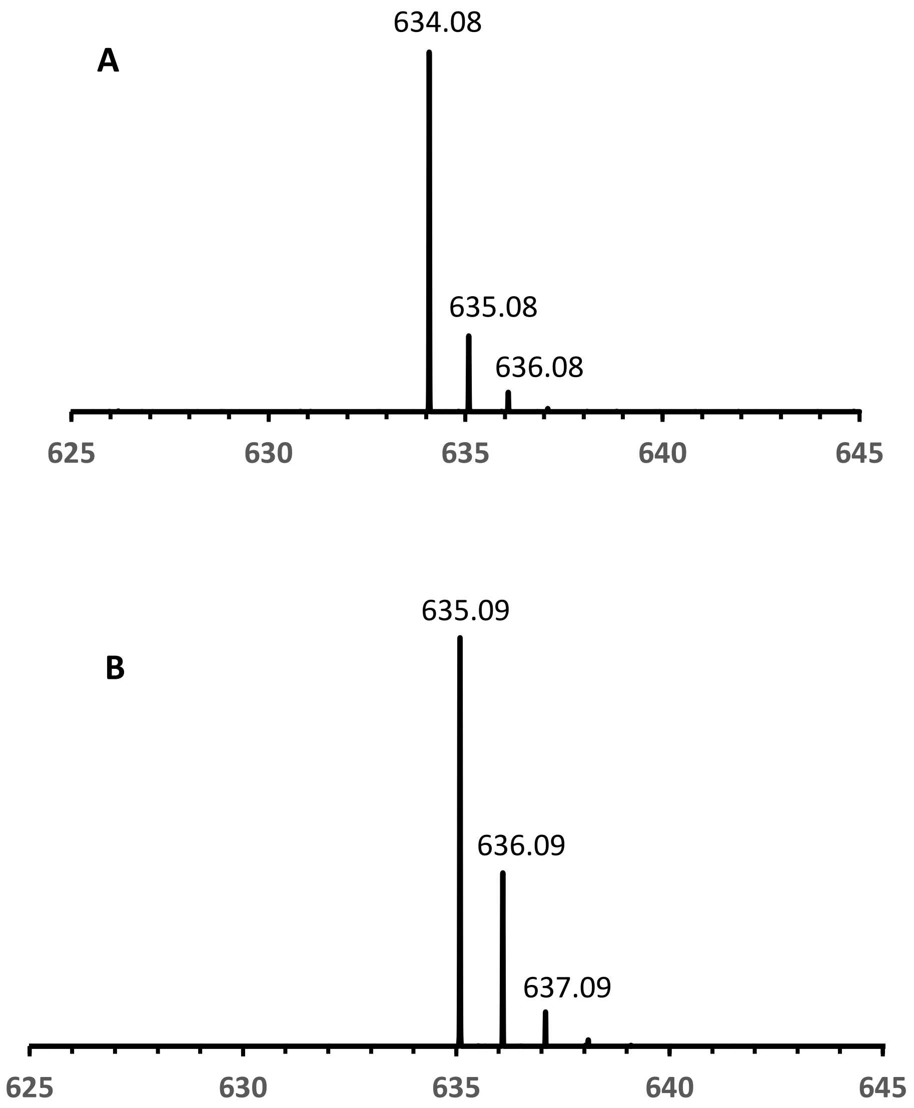

Figure S43. MS spectra of compound **28** formed from the reduction of **23** by the C4-reductase from serotype HS:3 in H_2O (**A**) and in D_2O (**B**)

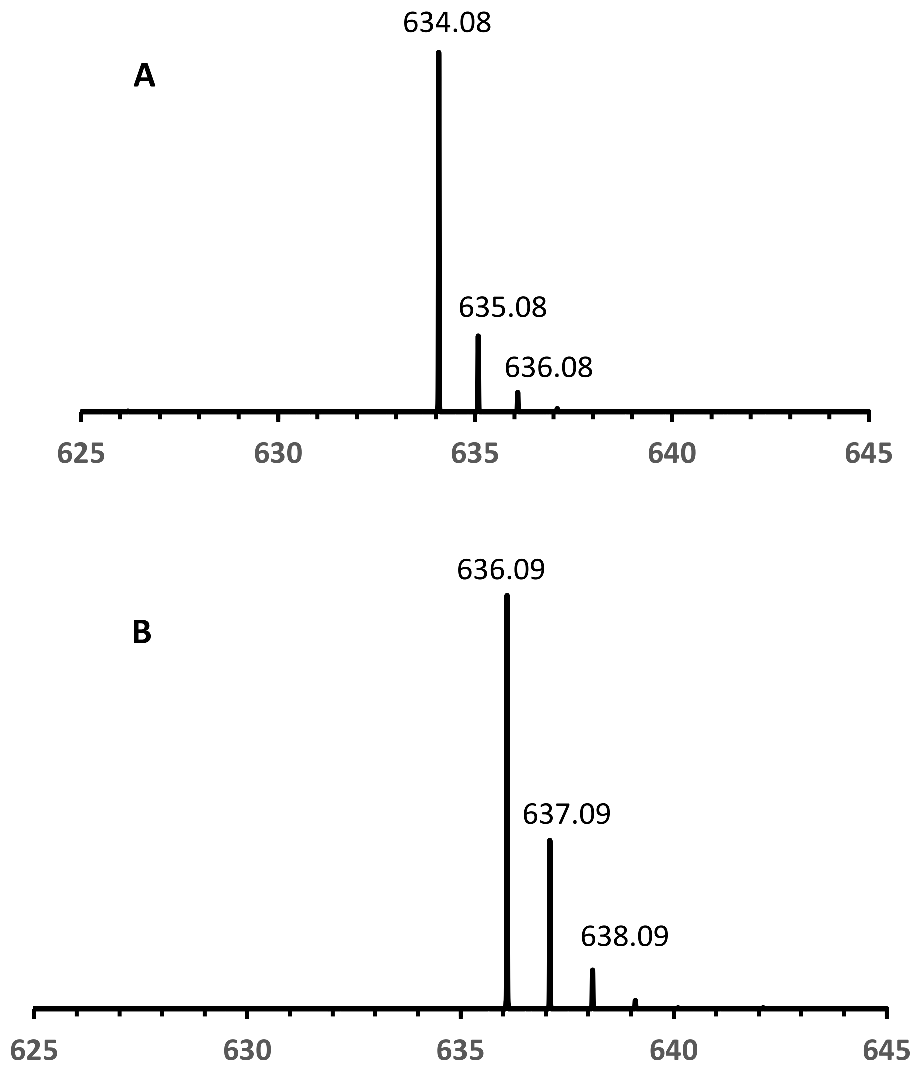

Figure S44. MS spectra of compound **29** formed from the reduction of **24** by the C4-reductase from serotype HS:15 in H_2O (**A**) and in D_2O (**B**)

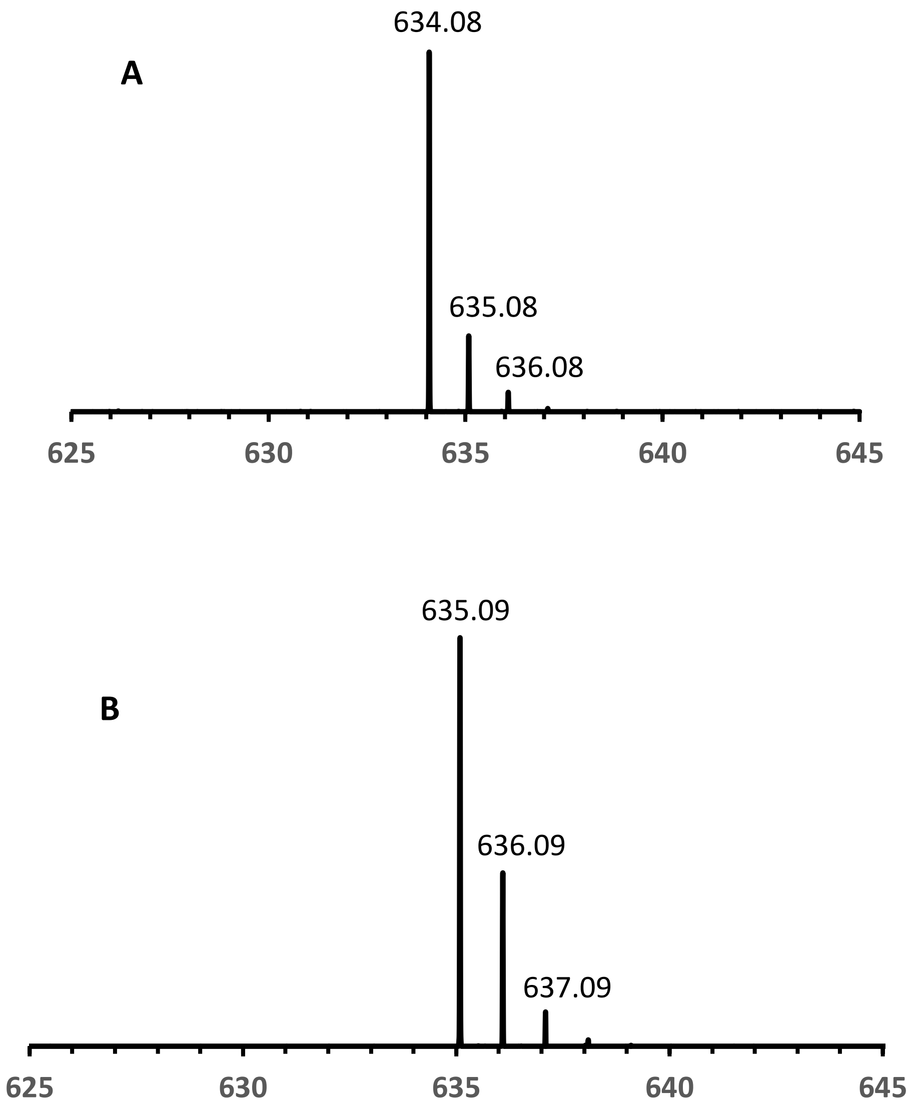

Figure S45. MS spectra of compound **27** formed from the reduction of compound **23** by the C4-reductase from serotype HS:23/36 in H_2O (**A**) and in D_2O (**B**)

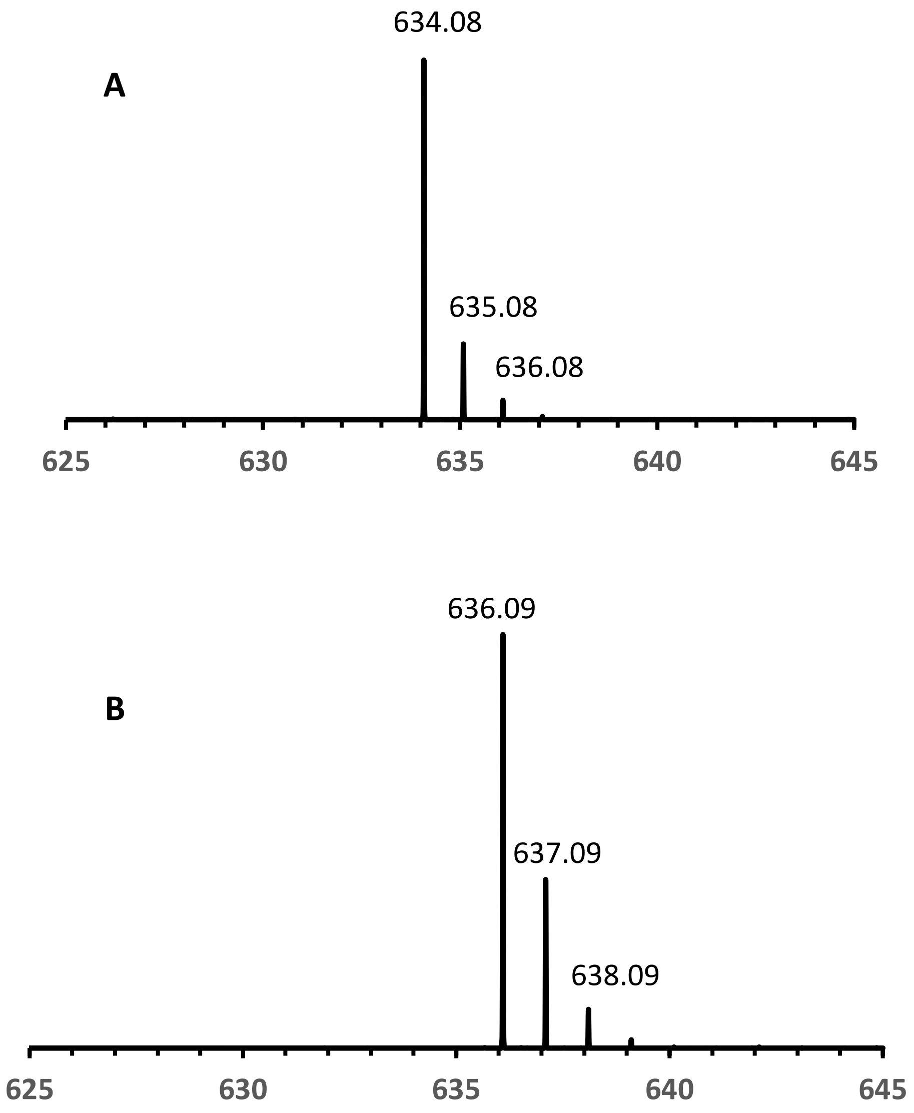

Figure S46. MS spectra of compound **30** formed from the reduction of compound **25** by the C4-reductase from serotype HS:42 in H_2O (**A**) and in D_2O (**B**)

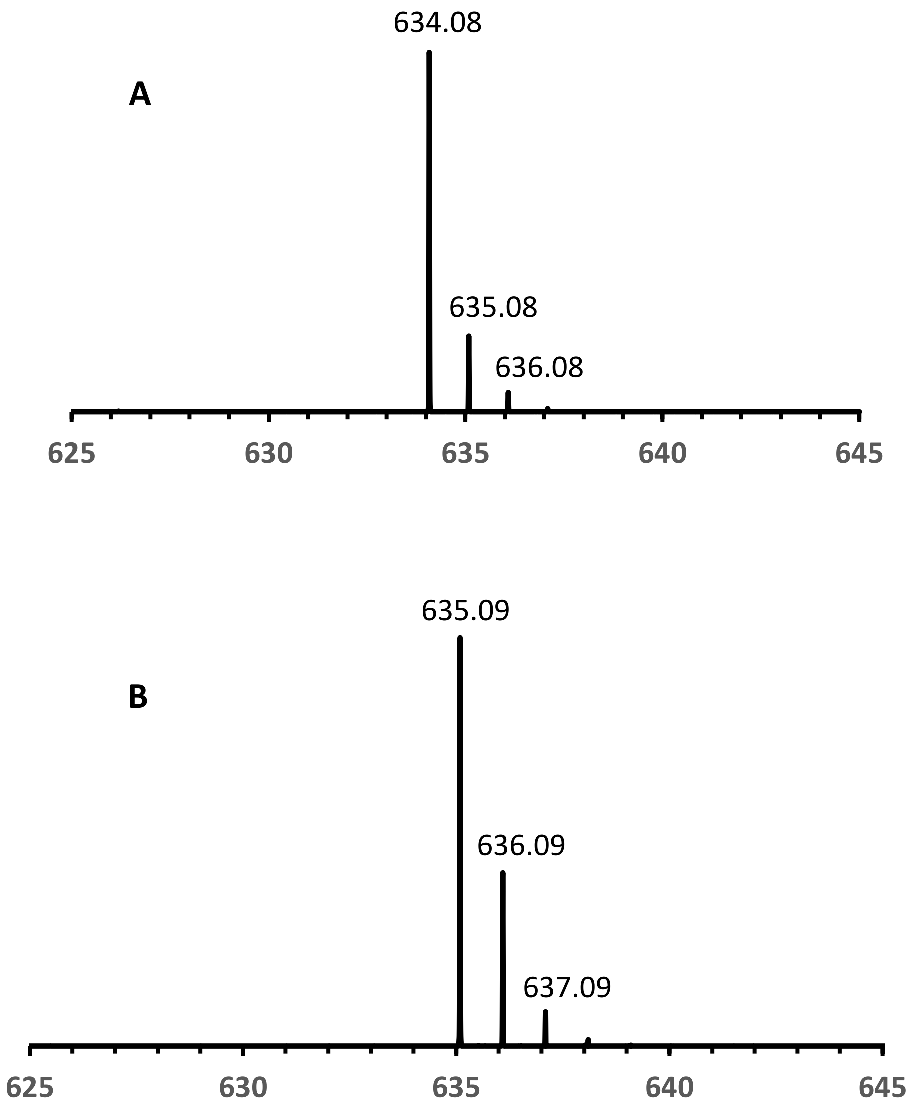

Figure S47. MS spectra of compound **28** formed from the reduction of compound **23** by the C4-reductase from serotype HS:33 in H_2O (**A**) and in D_2O (**B**)

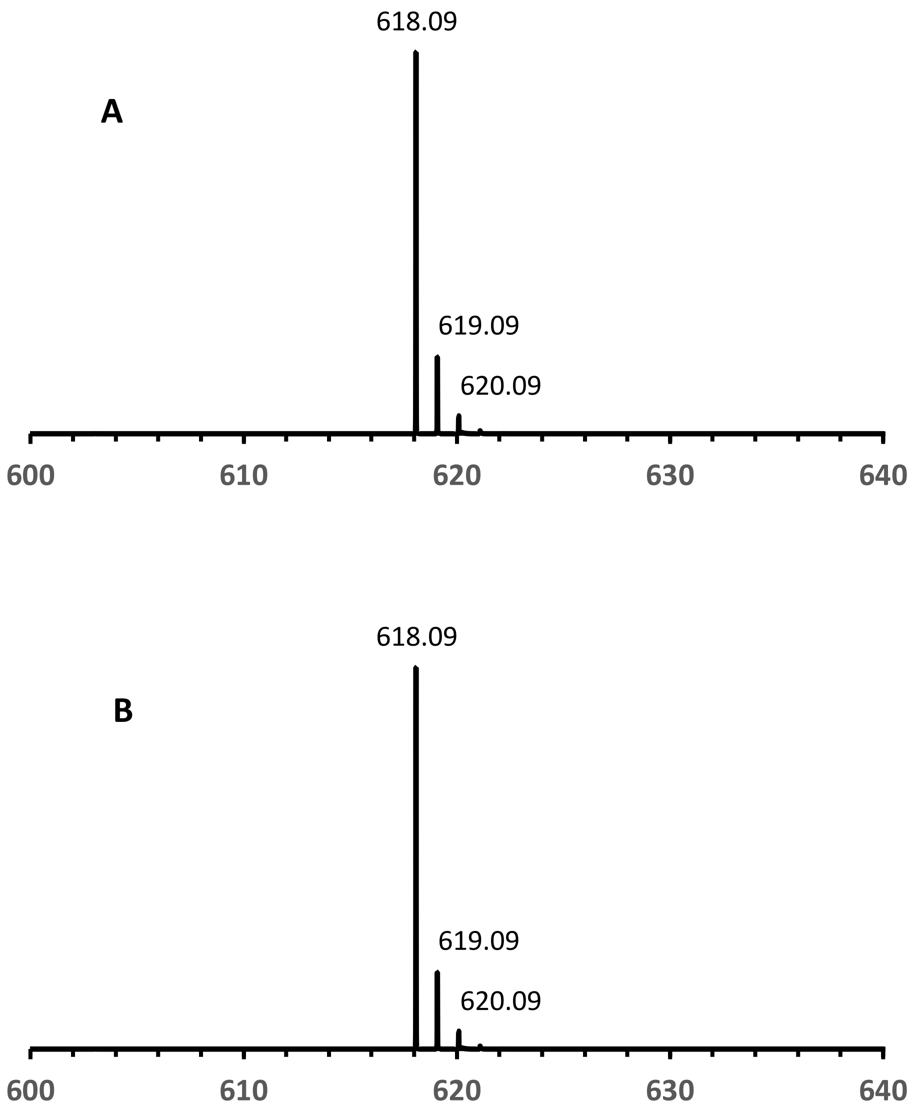

Figure S48. MS spectra of compound **34** formed from the reduction of compound **33** by the C4-reductase from serotype HS:53 in H_2O (**A**), and of compound **35** formed from the reduction of compound **33** by the C4-reductase from serotype HS:3 in H_2O (**B**).

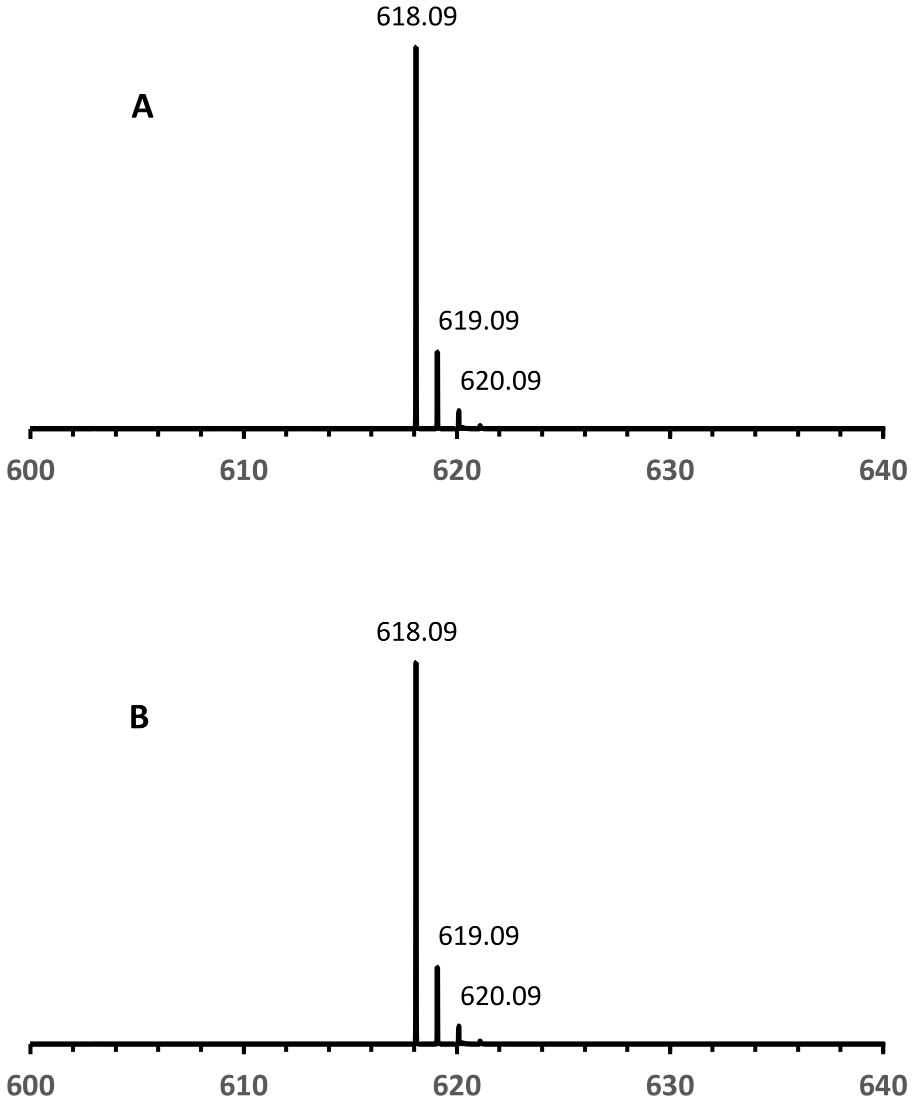

Figure S49. MS spectra of compound **37** formed from the reduction of compound **36** by the C4-reductase from serotype HS:2 in H_2O (**A**), and of compound **38** formed from the reduction of compound **36** by the C4-reductase from serotype HS:11 in H_2O (**B**).

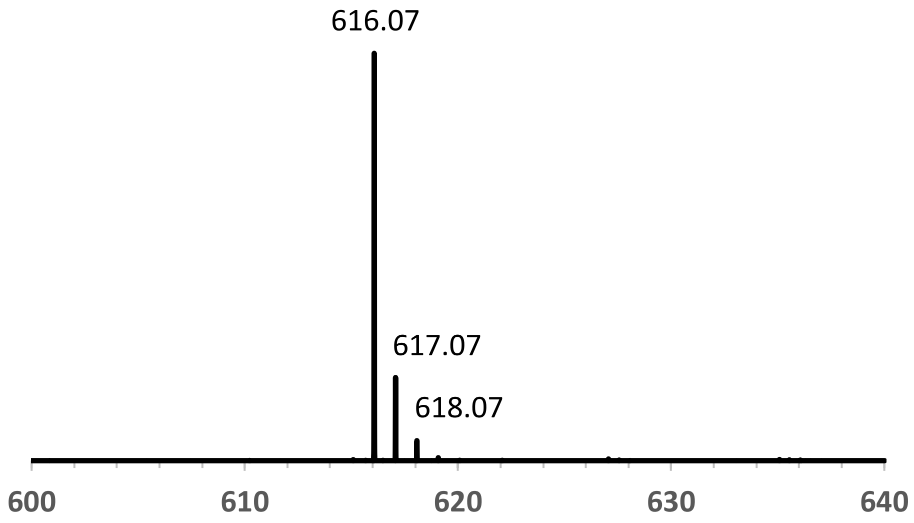

Figure S50. MS spectra of compound **33** made from compound **5** using the C4-dehydrogenase Cj1427 and the C3-dehydratase from HS:5.

References

1. Monteiro, M.A.; Noll, A.; Laird, R. M.; Pequegnat, B.; Ma, Z. C.; Bertolo, L.; DePass, C.; Omari, E.; Gabryelski, P.; Redkyna, O.; Jiao, Y. N.; Borrelli, S.; Poly, F.; Guerry, P. *Campylobacter jejuni* Capsule Polysaccharide Conjugate Vaccine. In Carbohydrate based Vaccines: from Concept to Clinic; *American Chemical Society: Washington, DC*, **2018**, pp. 249−271.
2. Ghosh, M. K.; Xiang, D. F.; Raushel, F. M. Biosynthesis of 3,6-Dideoxy-heptoses for the Capsular Polysaccharides of *Campylobacter jejuni*. *Biochemistry* **2023**, *62*, 1287-1297.
3. Xiang, D. F.; Ghosh, M. K.; Riegert, A. S.; Thoden, J. B.; Holden, H. M.; Raushel, F. M. Bifunctional Epimerase/Reductase Enzymes Facilitate the Modulation of 6-Deoxy-Heptoses Found in the Capsular Polysaccharides of *Campylobacter jejuni*. *Biochemistry* **2023**, *62*, 134-144.
